# High-order layered self-assembled multicavity metal–-organic capsules and anti-cooperative host–multi-guest chemistry†

**DOI:** 10.1039/d4sc01204f

**Published:** 2024-05-08

**Authors:** Kaixiu Li, Zhengguang Li, Jie Yuan, Mingzhao Chen, He Zhao, Zhiyuan Jiang, Jun Wang, Zhilong Jiang, Yiming Li, Yi-Tsu Chan, Pingshan Wang, Die Liu

**Affiliations:** a Department of Organic and Polymer Chemistry, Hunan Key Laboratory of Micro & Nano Materials Interface Science, College of Chemistry and Chemical Engineering, Central South University Changsha Hunan-410083 China; b Department Institute of Environmental Research at Greater Bay Area, Key Laboratory for Water Quality and Conservation of the Pearl River Delta, Ministry of Education, Guangzhou Key Laboratory for Clean Energy and Materials, Guangzhou University Guangzhou-510006 China; c School of Chemistry and Chemical Engineering, Henan Normal University Xinxiang Henan 453007 China; d Department of Chemistry, National Taiwan University Taipei 10617 Taiwan

## Abstract

The construction and application of metal–organic cages with accessible internal cavities have witnessed rapid development, however, the precise synthesis of complex metal–organic capsules with multiple cavities and achievement of multi-guest encapsulation, and further in-depth comprehension of host–multi-guest recognition remain a great challenge. Just like building LEGO blocks, herein, we have constructed a series of high-order layered metal–organic architectures of generation *n* (*n* = 1/2/3/4 is also the number of cavities) by multi-component coordination-driven self-assembly using porphyrin-containing tetrapodal ligands (like plates), multiple parallel-podal ligands (like clamps) and metal ions (like nodes). Importantly, these high-order assembled structures possessed different numbers of rigid and separate cavities formed by overlapped porphyrin planes with specific gaps. The host–guest experiments and convincing characterization proved that these capsules G2–G4 could serve as host structures to achieve multi-guest recognition and unprecedentedly encapsulate up to four C_60_ molecules. More interestingly, these capsules revealed negative cooperation behavior in the process of multi-guest recognition, which provides a new platform to further study complicated host–multi-guest interaction in the field of supramolecular chemistry.

## Abstract 2

Complex metal–organic capsules with large cavities (1–4 pockets) have been prepared and used as host structures for multi-guest recognition, displaying intriguing negative cooperation behavior within separate rigid cavities in the binding of C_60_.

## Graphical abstract

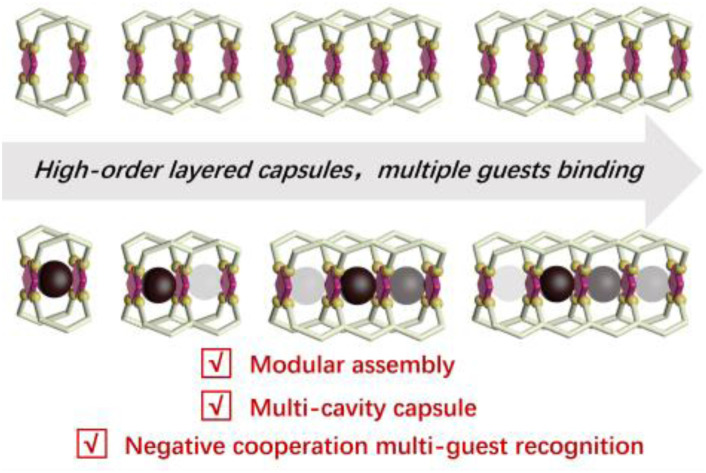

## Introduction

The pursuit of mimicking the structure and functionality of biological systems has prompted chemists to synthesize more elegant and complicated supramolecular assemblies.^1^ In this process, coordination-driven supramolecular assembly has become the preferred strategy due to its self-organization and predictability.^2–5^ A very diverse library of supramolecular architectures with well-defined shapes and sizes has been created, and pioneering contributions include metallo-macrocycles,^6–8^ platonic polyhedra,^9–11^ metal–organic cages,^12–14^ intricate molecule knots^15–17^ and so on.^18–20^ Highly sophisticated structures, such as the DNA double helix and virus capsid structure, can be constructed *via* supramolecular self-assembly in nature. However, the level of size and complexity of most artificial metal–organic supramolecular architectures employed to date lags far behind that of nature. Although remaining a formidable challenge, there are still creative efforts focused on developing construction strategies of complicated metal–organic supramolecules, such as low-symmetry metal–organic cages,^19–21^ interlocked molecular assemblies,^22–25^ giant metal-based supramolecules^13,26^ and so on.^7,27^

To achieve structural complexity and further diverse functionalities, the coordination-driven supramolecular cages may be the most suitable candidate due to the significant cavity, which can be exploited in diverse fields, such as biomedicine,^28^ separation,^29^ catalysis,^30,31^ and so on.^32^ However, most of the efforts have been focused on the metal–organic polyhedron featuring one single cavity.^33,34^ Therefore, the construction methods of metal–organic capsules with multiple cavities, namely multiple separate cavities in a single entity, are highly desirable.^35,36^ Three strategies are available to create multiple cavities in metallo-supramolecular architectures: interpenetrating architectures to increase the number of cavities,^37–39^ vertical extension of single cavity systems^40–45^ and expanding the structure on a two-dimensional plane.^46,47^ For example, Crowley *et al.* reported the first example of a [Pd_4_(L)_4_]^8+^ cage with three cavities using the long backboned multi-pyridine ligands.^43^ Soon after, Clever's group synthesized a peanut-shaped cage by assembling a tris-monodentate ligand with Pd^II^ cations, followed by quantitative catenation to give a five-cavity-containing compound.^37^ However, the previously reported multi-cavity supramolecular capsules can only recognize multiple anions or small molecules due to a small cavity volume.^43–45^ By comparison, those capsules being able to encapsulate large guests have rarely been documented due to the difficulties in synthesis and the assembly strategy.^42^ Moreover, the reports of cooperative behaviors of the homotropic binding or heterotropic binding guests in multi-cavity capsules remain elusive in spite of the fact that such behavior has been intensively investigated in a single cavity.^36^ Therefore, constructing capsules with multiple large cavities and further researching the mutual influence between the host and binding large guests is meaningful, yet especially challenging.

**Fig. 1:**
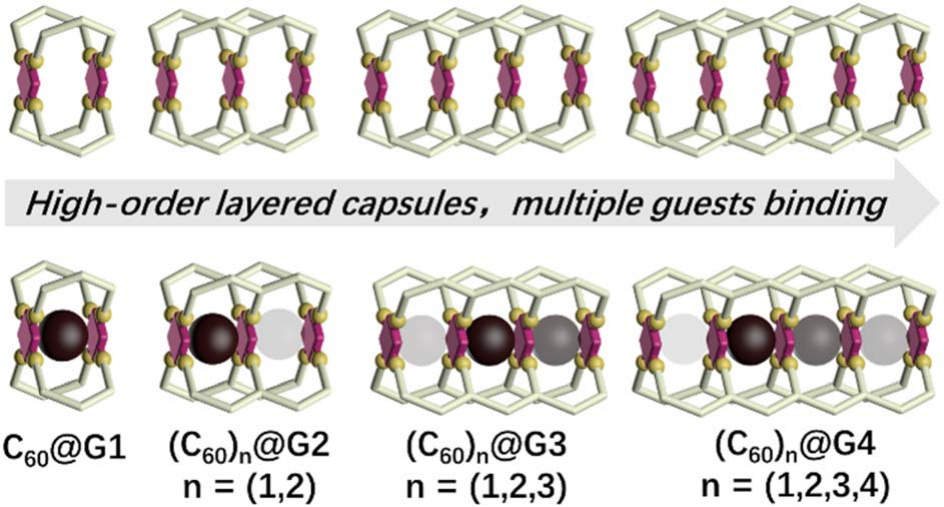
Cartoon representation of a series of multi-cavity cages and their C_60_ complexes (the darker colored ball represents a more prioritized binding).

Encouraged by previous excellent and appealing studies on multi-cavity cages, herein, a series of layered metal–organic capsules with large cavities varying from one to four pockets have been synthesized *via* the multicomponent self-assembly of terpyridine ligands and Cd^2+^. Furthermore, the large guest fullerene (C_60_) can be homogeneously bound by these capsules. Particularly noteworthy are the discrete single-molecular capsules that can wrap up to four C_60_ molecules (Fig. 1). More interestingly, based on the results of experimental and theoretical calculations, the negative cooperation effect between binding C_60_ has been also revealed.

## Results and discussion

### Assembly and characterization of metal–organic capsule G1

**Scheme 1:**
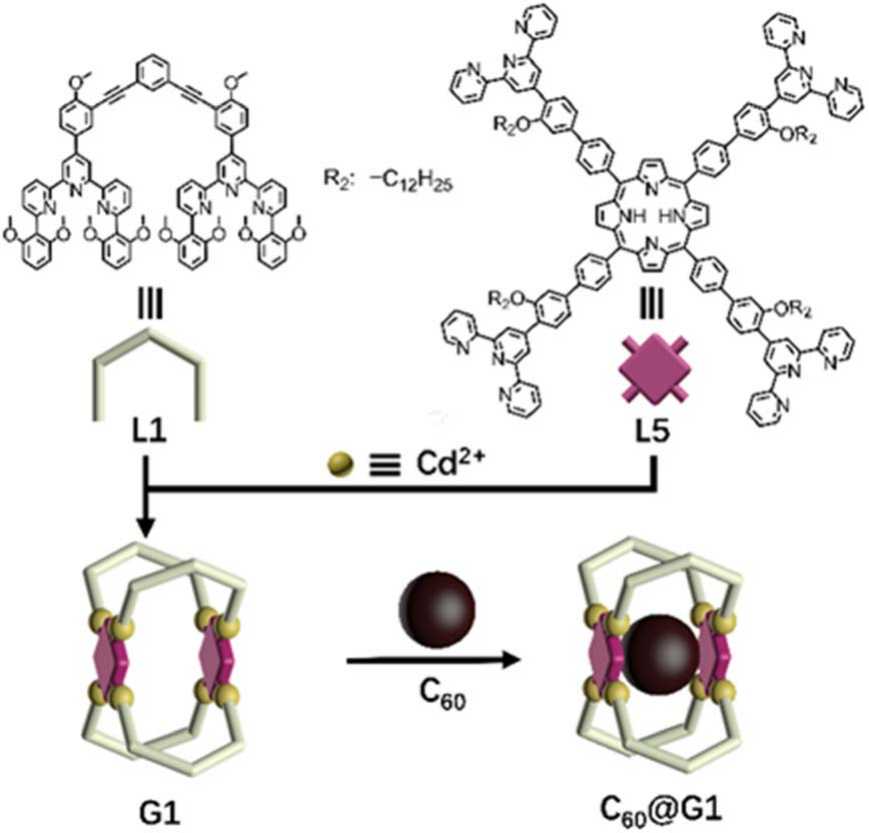
Chemical structure of ligands L1 and L5, and schematic illustration of the self-assembly and host–guest recognition to C_60_ of G1.

**Fig. 2:**
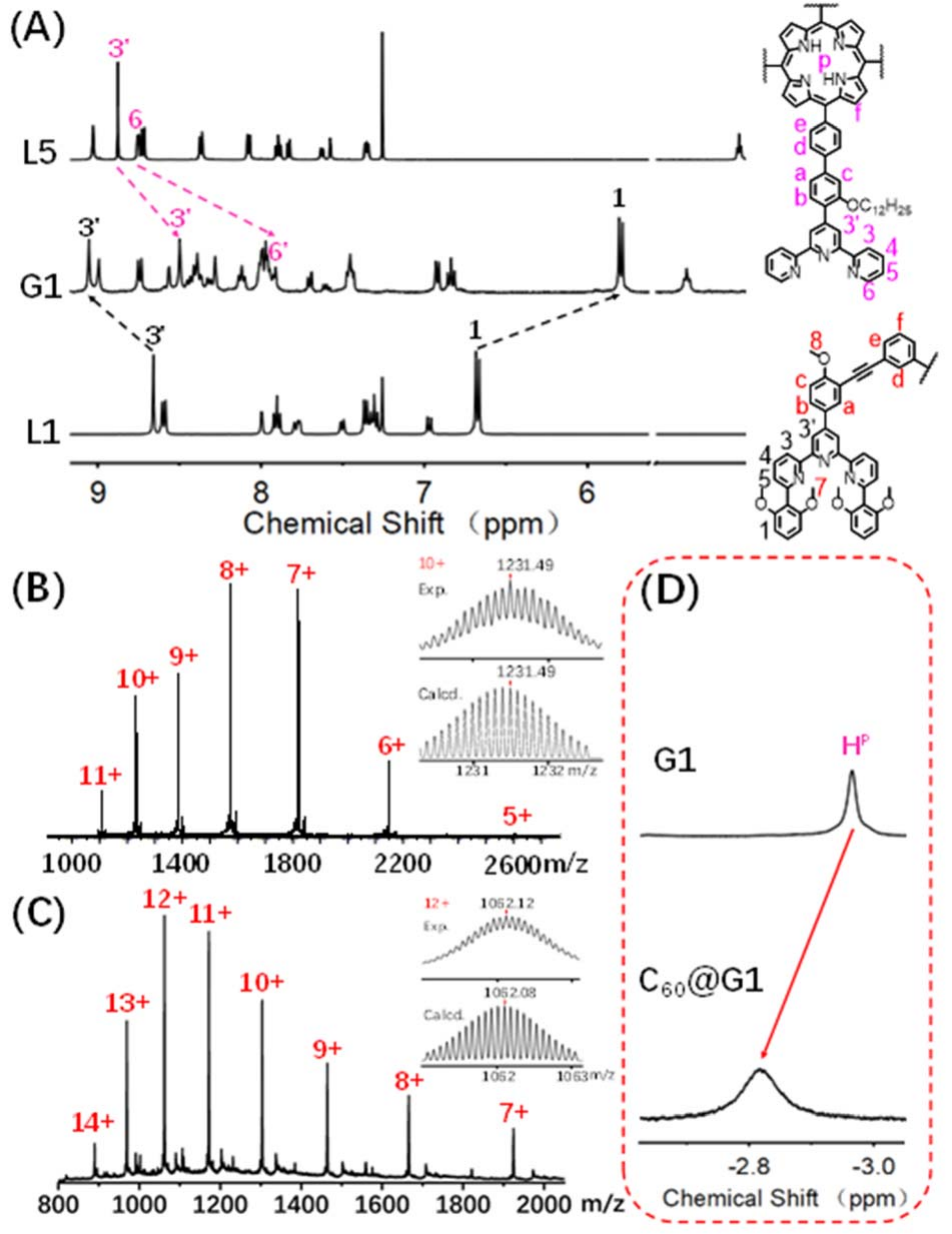
(A) ^1^H NMR spectra (500 MHz, 298 K) of supramolecule G1 in CD_3_CN and L1 and L5 in CDCl_3_; ESI-MS spectra of the (B) G1 and (C) C_60_@G1 (inset: isotopic distribution patterns); (D) partial ^1^H NMR spectra (600 MHz, 298 K) of G1 to highlight the shift of the proton H^P^.

Initially, as shown in Scheme 1, G1 with one cavity was designed. In order to avoid the self-sorting and realize the heteronuclear recognition in the process of coordination-driven assembly,^48^L1 modified with 2,6-dimethoxyphenyl as a steric hindrance group at the 6,6′′ position and L5 were obtained by Sonogashira and Suzuki coupling reactions, respectively, which were characterized using the ^1^H NMR, 2D COSY, 2D NOESY and ESI-MS spectra (Fig. 2A, S21–S24, S43–S46, S53 and S57†).

The assembly of G1 was carried out by mixing L1, L5 and Cd(NO_3_)_2_·4H_2_O at a precise stoichiometric ratio of 2 : 1 : 4 in MeOH/CHCl_3_ (1 : 1) and heating at 70 °C for 8 h (Scheme 1). After cooling to room temperature, an excessive CH_3_OH solution of NH_4_PF_6_ was added to exchange the anion NO_3_^−^ to PF_6_^−^, giving a deep purple precipitate. Based on the comparison of ^1^H NMR spectra of L1, L5 and supramolecular G1, the tpy-H^3′^ of ligand L1 shifted to the low-field (*δ* = 9.09 ppm, Δ*δ* = 0.43 ppm) due to the electron-withdrawing effect of the metal center after coordination with Cd^2+^. In contrast, the tpy-H^3′^ of ligand L5 shifted to high-field (*δ* = 8.53 ppm, Δ*δ* = 0.37 ppm) in G1 because of the strong shielding effect of 2,6-dimethoxyphenyl groups. These results indicated that L1 and L5 had formed heteronuclear coordination assembly (Fig. 2A). Moreover, two characteristic signals of tpy-H^3′^ displayed an integral ratio of 1 : 1, and the triple peak at 4.63 ppm combining with two singlet peaks at 4.17 and 2.77 ppm in the non-aromatic region, respectively attributed to the methylene and methoxyl groups, showed an integral ratio of 2 : 3 : 12, which was completely consistent with the desired structure (Fig. S62 and S63†). All proton signals were fully assigned by 2D COSY and 2D NOESY, verifying the successful formation of G1 (Fig. S64 and S65†). Through ESI-MS, a series of signal peaks from +11 to +5 corresponding to moieties of continuously losing various numbers of PF_6_^−^ were observed. And the experimental charge-to-mass ratio (*m*/*z*) values were consistent with the calculated ones (Fig. 2B and S80†). Further, TWIM-MS presented a group of signals ranging from +11 to +6, with a narrow drift time and no signal of other isomers, indicating that a single and discrete species G1 was formed (Fig. S79†).

### Host–guest interaction of G1 and C_60_

Based on previous reports, the porphyrin-containing cages were able to encapsulate large aromatic molecules,^49^ such as fullerene,^46,50–52^*via* suitable internal cavities and strong π–π interaction. With this in mind, the host–guest recognition of G1 was performed with C_60_ as the guest molecule in which the C_60_ solid (G1 : C_60_ molar ratio 1 : 3) was added to 0.6 mL (10.0 mg mL^−1^) CD_3_CN solution of capsule G1, and further heated at 80 °C for 24 hours. As shown in Fig. 2D and S94,† the characteristic signal of pyrrole protons (H^P^) in porphyrin rings was observed as one single peak and shifted from −2.93 ppm to low-field (−2.79 ppm) after the addition of C_60_, demonstrating that C_60_ was enveloped inside the cavity rather than on the periphery of G1.^53^ In order to further support the conclusion, the single-layered complex G0 was synthesized (Fig. S58–S61†). The ^1^H NMR signals of G0 clearly showed no change after mixing with C_60_ under the same conditions, manifesting that there was no obvious binding between single-layered porphyrin and C_60_ (Fig. S91†). Such results also excluded the possibility that C_60_ molecules were sitting outside each cavity and interacted with the external faces of the porphyrin walls in G1. Additionally, comparing the ^13^C NMR of C_60_@G1 with G1 in CD_3_CN, a sharp single peak at 140.4 ppm appeared and can be assigned to encapsulated C_60_, supported by the fact that the ^13^C NMR signal of the sole C_60_ cannot be collected due to the negligible solubility in CD_3_CN (Fig. S95†). Further, the ESI-MS displayed a set of charged peaks from +14 to +7, which perfectly matched with C_60_@G1 instead of (C_60_)_2_@G1 or empty G1 (Fig. 2C and S96†). But for the mixture of G0 and C_60_, no ESI-MS signals attributed to (C_60_)_*n*_@G0 were observed (Fig. S92†). All these pieces of evidence proved that one C_60_ was located in the cavity of G1. Next, rigorous measurements of the binding constant were conducted by UV-vis titration of G1 with C_60_ solution in DMF. The formation of the C_60_@G1 complex was characterized by a substantial decrease of band intensity at 442 nm and a successive increase at 425 nm in comparison with that of G1 itself (Fig. S118†). Further, the binding constant was calculated to be (2.9 ± 0.6) × 10^4^ M^−1^ in DMF on the basis of a 1 : 1 binding mode^54^ (Fig. S117–S119†). The kinetic process of G1 wrapping C_60_ was investigated through time-dependent ^1^H NMR experiments, in which the time to reach equilibrium state at 333 K and 353 K was determined to be approximately 17 h and 13 h, respectively. Then the activation energy (*E*_a_) to encapsulate C_60_ for G1 was calculated to be 35.32 kJ mol^−1^ according to the Arrhenius formula (Fig. S120–S123†). Finally, the total energies (*E*) of G1, C_60_@G1, and C_60_ were calculated by the semiempirical quantum mechanical GFN1-xTB method,^55^ resulting in Δ*E* = −23.47 kcal mol^−1^ from discrete G1 and C_60_ to complex C_60_@G1 (Fig. S127†). These results demonstrated that G1 had strong binding affinities, yet high activation energy to C_60_. The above-mentioned performances indicated that cage G1 possessed the suitable cavities and was a perfect host to encapsulate C_60_.

### Assembly and characterization of metal–organic capsules G2–G4

The successful assembly and host–guest interaction of G1 proved that the heteronuclear assembly strategy was feasible to construct host capsules. Therefore, the work returned to the original idea to obtain multi-cavity supramolecules by similar construction methods. Multilevel ligands L2–L4 were obtained by Sonogashira coupling reactions, respectively, which were characterized using the ^1^H NMR, 2D COSY, 2D NOESY and ESI-MS spectra (Fig. S25–S38, S54 and S56†).

**Fig. 3:**
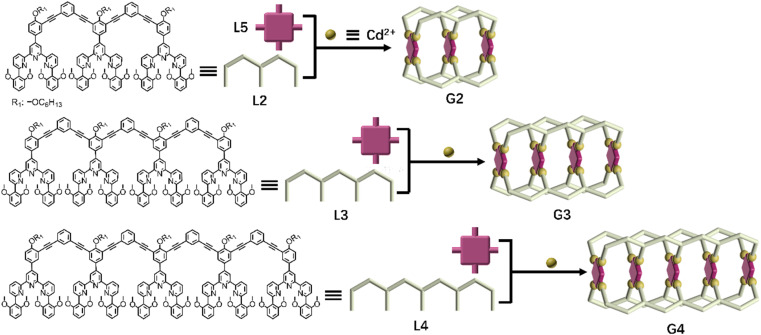
Chemical structure of ligands L2–L4 and schematic illustration of multicomponent self-assembly of multideck complex metal–organic capsules G2–G4.

**Fig. 4:**
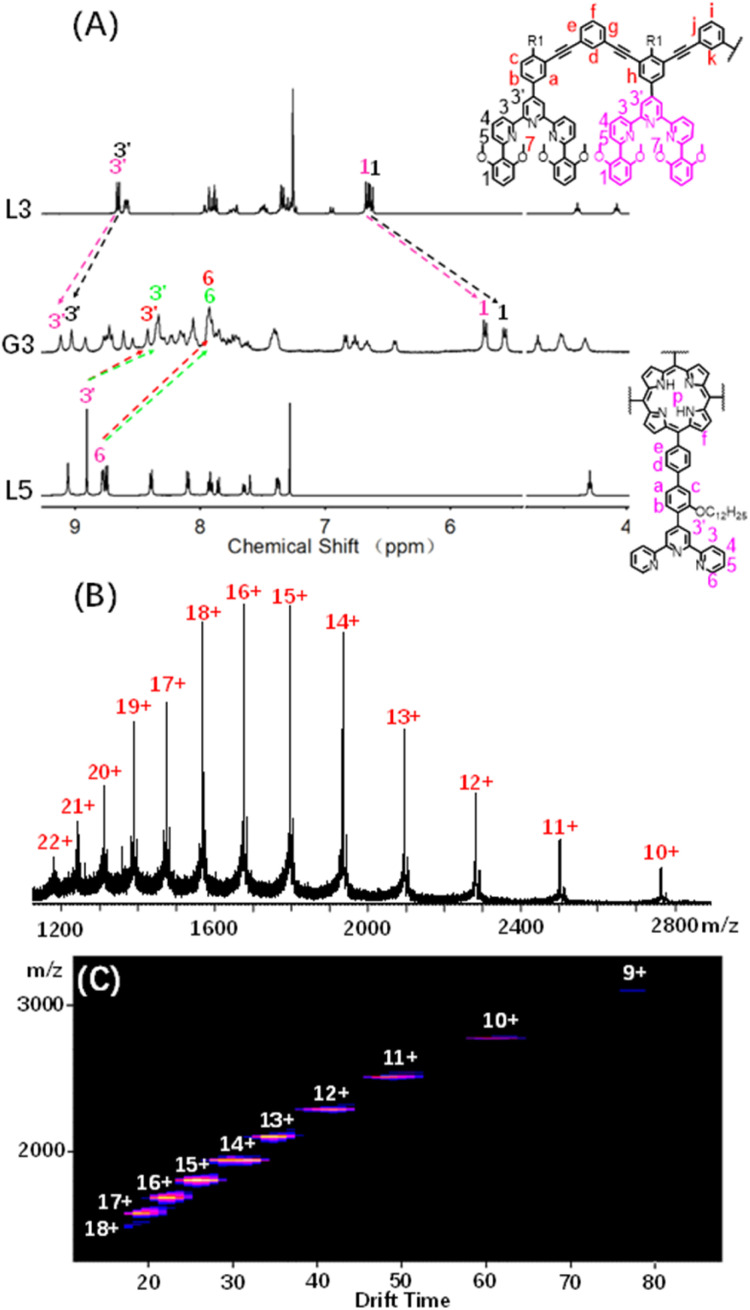
(A) ^1^H NMR spectra (500 MHz, 298 K) of G3 in CD_3_CN and L3 and L5 in CDCl_3_; (B) ESI-MS spectra and (C) 2D ESI-TWIM-MS plots of G3.

By using a similar method to G1, multi-cavity supramolecular capsules G2–G4 were obtained by heating the mixture of ligands L2/L3/L4, L5 and Cd(NO_3_)_2_·4H_2_O at accurate stoichiometric ratios (Fig. 3). The structural evidence of supramolecular G2–G4 was first collected by NMR experiments. Despite the large size and complicated composition of assembled architectures, sharp and distinct ^1^H NMR patterns were still obtained. From the comparison of G2–G4 with corresponding ligands (L2–L5), the signals assigned to the tpy-H^3′^ situated on ligands L2/L3/L4 showed low-field upon complexation, whereas the tpy-H^3′^ and the tpy-H6 located at ligand L5 obviously shifted to high-field due to the shielding effect of 2,6-dimethoxyphenyl, respectively, supporting the formation of complexes (Fig. 4A, S82 and S85†). Further exemplified with G3, as shown in Fig. 4A, structural information was collected: (i) four characteristic single peaks at 9.11, 9.03, 8.42 and 8.35 ppm attributed to tpy-H^3′^ with an integral ratio of 1 : 1 : 1 : 1 were observed; (ii) three triple peaks at 4.70 ppm, 4.52 ppm, and 4.34 ppm showed an integral ratio of 1 : 2 : 1 attributed to –OCH_2_–, and two single peaks at 2.68 and 2.51 ppm with an integral ratio of 1 : 1 attributed to –OCH_3_; (iii) two single peaks at −3.03 and −3.32 ppm with an integral ratio of 1 : 1 attributed to pyrrole proton H^P^ (Fig. S70 and S71†). The integral ratio of these characteristic peaks was completely consistent with the desired structure. Similarly, the detailed structural analysis of G2 and G4 was also presented in the ESI.† The attribution of all protons through the ^1^H NMR, 2D COSY and NOESY proved the successful assembly of the supramolecular cages G2–G4 (Fig. S66–S77†).

ESI-MS and TWIM-MS experiments were also performed to confirm the composition of G2–G4. A series of peaks with continuous mass charge ratio (*m*/*z*) from +16 to +7, +22 to +10 and +22 to +12 respectively correspond to charged moieties of G2 {[Cd_12_L2_4_L5_3_(PF_6_^−^)_24_]-*n*PF_6_^−^}^*n*+^ (*n* = 7–16) (Fig. S83A†), G3 {[Cd_16_L3_4_L5_4_(PF_6_^−^)_32_]-*n*PF_6_^−^}^*n*+^ (*n* = 10–22) (Fig. 4B) and G4 {[Cd_20_L4_4_L5_5_(PF_6_^−^)_40_]-*n*PF_6_^−^}^*n*+^ (*n* = 12–22) (Fig. S86A†). Further investigation revealed that the experimental *m*/*z* values matched well with theoretical values of G2 (with a molecular weight of 21 731.48 Da), G3 (with a molecular weight of 29 138.14 Da) and G4 (with a molecular weight of 36 544.80 Da), respectively. In addition, TWIM-MS spectra showed a series of bands with a narrowly distributed drifting time at each charge state from +16 to +7 (Fig. S83B†), +18 to +9 (Fig. 4C) and +21 to +16 (Fig. S86B†) for G2–G4, respectively. And no signals of other unexpected isomers were found, verifying accurate assembly of single and discrete species.

### 2D DOSY, TEM, and AFM characterization of G1–G4

**Fig. 5:**
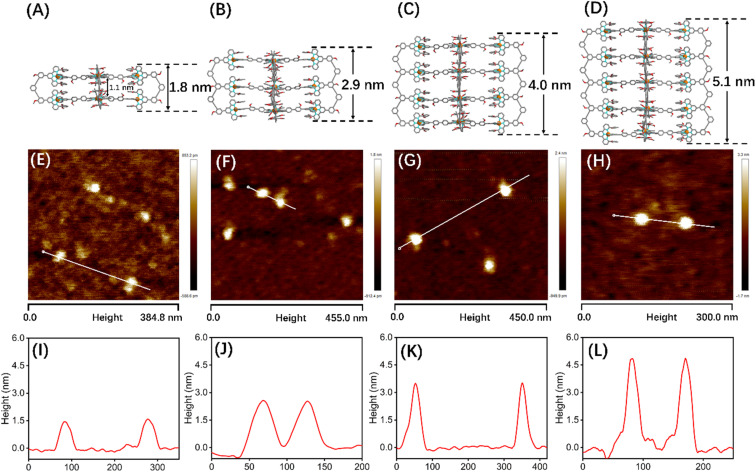
Structures from Materials Studio-Forcite-Geometry Optimization (the saturated fatty chain in G2–G4 was simplified to the methoxy group), AFM images and height mapping of the selected single molecule: (A), (E) and (I) for G1, (B), (F) and (J) for G2, (C), (G) and (K) for G3, and (D), (H) and (L) for G4.

Although many efforts have been devoted to cultivating single crystals, and even the crystals of G1 and C_60_@G1 were formed (Fig. S81†), unfortunately, no resolvable datum was obtained essentially due to large sizes and cavities with a large amount of unordered solvent molecules. In order to obtain more structural evidence of G1–G4, 2D DOSY, AFM and TEM characterization experiments were performed. The single band in the DOSY spectrum of G1–G4 confirmed that only one species was present in the solution. Furthermore, the diffusion coefficients from G1 to G4 increased sequentially, with log *D* = −9.70, −9.75, −9.77 and −9.91 m^2^ s^−1^, respectively (Fig. S87†). Subsequently, by calculating using the Stokes–Einstein equation, the experimental hydrodynamic radius was 3.0, 3.3, 3.5 and 4.8 nm, respectively, which matched the sizes of the molecular modeling structure (Fig. S88†). In addition, AFM experiments were carried out by spin-coating CH_3_CN solutions of G1–G4 (concentration of ∼10^−7^ M) onto the freshly cleaved mica surface and showed average vertical heights of 1.5 nm (Fig. 5A, E and I), 2.6 nm (Fig. 5B, F and J), 3.7 nm (Fig. 5C, G and K), and 4.8 nm (Fig. 5D, H and L), which is well consistent with the heights in molecular modelling, respectively. The measured width of these molecules from AFM profiles showed a large value because of the tip broadening effect.^56,57^ Meanwhile, the TEM images showed the dispersion of individual spots with average sizes fitting with the molecular models (Fig. S90†).

### Host–guest interaction of G2–G4 and C_60_

**Fig. 6:**
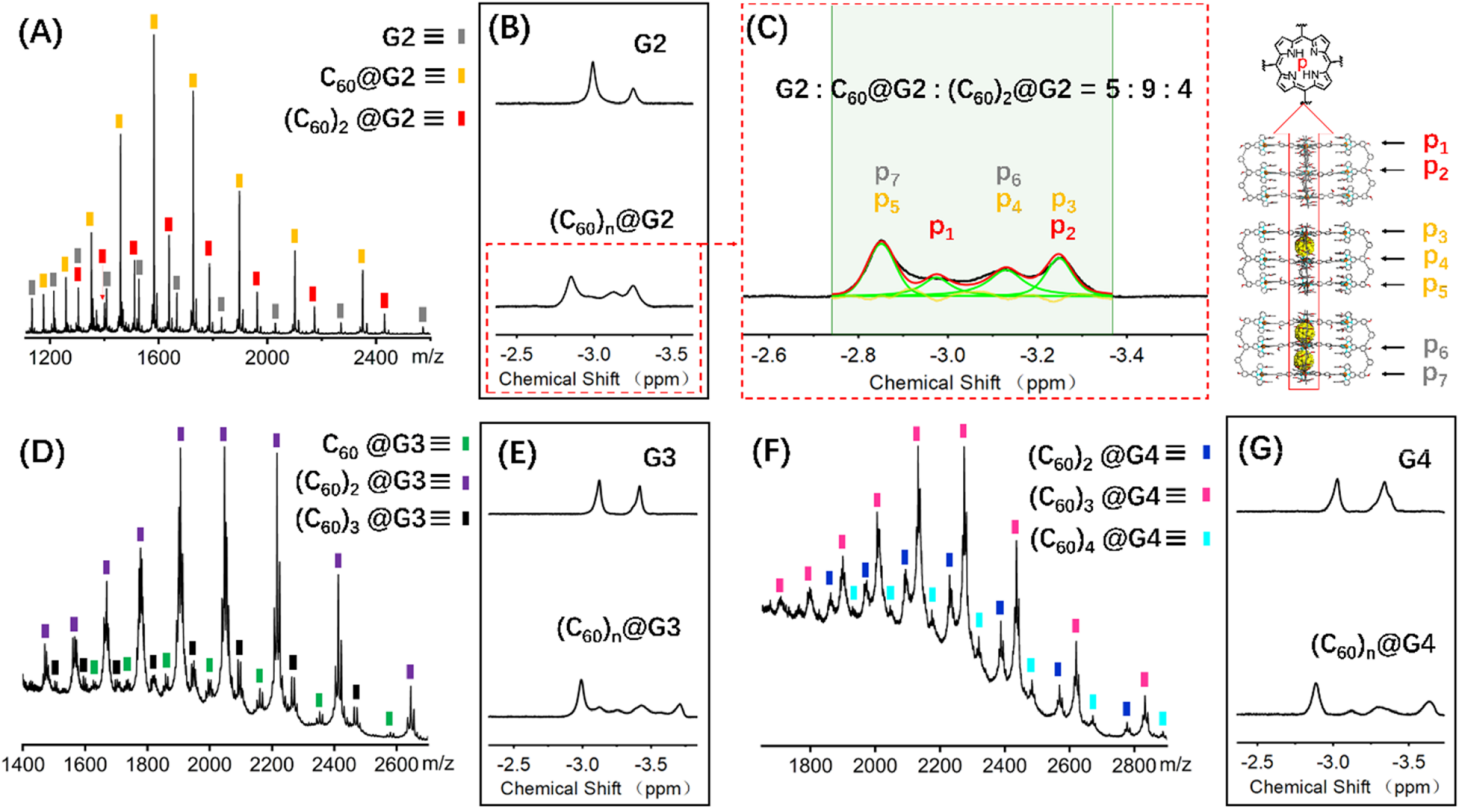
ESI-MS spectra of the (A) (C_60_)_*n*_@G2, (D) (C_60_)_*n*_@G3 and (F) (C_60_)_*n*_@G4, ^1^H NMR spectra (600 MHz, 298 K) comparison diagram of the H^P^ for (B) G2 and (C_60_)_*n*_@G2, (E) G3 and (C_60_)_*n*_@G3, (G) G4 and (C_60_)_*n*_@G4, (C) the ^1^H NMR spectra attribution of H^P^ for (C_60_)_*n*_@G2 and the proportion of G2, C_60_@G2 and (C_60_)_2_@G2.

Coupled with the above-mentioned host G1 being able to encapsulate C_60_ molecules, the successful synthesis of complicated multi-layered structures G2–G4 with various numbers of cavities make it feasible to research multi-guest recognition interactions, which have seldom been reported due to the synthetic obstacle.^42,58,59^ Hence, multiple C_60_ wrapping experiments were conducted, in which C_60_ solid [*n* (cavity): *n* (C_60_) = 1 : 3] was added to 0.6 mL 10.0 mg mL^−1^ CD_3_CN solution of capsules G2–G4, respectively. And further heating at 80 °C for 24 hours was performed to reach the thermodynamic equilibrium state, which was verified by the unchangeable ^1^H NMR signals (Fig. S101, S108 and S114†). Comparison of the ^1^H NMR spectra of G2–G4 in CD_3_CN before and after adding C_60_ molecules showed that the resonance signals of characteristic pyrrole H^P^ had obvious shifts, verifying the C_60_ encapsulation. However, complex multiple sets of peaks were present owing to the complex chemical environment of H^P^ after accommodating different numbers of C_60_ molecules in the cage cavities (Fig. 6B, E, G, S98, S106 and S112†).

**Fig. 7:**
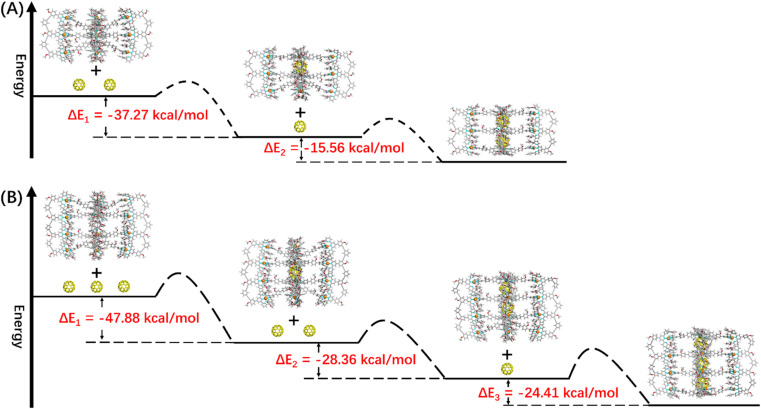
The energy schematic diagram of the inferred main path and the corresponding binding energy for (A) G2 and (B) G3 wrapping C_60_, respectively (the main path given by calculating the maximum energy difference).

In terms of host capsule G2, the host–guest recognition complex system (C_60_)_*n*_@G2 consisted of three species, namely G2, C_60_@G2 and (C_60_)_2_@G2, which was proved by NMR and ESI-MS. The ^1^H NMR spectrum showed that the H^P^ signals can be fitted to three sets, respectively corresponding to G2, C_60_@G2 and (C_60_)_2_@G2, and the integration ratio of G2, C_60_@G2 and (C_60_)_2_@G2 was determined to be 5 : 9 : 4, manifesting that C_60_@G2 was the dominant product (Fig. 6C and S99†). Meanwhile, compared with G2, the ^13^C NMR of (C_60_)_*n*_@G2 displayed a new broad single peak around 140.3 ppm composed of two slightly different signals belonging to C_60_, indicating that C_60_ was successfully wrapped into the cavity because C_60_ was originally nearly insoluble in acetonitrile (Fig. S100†). Moreover, as shown in Fig. 6A, the ESI-MS spectra in both CH_3_CN and DMF clearly showed three sets of consecutive peaks assigned to G2, C_60_@G2 and (C_60_)_2_@G2, respectively. And the content distribution of component G2, C_60_@G2 and (C_60_)_2_@G2 was further supported by the ESI-MS spectra (Fig. 6A and S102–S104†), which matched with the above-mentioned ^1^H NMR analysis. To shed light on the recognition process, by using similar methods to C_60_@G1, the binding constants of C_60_@G2 and (C_60_)_2_@G2 were determined by UV titration as follows: *K*_1_ = (3.06 ± 0.5) × 10^4^ M^−1^ and *K*_2_ = (1.99 ± 0.5) × 10^4^ M^−1^ (Fig. S124 and S125†). Meanwhile, the kinetic process of G2 wrapping C_60_ was monitored through time-dependent ^1^H NMR experiments at 353 K. These ^1^H NMR spectra showed that it took 18 h to achieve dynamic equilibrium of the (C_60_)_*n*_@G2 system (Fig. S101†), whereas just 13 h were required for C_60_@G1. Combining thermodynamics with kinetic data analysis, especially the cooperativity parameter (*α* = 0.65 < 1) in the process of G2 host–guest recognition (Fig. S125†),^60^ we rationally proposed that the encapsulation of two guest molecules is a negative cooperation process,^61^ namely one C_60_ molecule entering the host cavity relatively increases the difficulty of accommodating the other one. To further prove this hypothesis, the total energy of states G2, C_60_, C_60_@G2 and (C_60_)_2_@G2 were also calculated using the semiempirical quantum mechanical GFN1-xTB method, and the corresponding binding energy (Δ*E*) for sequentially wrapping two C_60_ was calculated to be −37.27 kcal mol^−1^ (G2 + C_60_ → C_60_@G2) and −15.56 kcal mol^−1^ (C_60_@G2 + C_60_ → (C_60_)_2_@G2) (Fig. 7A and S128†). Compared with the binding energy of C_60_@G1 (−23.47 kcal mol^−1^) (Fig. S127†), G2 revealed stronger binding affinities to the first C_60_, whereas the binding energy of further encircling the second one to form (C_60_)_2_@G2 remained relatively lower, supporting that negative cooperation encapsulation behavior of two guest molecules.

Moving to (C_60_)_*n*_@G3 and (C_60_)_*n*_@G4, in spite of overly complex resonance signals which cannot be specifically assigned due to the possible presence of multiple adducts for (C_60_)_*n*_@G3 and (C_60_)_*n*_@G4, respectively (Fig. S126†), the fact that G3/G4 packaged multiple C_60_ molecules can be supported by the multiple shifts of H^P^ in ^1^H NMR spectra in comparison to empty capsules G3/G4 (Fig. 6E, G, S106 and S112†). The asymmetric broad peaks belonging to C_60_ in their ^13^C NMR spectra further supported the encapsulation of multiple C_60_ by G3 and G4 (Fig. S107 and S113†). Convincingly, the ESI-MS spectra clearly revealed the composition of multiple components for (C_60_)_*n*_@G3 and (C_60_)_*n*_@G4, in which (C_60_)_*n*_@G3 mixtures contained the main component (C_60_)_2_@G3 in addition to a small portion of C_60_@G3 and (C_60_)_3_@G3 (Fig. 6D, S109 and S110†), and (C_60_)_*n*_@G4 mixtures consisted of consecutive three sets of signal peaks belonging to (C_60_)_2_@G4, (C_60_)_3_@G4, and (C_60_)_4_@G4, respectively, with (C_60_)_3_@G4 being the main component (Fig. 6F, S115 and S116†). Monitoring of the dynamic process of G3/G4 wrapping C_60_ at 353 K showed that G3/G4 wrapping C_60_ to reach dynamic equilibrium needed nearly 20 hours (Fig. S108 and S114†), manifesting the slower multiple binding process. Finally, the total energy of various possible products for (C_60_)_*n*_@G3 and (C_60_)_*n*_@G4 was also calculated (Fig. S129 and S130†). Based on the tendency of each step to generate the corresponding host–guest complexes with the lowest energy, the proposed processes of G3 and G4 wrapping multiple C_60_ and these corresponding binding energies (Δ*E*) were given (Fig. 7B and S131†). In the process of sequentially encapsulating C_60_, the binding energy revealed a downward trend and was the smallest for the last one which entered the cavities to form (C_60_)_3_@G3 and (C_60_)_4_@G4, which was calculated to be Δ*E*_3_ = −24.41 kcal mol^−1^ and Δ*E*_4_ = −14.24 kcal mol^−1^, respectively (Fig. 7B and S131†). Such results were in accordance with the wrapping behavior of G2, verifying the negative cooperation performance in the process of multiple host recognition to guest C_60_ molecules for multideck capsules G2–G4. The negative cooperation behavior can be attributed to conformational adaptation, in which the entry of large-size guest molecules affects the molecular configuration, resulting in the volume decrease of remaining cavities, thereby increasing the energy of the entire system after subsequent guest molecule binding.^42,62^ Similar collaborative behavior for the multi-guest recognition process in a single cavity was understandable because the electronic effects and spatial complementarity between multiple guests in a single-cavity host were strong.^51,63–65^ However, the synergistic effect of capsules with multiple separate cavities is rarely reported, only observed in a few capsules with flexible cavities and strong conformational adaptation,^66,67^ For herein reported capsules with rigid cavities, the negative cooperation behavior was possibly attributed to the large size of guest molecules and strong host–guest interactions.

In addition, the photosensitivity of capsule complexes with C_60_ ((C_60_)_*n*_@Gn) to generate singlet oxygen has been studied. The time-dependent UV-vis spectra under 405 nm irradiation were recorded and showed the gradually decreasing absorption intensity of DPHA, which can be ascribed to the formation of endoperoxide *via* singlet oxygen-mediated oxidation.^68^ Such a result indicates that capsules loaded with C_60_ possess photosensitive properties (Fig. S133 and S134†). As the number of cavities increases, the ability of capsules to generate ^1^O_2_ increases, principally because of improved photosensitive performance *via* enhanced light capture as the number of porphyrin rings increases (Fig. S135†). These results provide a method for designing and synthesizing supramolecular structures with enhanced photosensitivity in the future.

## Conclusions

Overall, using the multi-component modular self-assembly strategy, we have designed and synthesized a series of complex layered metal–organic capsules G1–G4. These multideck structures possess different numbers of large and separate internal cavities formed by two parallel porphyrin planes and displayed strong recognition capability for large guest molecule C_60_ (the binding constant up to 10^6^ M), convincingly supported by ^1^H NMR, ^13^C NMR, and ESI-MS spectroscopies, UV-vis titration and GFN1-xTB method calculation. Unprecedentedly, these multi-cavity capsules were exploited as multi-guest recognition systems and displayed intriguing binding performance up to four C_60_ at a time. Interestingly, the negative cooperation behavior was observed for the capsules G2–G4 in the process of binding multiple C_60_ molecules, which can be attributed to the scarce conformational adaptation of rigid capsules. This work provides a powerful strategy to construct complex metal–organic capsules with multiple cavities by multi-component coordination-driven self-assembly and establishes a novel platform for the study of host–guest interactions in multiple separate cavities, which lays a foundation for the construction of more complex host–guest systems in the future.

## Author contributions

K. L. conceived the project, synthesized the ligand molecules and completed the manuscript, which was supervised by D. L. and P. W. Characterization experiments including NMR, MS, AFM, and TEM were performed with the assistance of Z. L., M. C., J. W. and Z. J. All authors participated in the data analysis and discussions.

## Conflicts of interest

There is no conflict of interest to report.

## Supplementary Material

SC-015-D4SC01204F-s001
