# Supplementary material for: High-order layered self-assembled multicavity metal–-organic capsules and anti-cooperative host–multi-guest chemistry
Source: Chem Sci. 2024 May 8;15(23):8913–21. doi: 10.1039/d4sc01204f (PMC11168090; doi:10.1039/d4sc01204f)
Supplement: SC-015-D4SC01204F-s001 [file SC-015-D4SC01204F-s001.pdf]

Electronic Supplementary Material (ESI) for Chemical Science.
This journal is © The Royal Society of Chemistry 2024

High-order layered self-assembled multicavity metal-organic capsules and anti-cooperative host-multi-guest chemistry

Kaixiu Li,[a] Zhengguang Li,[a] Jie Yuan,[c] Mingzhao Chen,[b] He Zhao,[a] Zhiyuan Jiang,[a] Jun Wang,[b] Zhilong Jiang,[b] Yiming Li,[a] Yi-Tsu Chan,[d] Pingshan Wang,*[ab] Die Liu*[a]

[a] Department of Organic and Polymer Chemistry; Hunan Key Laboratory of Micro & Nano Materials Interface Science, College of Chemistry and Chemical Engineering; Central South University, Changsha, Hunan-410083, China

[b] Department Institute of Environmental Research at Greater Bay Area; Key Laboratory for Water Quality and Conservation of the Pearl River Delta, Ministry of Education; Guangzhou Key Laboratory for Clean Energy and Materials, Guangzhou University, Guangzhou-510006, China

[c] School of Chemistry and Chemical Engineering, Henan Normal University, Xinxiang, Henan 453007, China

[d] Department of Chemistry, National Taiwan University, Taipei 10617, Taiwan

*Corresponding Author(s) Email Address: chemwps@csu.edu.cn; chem-ld@csu.edu.cn.

Contents

1. General Procedures 2
2. Synthesis of ligand L1-L5 3
3. NMR and MS spectra of compounds 13
4. Self-assembly of G0-G4. 40
5. NMR, MS, TWIM-MS spectra, AFM and TEM of G0-G4. 43
6. The host-guest interaction between G0-G4 and C_{60}. 59
7. Calculation of binding constant (K_a), activation energy (E_a) and the energy state of host-guest complexes. 73
8. Comparison of the properties of capsules loaded with C_{60} to produce $^1O_2$. 82
9. References 84

1. General Procedures

All chemicals were purchased from commercial suppliers and used without further purification unless otherwise specified. Column chromatography was conducted using basic Al_2O_3 (Sinopharm Chemical Reagents Co. Ltd, 200-300 mesh) or SiO_2 (Qingdao Haiyang Chemical Co., Ltd, 200-300 mesh).

NMR spectra were recorded on a Bruker ADVANCE 400M, 500M and 600M NMR Spectrometer. ^{1}H NMR chemical shifts are reported in ppm downfield from tetramethylsilane (TMS) reference using the residual protonated solvent as an internal standard.

Mass spectra of complexes and ligands were determined on Waters Synapt G2 Mass Spectrometer with traveling wave ion mobility (TWIM) under the following conditions: ESI capillary voltage, 3.5 kV; cone voltage, 35 V; desolvation gas flow, 800 L/h. TWIM-MS was measured with IM traveling wave height, 25 V; and IM traveling wave velocity, 1000 m/s.

Atomic force microscopy (AFM) was conducted on a Bruker Dimension Icon AFM system with ScanAsyst and the data were processed by NanoScope Analysis version 1.5 (Bruker Software, Inc.). AFM samples were prepared by casting a sample solution (1×10^{-7} M) on a freshly cleaved mica surface.

Transmission electron microscope (TEM) images were recorded on a JEM 2100 transmission electron microscope operated at an accelerating voltage of 200 KV. TEM samples were prepared by drop-casting a sample solution (1×10^{-7} M) onto a carbon-coated copper grid and dried in vacuo for 24 h.

Molecular Modeling. Calculations were proceeded with Geometry Optimization and followed by Anneal in Forcite module of Materials Studio version 7.0 program (Accelrys Software, Inc.)., the counterions were omitted.

2. Synthesis of ligand L1-L5.

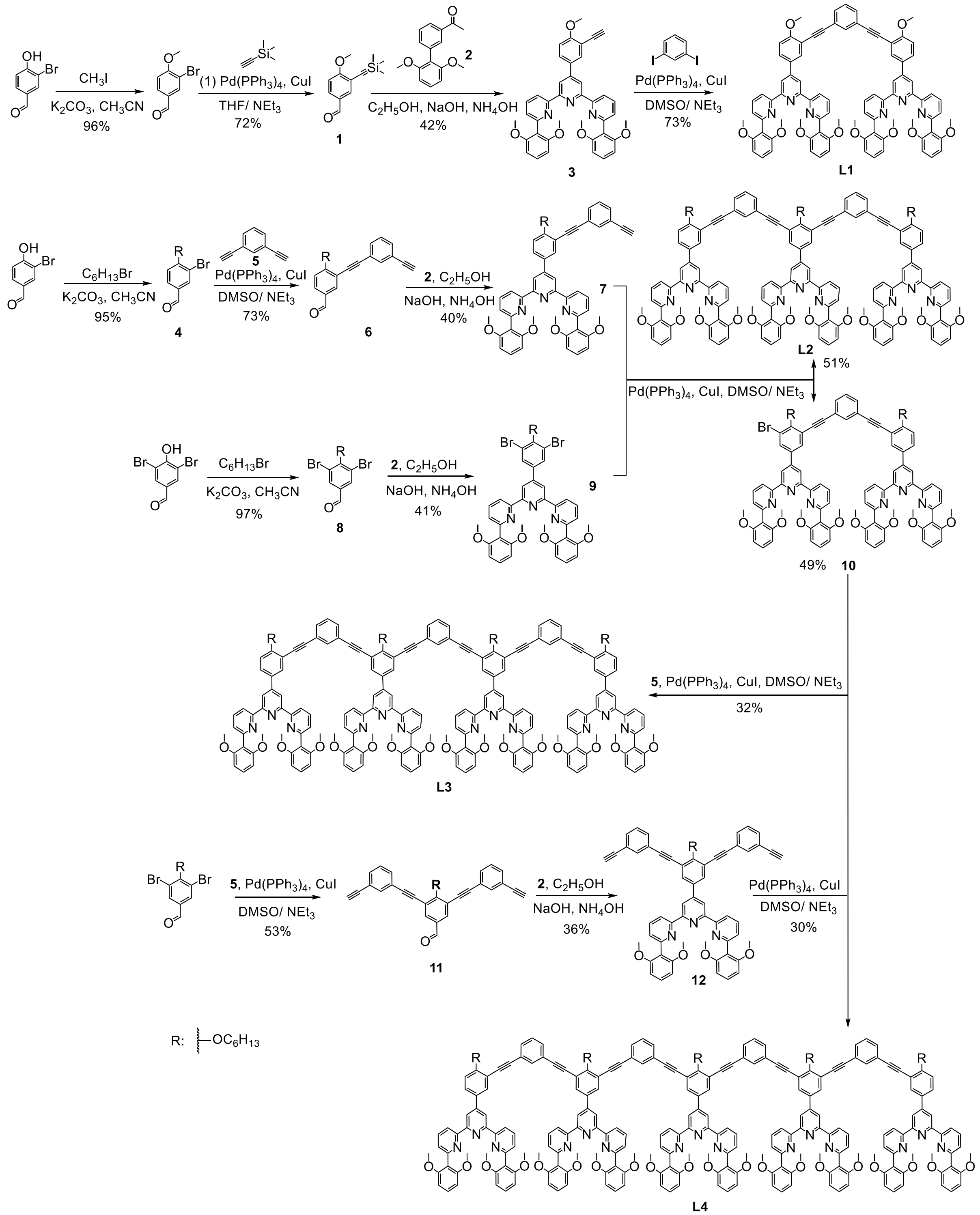

Scheme S1. Synthesis of ligands **L1-L4**.

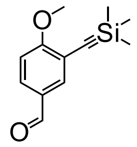

Compound 1. Dissolved 3-bromo-4-methoxybenzaldehyde (2.15 g, 10 mmol) and ethynyltrimethylsilane (1.20 g, 12 mmol) in 50ml of anhydrous tetrahydrofuran, then added 30ml of anhydrous triethylamine, then added CuI 15 mg and $Pd(PPh_3)_4$ (177 mg, 0.15 mmol), and finally let the whole reaction under the atmosphere of Ar gas 85 °C reaction for 12h. After the reaction was cooled to room temperature, silica gel powder was added and decompression distillation was conducted to obtain the sample, The intermediate product was obtained by chromatography column method, dissolved in the mixed solvent of $CHCl_3$/MeOH (1:1, v/v) and added with NaOH, stirred at room temperature for 2h, and finally extracted to obtain compound **1**. (1.60 g, 72%); ^{1}H NMR (400 MHz, $CDCl_3$) δ = 9.78 (s, 1H), 7.89 (s, 1H), 7.76-7.74 (d, J = 8 Hz, 1H), 6.91-6.89 (d, J = 8 Hz, 1H), 3.89 (s, 3H), 0.20 (s, 12H). ^{13}C NMR (126 MHz, $CDCl_3$, ppm) δ = 190.12, 164.80, 136.09, 131.86, 129.54, 113.41, 110.78, 100.19, 99.62, 56.33.

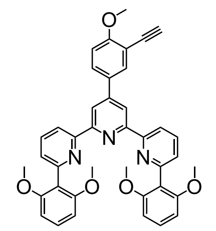

Compound 3. Compound **1** (2.20 g, 10 mmol), compound **2** (5.63 g, 22 mmol) and NaOH (1.60 g, 40 mmol) were mixed in 80ml EtOH and stirred at room temperature for 1 day. Then 40ml $NH_3{\cdot}H_2O$ was added to reflux for one day at 85 °C. After the reaction, it was cooled to room temperature and precipitation would occur. The solid was pumped out and recrystallized with $CHCl_3/CH_3OH$ to give **3** as a white solid (2.67 g, 4.20 mmol) in 42% yield. ^{1}H NMR (500 MHz, 298 K, $CDCl_3$, ppm) δ= 8.65 (s, 2H), 8.62-8.61 (d, J = 5 Hz, 2H), 7.98 (s, 1H), 7.95-7.92 (t, 2H), 7.83-7.81 (d, 1H), 7.40-7.37 (t, 4H), 6.98-6.97 (d, J = 5 Hz, 1H), 6.74-6.73 (d, 4H), 3.96 (s, 3H), 3.80 (s, 12H), 3.34 (s, 1H). ^{13}C NMR (126 MHz, 298 K, $CDCl_3$) δ = 160.93, 158.38, 156.45, 156.18, 153.86, 148.18, 136.42, 133.05, 131.41, 129.63, 129.39, 126.23, 119.82, 119.55, 118.72, 111.48, 110.83, 104.63, 81.35, 79.86, 56.19, 56.04.

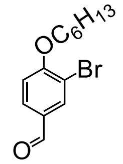

Compound 4. To a flask containing 3-bromo-4-hydroxybenzaldehyde (5.02 g, 25 mmol), 1-bromohexane (8.25 g, 50 mmol) and K_2CO_3 (6.90 g, 50 mmol), add 60ml CH_3CN as solvent. The system was pumped and backfilled with Ar. Then the mixture was refluxed for 1 days under Ar. After cooled to 25 °C, the K_2CO_3 in the reaction solution was filtered out, the filtrate was decompressed and distilled to obtain the colorless liquid compound **4** (6.77 g, 23.7 mmol) in 95% yield. ^{1}H NMR (400 MHz, 298 K, $CDCl_3$, ppm) δ= 9.83 (s, 1H), 8.08 (s, 1H), 7.81-7.78 (d, *J* = 12

Hz, 1H), 6.99-6.97(d, J = 8 Hz, 1H), 4.13-4.10 (t, 2H), 1.91-1.86 (m, 2H), 1.54-1.50 (m, 2H), 1.38-1.35 (m, 4H), 0.93-0.90 (m, 3H). ^{13}C NMR (101 MHz, 298 K, $CDCl_3$, ppm) δ = 156.34, 155.98, 150.01, 149.19, 147.23, 136.80, 136.68, 131.57, 126.98, 123.75, 121.32, 119.14, 64.79.

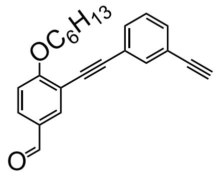

Compound 6. To a flask containing compound **4** (1.71 g, 6.0 mmol) and 1,3-diethynylbenzene (**5**) (1.50 g, 12 mmol), a mixed solvent (50 mL) of THF/Et_3N (3:2, v/v) was added. After $Pd(PPh_3)_4$ (212 mg, 0.18 mmol) and CuI 15 mg was added into the mixture, the system was pumped and backfilled with Ar. Then the mixture was refluxed for 10 h under Ar. After cooled to 25 °C, the mixture was extracted with CH_2Cl_2 and the combined organic extract was evaporated to dryness under reduced pressure. Then subjected to column chromatography (SiO_2, CH_2Cl_2/hexane = 1:4) to give **6** as a white solid (1.44 g, 4.38 mmol) in 73% yield. ^{1}H NMR (400 MHz, 298 K, $CDCl_3$, ppm) δ = 9.90 (s, 1H), 8.03 (s, 1H), 7.87-7.85 (d, J = 8 Hz, 1H), 7.68 (s, 1H), 7.54-7.52 (d, J = 8 Hz, 1H), 7.49-7.48 (d, J = 4 Hz, 1H), 7.36-7.33 (t, 1H), 7.03-7.01 (d, J = 4 Hz, 1H), 4.18-4.14 (t, 2H), 3.13 (s, 1H), 1.94-1.91 (m, 2H), 1.60-1.57 (m, 2H), 1.43-1.36 (m, 4H), 0.94-0.91 (m, 3H). ^{13}C NMR (101 MHz, 298 K, $CDCl_3$, ppm) δ = 135.94, 135.64, 132.86, 132.72, 132.38, 128.61, 128.44, 122.73, 122.50, 122.06, 82.59, 82.40, 80.76, 78.25, 77.96, 74.39.

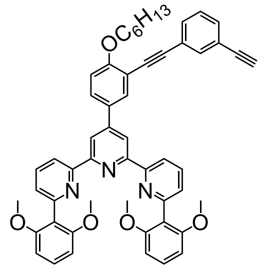

Compound 7. To a flask containing compound **6** (2.64 g, 8.0 mmol), compound **2** (4.42 g, 17.6 mmol) and NaOH (1.28 g, 32 mmol), add 80ml EtOH as solvent and stirred at room temperature for 1 day. Then 40ml $NH_3{\cdot}H_2O$ was added to reflux for one day at 85 °C. After the reaction, it was cooled to room temperature and precipitation would occur. The solid was pumped out and recrystallized with $CHCl_3$/CH_3OH to give **7** as a white solid (2.58 g, 4.20 mmol) in 40% yield. ^{1}H NMR (500 MHz, $CDCl_3$, ppm) δ = 8.65 (s, 2H), 8.60-8.59 (d, J = 4 Hz, 2H), 7.97-7.96 (d, J = 4 Hz, 1H), 7.93-7.89 (t, 2H), 7.77-7.75 (d, J = 4 Hz, 2H), 7.65 (s, 1H), 7.51-7.49 (d, J = 8 Hz, 1H), 7.44-7.42 (d, J = 8 Hz, 1H), 7.38-7.34 (t, 3H), 7.32-7.28 (t, 2H), 6.96-6.94 (d, J = 8 Hz, 1H), 6.72-6.70 (d, J = 8 Hz, 4H), 4.10-4.07 (t, 2H), 3.78 (s, 12H), 3.09 (s, 1H), 1.89-1.86 (m, 2H), 1.55-1.53 (m, 2H), 1.40-1.35 (m, 4H), 0.92-0.88 (m, 3H). ^{13}C NMR (101 MHz, $CDCl_3$, ppm) δ =164.99, 160.23, 158.40, 156.41, 153.79, 148.53, 136.49, 135.10, 132.21, 131.84, 131.58, 129.69, 128.37, 126.25, 124.12, 122.37, 119.60, 118.87, 112.84, 112.09, 104.68, 92.52, 86.63, 82.92, 68.88, 56.21, 31.64, 29.15, 25.81, 22.60, 14.07.

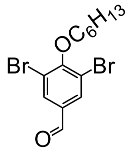

Compound 8. To a flask containing 3,5-dibromo-4-hydroxybenzaldehyde (5.60 g, 20 mmol), 1-bromohexane (6.60 g, 40 mmol) and K_2CO_3 (6.90 g, 50 mmol), add 60 ml CH_3CN as solvent. The system was pumped and backfilled with Ar. Then the mixture was refluxed for 1 days under Ar. After cooled to 25 °C, the K_2CO_3 in the reaction solution was filtered out, the filtrate was decompressed and distilled to obtain the colorless liquid compound **8** (7.06 g, 23.7 mmol) in 97% yield. ^{1}H NMR (400 MHz, $CDCl_3$) δ = 9.75 (s, 1H), 7.91 (s, 2H), 3.99-3.96 (t, 2H), 1.81-1.78 (m, 2H), 1.48-1.43 (m, 2H), 1.29-1.24 (m, 4H), 0.84-0.81 (m, 3H). ^{13}C NMR (101 MHz, $CDCl_3$) δ = 188.43, 158.52, 133.96, 133.89, 119.48, 74.13, 31.60, 30.01, 25.48, 22.62, 14.11.

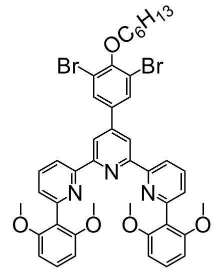

Compound 9. To a flask containing compound **8** (3.64 g, 10 mmol), compound **2** (5.52 g, 22 mmol) and NaOH (1.60 g, 40 mmol), add 80 ml EtOH as solvent and stirred at room temperature for 1 day. Then 30ml $NH_3{\cdot}H_2O$ was added to reflux for one day at 85 °C. After the reaction, it was cooled to room temperature and precipitation would occur. The solid was pumped out and recrystallized with $CHCl_3/CH_3OH$ to give **9** as a white solid (3.44 g, 4.10 mmol) in 41% yield.; ^{1}H NMR (400 MHz, $CDCl_3$, ppm) δ = 8.60-8.58 (d, J = 8 Hz, 2H), 8.58 (s, 2H), 7.94 (s, 2H), 7.94-7.90 (t, 2H), 7.40-7.38 (d, J = 8 Hz, 2H), 7.39-7.35 (t, 2H), 6.73-6.71 (d, J = 8 Hz, 4H), 4.05-4.01 (t, 2H), 3.79 (s, 12H), 1.90-1.84 (m, 2H), 1.56-1.51 (m, 2H), 1.39-1.35 (m, 4H), 0.93-0.90 (m, 3H). ^{13}C NMR (101 MHz, $CDCl_3$, ppm):δ= 158.34, 156.70, 155.79, 153.92, 153.72, 146.53, 137.32, 136.46, 131.47, 129.72, 126.47, 119.59, 119.55, 118.90, 118.71, 104.63, 73.79, 56.16, 31.66, 30.01, 25.54, 22.63, 14.09.

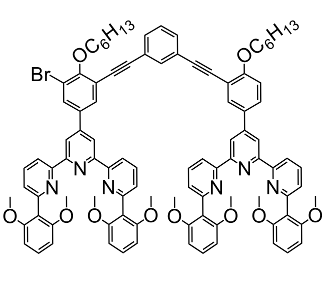

Compound 10. To a flask containing compound **7** (3.22 g, 4.0 mmol) and compound **9** (3.36 g, 4.0 mmol), a mixed solvent (50 mL) of DMSO/Et_3N (3:2, v/v) was added. After $Pd(PPh_3)_4$ (14 mg, 0.12 mmol) and CuI 15 mg was added into the mixture, the system was pumped and backfilled with Ar. Then the mixture was refluxed for 6 h under

Ar. After cooled to 25 °C, the mixture was extracted with CH_2Cl_2 and the combined organic extract was evaporated to dryness under reduced pressure. Then subjected to column chromatography (Al_2O_3, CH_2Cl_2/hexane = 1:1) to give **10** as a white solid (3.20 g, 2.04 mmol) in 49% yield. ^{1}H NMR (400 MHz, $CDCl_3$) δ = 8.65 (s, 2H), 8.63 (s, 2H), 8.61-8.60 (d, J = 4 Hz, 2H), 8.59-8.58 (d, J = 4 Hz, 2H), 7.98-7.96 (d, J = 8 Hz, 2H), 7.92-7.88 (m, 5H), 7.77-7.75 (d, J = 8 Hz, 1H), 7.70 (s, 1H), 7.51-7.49 (d, J = 8 Hz, 1H), 7.47-7.45 (d, J = 8 Hz, 1H), 7.38-7.29 (m, 9H), 6.97-6.94 (d, J = 12 Hz, 1H), 6.69-6.66 (d, J = 8 Hz, 8H), 4.25-4.21 (t, 2H), 4.10-4.07 (t, 2H), 3.76 (s, 12H), 3.75 (s, 12H), 1.90-1.83 (m, 4H), 1.56-1.51 (m, 4H), 1.37-1.29 (m, 8H), 0.87-0.81 (m, 6H). ^{13}C NMR (101 MHz, $CDCl_3$, ppm) δ = 158.34, 158.32, 157.52, 156.68, 156.60, 155.85, 155.75, 153.90, 153.73, 147.24, 147.07, 146.53, 142.45, 139.83, 137.30, 136.42, 135.77, 134.47, 132.43, 132.22, 131.74, 131.65, 131.47, 131.00, 129.73, 128.42, 126.48, 126.41, 124.20, 123.19, 119.56, 119.53, 118.98, 118.90, 118.71, 118.05, 112.90, 112.11, 104.62, 99.98, 99.94, 93.74, 92.57, 86.59, 85.55, 74.47, 73.79, 68.89, 56.16, 56.14, 31.70, 31.66, 31.57, 30.32, 30.01, 29.33, 29.13, 25.83, 25.71, 25.54, 22.63, 14.09, 14.04, 14.01.

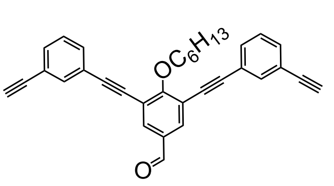

Compound 11. To a flask containing compound **8** (2.55 g, 7.0 mmol) and 1,3-diethynylbenzene (**5**) (2.65 g, 21 mmol), a mixed solvent (50 mL) of THF/Et_3N (3:2, v/v) was added. After $Pd(PPh_3)_4$ (212 mg, 0.18 mmol) and CuI 15 mg was added into the mixture, the system was pumped and backfilled with Ar. Then the mixture was refluxed for 10 h under Ar. After cooled to 25 °C, the mixture was extracted with CH_2Cl_2 and the combined organic extract was evaporated to dryness under reduced pressure. Then subjected to column chromatography (SiO_2, CH_2Cl_2/hexane = 1:4) to give **6** as a white solid (1.69 g, 3.71 mmol) in 53% yield. ^{1}H NMR (400 MHz, $CDCl_3$, ppm) δ = 9.92 (s, 1H), 7.99 (s, 2H), 7.67 (s, 1H), 7.53-7.48 (t, 4H), 7.37-7.33 (t, 2H), 4.52-4.49 (t, 2H), 3.12 (s, 2H), 1.92-1.88 (m, 2H), 1.61-1.57 (m, 2H), 1.36-1.31 (m, 4H), 0.87-0.83 (m, 3H). ^{13}C NMR (101 MHz, $CDCl_3$, ppm) δ = 188.64, 134.06, 133.98, 131.32, 130.76, 127.57, 122.09, 121.66, 116.70, 92.86, 84.10, 81.54, 77.05, 73.83, 30.70, 29.54, 24.90, 21.63, 13.02.

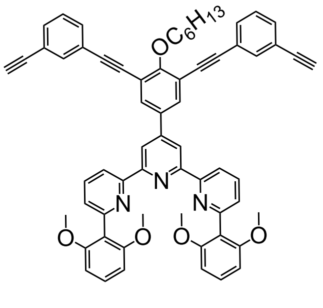

Compound 12. To a flask containing compound **11** (2.28 g, 5.0 mmol), compound **2** (2.76 g, 11 mmol) and NaOH (0.80 g, 20 mmol), add 50 ml EtOH as solvent and stirred at room temperature for 1 day. Then 20 ml $NH_3·H_2O$ was added to reflux for one day at 85 °C. After the reaction, it was cooled to room temperature, the mixture was extracted

with CH_2Cl_2 and the combined organic extract was evaporated to dryness under reduced pressure. Then subjected to column chromatography (Al_2O_3, CH_2Cl_2/hexane = 1:2) to give **12** as a white solid (1.67 g, 1.80 mmol) in 36% yield. ^{1}H NMR (400 MHz, $CDCl_3$, ppm) δ = 8.66 (s, 2H), 8.62-8.60 (d, J = 8 Hz, 2H), 7.93 (s, 2H), 7.91-7.81 (m, 2H), 7.66 (s, 2H), 7.51-7.49 (d, J = 8 Hz, 2H), 7.47-7.45 (d, J = 8 Hz, 2H), 7.39-7.32 (m, 6H), 6.72-6.70 (d, J = 8 Hz, 4H), 4.38-4.35 (t, 2H), 3.78 (s, 12H), 3.11 (s, 2H), 1.91-1.86 (m, 2H), 1.63-1.56 (m, 2H), 1.35-1.29 (m, 4H), 0.86-0.83 (m, 3H). ^{13}C NMR (101 MHz, $CDCl_3$, ppm) δ = 199.99, 160.51, 157.37, 154.95, 152.85, 146.73, 135.21, 134.00, 131.75, 130.94, 130.78, 128.98, 128.70, 127.45, 125.35, 122.60, 121.53, 118.58, 118.03, 116.59, 103.69, 91.86, 85.20, 81.72, 76.79, 73.76, 55.17, 30.78, 29.56, 25.03, 21.64, 13.03.

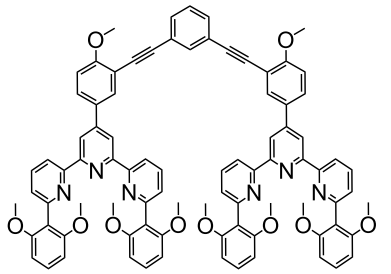

Compound L1. To a flask containing compound **3** (3.18 g, 5.0 mmol) and 1,3-diiodobenzene (750 mg, 2.27 mmol), a mixed solvent (50 mL) of THF/Et_3N (3:2, v/v) was added. After $Pd(PPh_3)_4$ (212 mg, 0.18 mmol) and CuI 15 mg was added into the mixture, the system was pumped and backfilled with Ar. Then the mixture was refluxed for 10 h under Ar. After cooled to 25 °C, the mixture was extracted with CH_2Cl_2 and the combined organic extract was evaporated to dryness under reduced pressure. Then subjected to column chromatography (Al_2O_3, CH_2Cl_2/hexane = 1:1) to give compound **L1** as a white solid (2.46 g, 1.66 mmol) in 73% yield. ^{1}H NMR (400 MHz, $CDCl_3$, ppm) δ = 8.66 (s, 4H), 8.60-8.59 (d, J = 4 Hz, 4H), 7.99 (s, 2H), 7.92-7.88(s, 4H), 7.80 (s, 1H), 7.77-7.76 (d, J = 4 Hz, 2H), 7.51-7.49 (d, J = 8 Hz, 2H), 7.37-7.35 (d, J = 8 Hz, 4H), 7.33-7.29 (m, 5H), 6.98-6.96 (d, J = 8 Hz, 2H), 6.68-6.66 (d, J = 8 Hz, 8H), 3.94 (s, 6H), 3.75 (s, 24H). ^{13}C NMR (101 MHz, $CDCl_3$, ppm) δ = 160.38, 158.35, 156.45, 156.22, 153.88, 148.42, 136.43, 134.88, 132.59, 131.42, 131.29, 129.68, 129.02, 128.33, 126.24, 123.71, 119.72, 119.56, 118.81, 112.63, 110.91, 104.60, 92.92, 86.10, 56.18, 56.04.

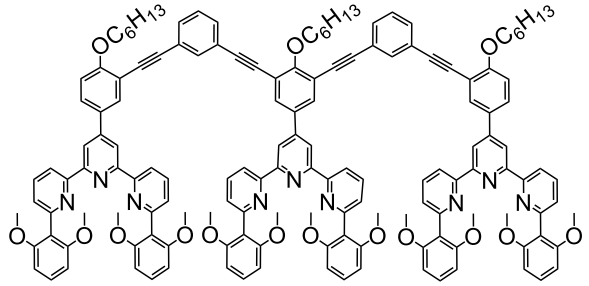

Compound L2. To a flask containing compound **7** (2.01 g, 2.50 mmol) and compound **9** (1.0 g, 1.19 mmol), a mixed solvent (50 mL) of DMSO/Et_3N (3:2, v/v) was added. After $Pd(PPh_3)_4$ (0.14 g, 0.12 mmol) and CuI 15 mg was added into the mixture, the system was pumped and backfilled with Ar. Then the mixture was refluxed for 10 h under Ar. After cooled to 25 °C, the mixture was extracted with CH_2Cl_2 and the combined organic extract was

evaporated to dryness under reduced pressure. Then subjected to column chromatography (Al_2O_3, CH_2Cl_2/hexane = 1:1) to give compound **L2** as a white solid (1.39 g, 2.04 mmol) in 51% yield. ^{1}H NMR (400 MHz, $CDCl_3$, ppm) δ = 8.67 (s, 2H), 8.65 (s, 4H), 8.60-8.58 (d, *J* = 8 Hz, 6H), 7.97-7.96 (d, *J* = 4 Hz, 2H), 7.93 (s, 2H), 7.91-7.88 (t, 6H), 7.77-7.75 (d, *J* = 8 Hz, 2H), 7.71 (s, 2H), 7.51-7.47 (t, 4H), 7.36-7.24 (m, 16H), 6.96-6.94 (d, *J* = 8 Hz, 2H), 6.68-6.66 (d, *J* = 8 Hz, 8H), 6.65-6.63 (d, *J* = 8 Hz, 4H), 4.41-4.38 (t, 2H), 4.10-4.07 (t, 4H), 3.74 (s, 24H), 3.73 (s, 12H), 1.89-1.83 (m, 6H), 1.55-1.52 (m, 6H), 1.37-1.22 (m, 13H), 0.87-0.83 (t, 6H), 0.77-0.73 (t, 3H). ^{13}C NMR (101 MHz, $CDCl_3$, ppm) δ = 161.55, 160.20, 158.34, 158.30, 156.54, 155.93, 153.92, 153.79, 148.63, 147.91, 136.54, 136.47, 136.41, 134.47, 134.42, 132.70, 132.21, 131.49, 131.17, 131.04, 129.71, 129.05, 128.38, 126.36, 126.28, 124.13, 123.48, 119.57, 119.50, 119.06, 118.84, 117.83, 112.94, 112.12, 104.62, 93.21, 92.67, 86.50, 85.96, 74.73, 68.89, 56.16, 56.13, 31.78, 31.56, 30.58, 29.13, 26.04, 25.71, 22.66, 22.63, 14.01.

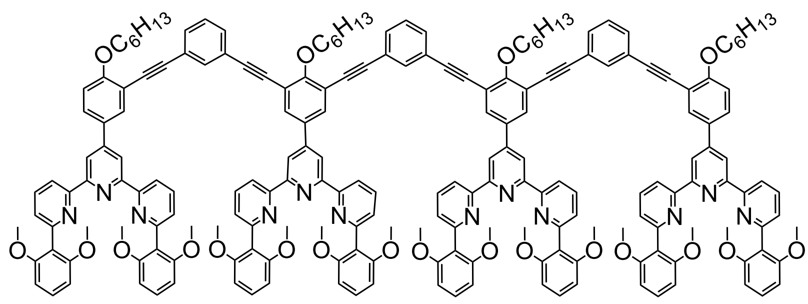

Compound L3. To a flask containing compound **10** (3.13 g, 2.0 mmol) and compound **5** (84 mg, 0.67 mmol), a mixed solvent (45 mL) of DMSO/Et_3N (3:2, v/v) was added. After $Pd(PPh_3)_4$ (0.14 g, 0.12 mmol) and CuI 15 mg was added into the mixture, the system was pumped and backfilled with Ar. Then the mixture was refluxed for 10 h under Ar. After cooled to 25 °C, the mixture was extracted with CH_2Cl_2 and the combined organic extract was evaporated to dryness under reduced pressure. Then subjected to column chromatography (Al_2O_3, CH_2Cl_2/hexane = 1:1) to give compound **L3** as a white solid (664 mg, 0.21 mmol) in 32% yield. ^{1}H NMR (400 MHz, $CDCl_3$) δ 8.67 (s, 4H), 8.65 (s, 4H), 8.60 (d, 4H), 8.58 (d, 4H), 7.97-7.96 (d, *J* = 8 Hz, 2H), 7.93 (s, 4H), 7.91-7.87 (t, 8H), 7.76-7.73 (m, 3H), 7.71 (s, 2H), 7.52-7.47 (m, 6H), 7.35-7.23 (m, 19H), 6.96-6.94 (d, *J* = 8 Hz, 2H), 6.68-6.65 (d, *J* = 8 Hz, 8H), 6.64-6.62 (d, *J* = 8 Hz, 8H), 4.41-4.38 (t, 4H), 4.09-4.06 (t, 4H), 3.74 (s, 24H), 3.72 (s, 24H), 1.89-1.84 (m, 8H), 1.55-1.50 (m, 8H), 1.33-1.24 (m, 16H), 0.86-0.83 (t, 6H), 0.77-0.74 (t, 6H). ^{13}C NMR (101 MHz, $CDCl_3$, ppm) δ = 161.55, 160.17, 158.33, 158.28, 156.58, 156.40, 156.19, 155.95, 153.96, 153.87, 148.55, 147.90, 136.36, 134.49, 134.46, 132.79, 132.68, 132.21, 131.50, 131.41, 131.24, 131.04, 129.92, 129.89, 129.70, 129.64, 129.00, 128.51, 128.39, 126.34, 126.20, 124.14, 123.66, 123.46, 119.77, 119.61, 119.51, 119.49, 119.02, 118.77, 117.84, 117.77, 112.93, 112.11, 104.62, 104.59, 93.24, 93.01, 92.65, 86.53, 86.18, 85.93, 77.23, 74.74, 68.88, 56.14, 56.10, 31.78, 31.56, 30.58, 29.71, 29.32, 29.13, 26.04, 25.71, 22.66, 22.63, 14.01, 1.03.

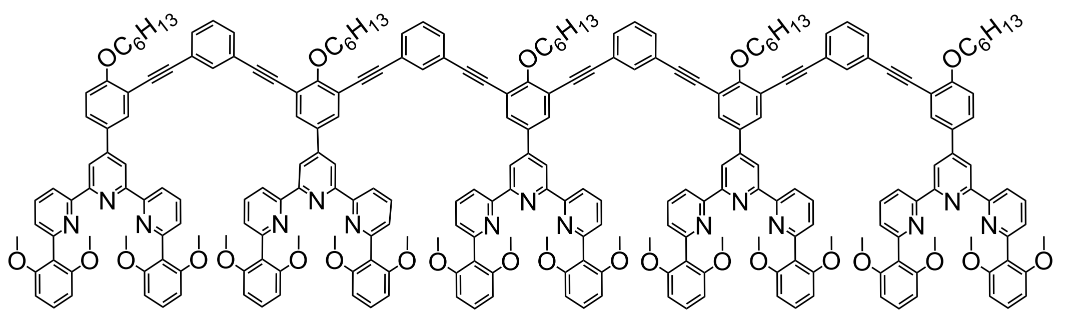

Compound L4. To a flask containing compound **10** (1.57 g, 1.0 mmol) and compound **12** (372 mg, 0.40 mmol), a mixed solvent (30 mL) of DMSO/Et_3N (3:2, v/v) was added. After $Pd(PPh_3)_4$ (0.14 g, 0.12 mmol) and CuI 15 mg was added into the mixture, the system was pumped and backfilled with Ar. Then the mixture was refluxed for 10 h under Ar. After cooled to 25 °C, the mixture was extracted with CH_2Cl_2 and the combined organic extract was evaporated to dryness under reduced pressure. Then subjected to column chromatography (Al_2O_3, CH_2Cl_2/hexane = 3:1) to give compound **L2** as a white solid (468 mg, 0.12 mmol) in 30% yield. ^{1}H NMR (400 MHz, $CDCl_3$) δ 8.67 (s, 8H), 8.66 (s, 2H), 8.60-8.58 (d, *J* = 8 Hz, 10H), 7.97-7.96 (d, *J* = 4 Hz, 2H), 7.93 (s, 6H), 7.91-7.87 (m, 10H), 7.78-7.76 (d, *J* = 8 Hz, 2H), 7.73 (s, 2H), 7.71 (s, 2H), 7.52-7.47 (m, 10H), 7.37-7.23 (m, 24H), 6.96-6.94 (d, *J* = 8 Hz, 2H), 6.67-6.65 (d, 4H), 6.64-6.62 (d, 6H), 4.41-4.38 (t, 6H), 4.09-4.06 (t, 4H), 3.74 (s, 24H), 3.72 (s, 24H), 3.71 (s, 12H), 1.89-1.84 (m, 10H), 1.59-1.51 (m, 10H), 1.33-1.24 (m, 20H), 0.86-0.83 (t, 6H), 0.77-0.74 (t, 9H). ^{13}C NMR (101 MHz, $CDCl_3$, ppm) δ = 161.55, 160.20, 158.33, 158.28, 156.55, 155.92, 153.95, 153.82, 148.63, 147.91, 136.40, 134.46, 132.79, 132.68, 132.21, 131.50, 131.42, 131.17, 131.04, 129.73, 129.05, 128.52, 128.39, 126.37, 126.27, 124.14, 123.66, 123.64, 123.45, 119.57, 119.51, 119.05, 118.83, 117.84, 117.78, 112.93, 112.13, 104.62, 93.25, 93.06, 93.01, 92.65, 86.52, 86.19, 86.14, 85.93, 77.23, 74.74, 68.89, 56.15, 56.11, 40.98, 31.78, 31.56, 30.58, 29.78, 29.33, 29.13, 27.22, 26.04, 25.71, 22.66, 22.63, 14.03, 14.01.

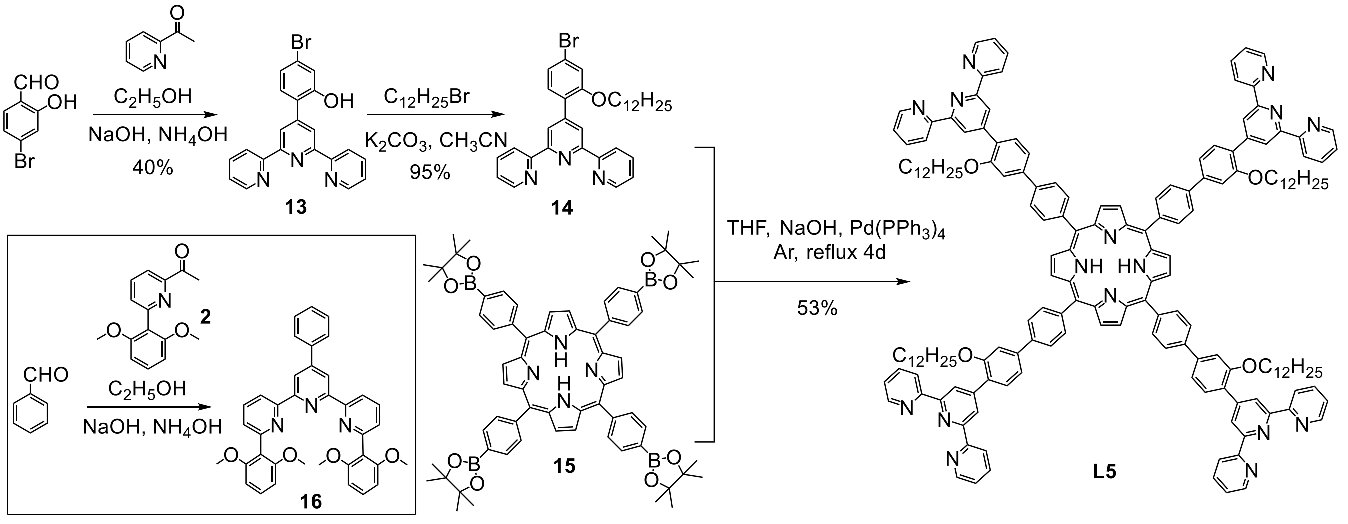

Scheme S2. Synthesis of ligands **L5**.

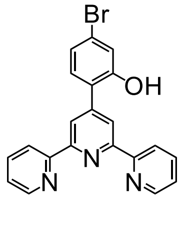

Compound 13. 4-bromo-2-hydroxybenzaldehyde (4.02 g, 20 mmol), 2-acetylpyridine (5.08 g, 42 mmol) and NaOH (3.2 g, 80 mmol) were mixed in 80 ml EtOH and stirred at room temperature for 1 day. Then 40 ml $NH_3 \cdot H_2O$ was added to reflux for one day at 85 °C. After the reaction, it was cooled to room temperature and precipitation would occur. The yellow solid was pumped out and wash with a small amount of CH_3OH to give **13** as a yellow solid (3.23 g, 8.0 mmol) in 40% yield. ^{1}H NMR (400 MHz, DMSO) δ = 8.69-8.67 (d, *J* = 8 Hz, 2H), 8.67 (s, 2H), 8.63-8.61 (d, *J* = 8 Hz, 2H), 8.02-7.98 (t, 2H), 7.49-7.45 (m, 2H), 7.39-7.37 (d, *J* = 8 Hz, 2H), 7.06 (s, 1H), 6.93-6.90 (d, 1H). ^{13}C NMR (101 MHz, DMSO) δ = 156.72, 154.56, 151.20, 149.59, 146.43, 141.10, 140.97, 137.55, 130.42, 124.21, 123.94, 123.57, 121.10, 119.82.

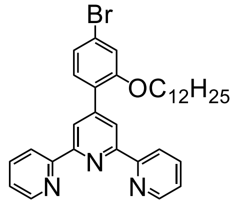

Compound 14. To a flask containing compound **13** (2.02 g, 5.0 mmol), 1-bromododecane (1.88 g, 7.5 mmol) and K_2CO_3 (1.73 g, 12.5 mmol), add 60 ml DMF as solvent. The system was pumped and backfilled with Ar. Then the mixture was refluxed for 1 days under Ar. After cooled to 25 °C, the mixture was extracted with CH_2Cl_2 and the combined organic extract was evaporated to dryness under reduced pressure. Then subjected to column chromatography (Al_2O_3, CH_2Cl_2/hexane = 1:10) to give compound **14** as a white solid (2.72 g, 23.7 mmol) in 95% yield. ^{1}H NMR (400 MHz, $CDCl_3$, ppm) δ = 8.73-8.72 (d, *J* = 4 Hz, 2H), 8.70-8.69 (d, *J* = 4 Hz, 4H), 7.91-7.88 (t, 2H), 7.47-7.45 (d, *J* = 8 Hz, 1H), 7.37-7.35 (t, *J* = 8 Hz, 2H), 7.23-7.21 (d, *J* = 8 Hz, 1H), 7.16 (s, 1H), 4.03-4.01 (t, *J* = 8 Hz, 2H), 1.76-1.71 (m, 2H), 1.43-1.36 (m, 2H), 1.28-1.09 (m, 16H), 0.91-0.88 (t, 3H). ^{13}C NMR (101 MHz, $CDCl_3$, ppm) δ= 156.96, 156.20, 155.03, 148.97, 147.51, 137.00, 131.63, 127.25, 123.81, 123.72, 123.36, 121.72, 121.34, 115.72, 68.91, 31.93, 29.63, 29.62, 29.58, 29.40, 29.38, 29.37, 29.05, 26.12, 22.71, 14.16.

Compound 15 were synthesized according to the reported procedures. [S1]

Compound 16 were synthesized according to the reported procedures. [S2]

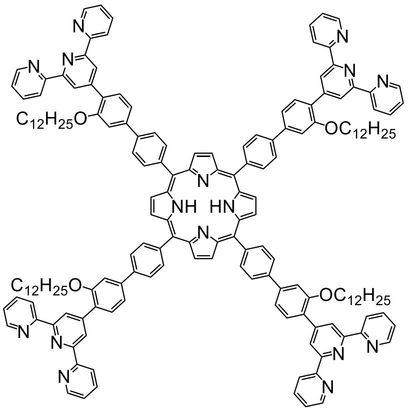

Compound L5. To a flask containing compound **15** (559 mg, 0.5 mmol), compound **14** (1.72 mg, 3.0 mmol) and K_2CO_3 (690 mg, 12.5 mmol) with 2 ml H_2O, then add 30 ml THF as solvent. After $Pd(PPh_3)_4$ (140 mg, 0.12 mmol) was added, the system was pumped and backfilled with Ar. Then the system was refluxed for 4 days under Ar. After cooled to 25 °C, the mixture was extracted with $CHCl_3$ and the combined organic extract was evaporated to dryness in vacuo to give a reside that was washed with MeOH, then subjected to column chromatography (Al_2O_3, CH_2Cl_2/MeOH = 100:1) and then recrystallized from a mixture of $CHCl_3$/MeOH to give **L5**, as a purple powder: (684 mg, 0.26 μmol), in 53% yield.. ^1H NMR (400 MHz, $CDCl_3$) δ = 9.06 (s, 8H), 8.90 (s, 8H), 8.78 (d, *J* = 4.5 Hz, 8H), 8.75 (d, *J* = 8.0 Hz, 8H), 8.39 (d, *J* = 7.9 Hz, 8H), 8.10 (d, *J* = 8.0 Hz, 8H), 7.92 (t, *J* = 8.6 Hz, 8H), 7.86 (d, *J* = 7.7 Hz, 4H), 7.65 (d, *J* = 8.7 Hz, 4H), 7.60 (s, 4H), 7.38 (m, 8H), 4.29 (t, *J* = 6.2 Hz, 8H), 1.88 (m, 8H), 1.53 (m, 8H), 1.25 (m, 64H), 0.90 (t, *J* = 7.0 Hz, 12H); ^{13}C NMR (125 MHz, $CDCl_3$) δ = 157.07, 156.66, 155.23, 149.18, 148.20, 142.68, 141.60, 140.13, 136.79, 135.19, 131.25, 127.65, 125.52, 123.61, 121.92, 121.29, 119.96, 119.85, 111.39, 68.89, 31.95, 29.69, 29.66, 29.53, 29.49, 29.39, 26.32, 22.72, 14.16.

3. NMR and MS spectra of compounds

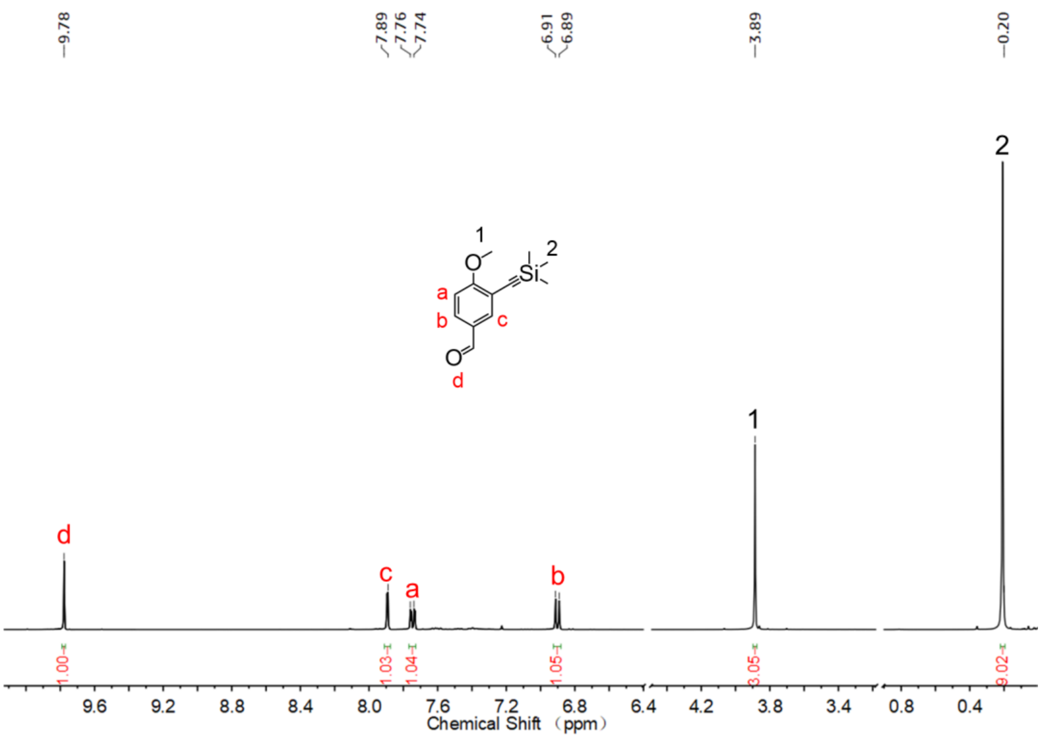

Figure S1. ^{1}H NMR spectrum of compound **1** in $CDCl_3$.

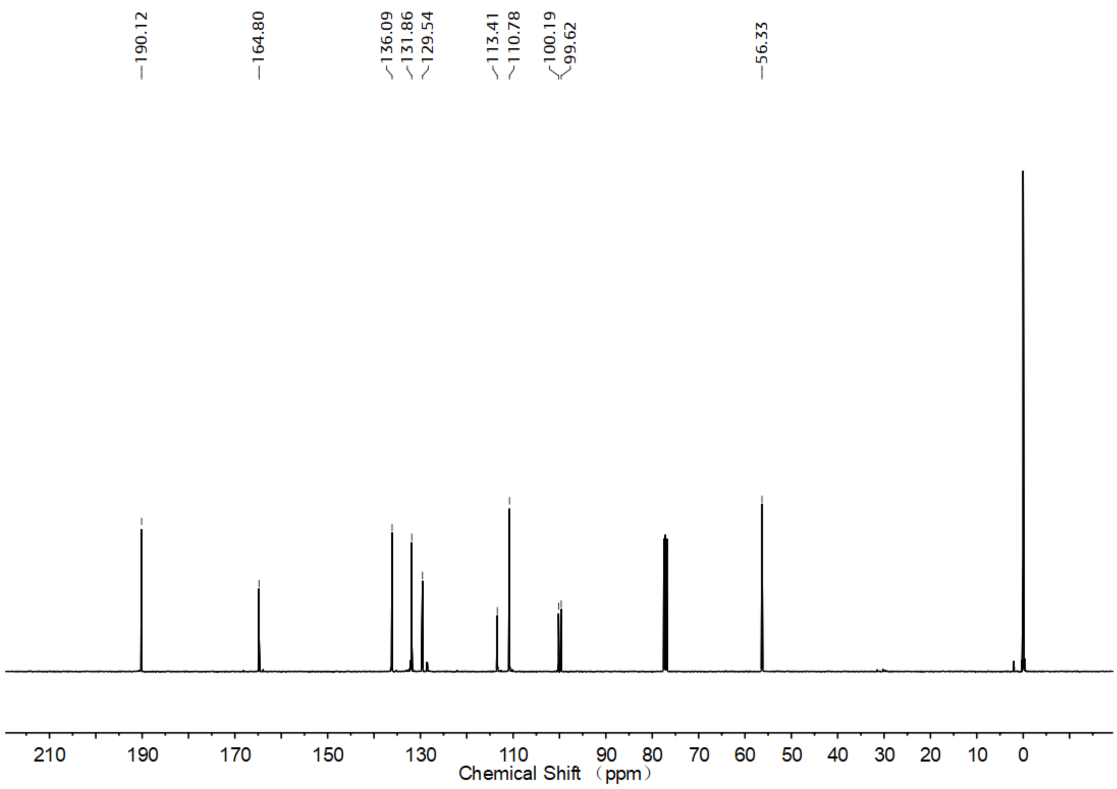

Figure S2. ^{13}C NMR spectrum of compound **1** in $CDCl_3$.

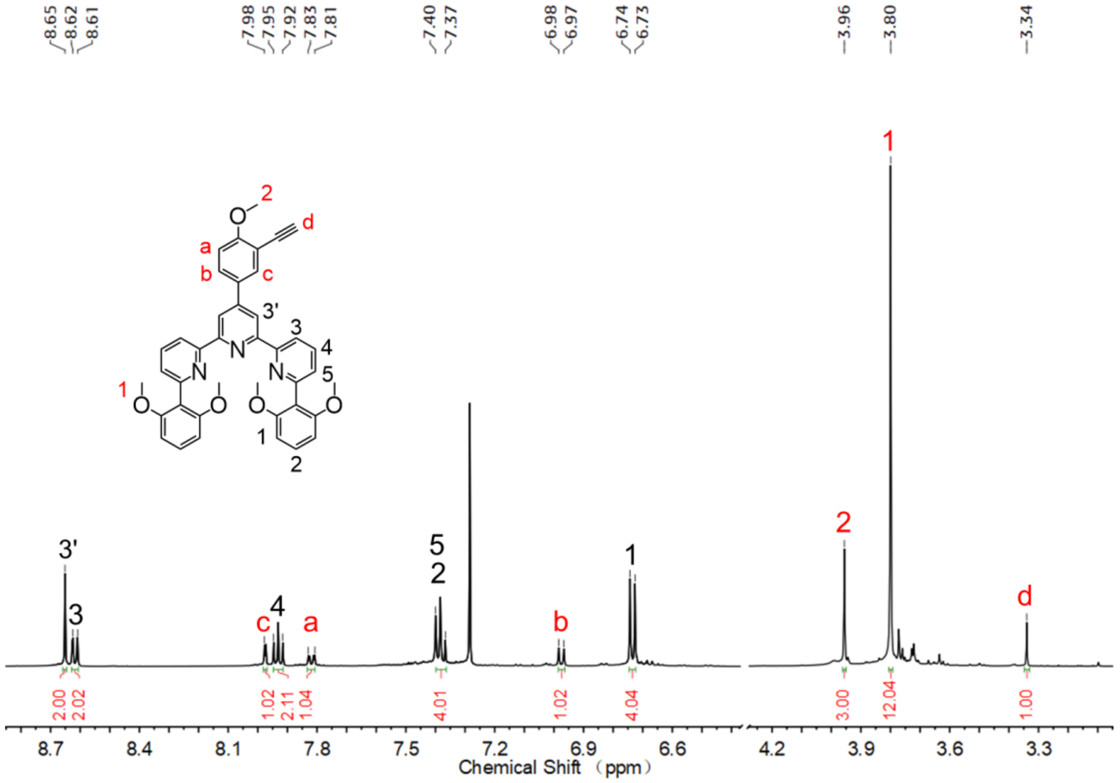

Figure S3. ^{1}H NMR spectrum of compound **3** in $CDCl_3$.

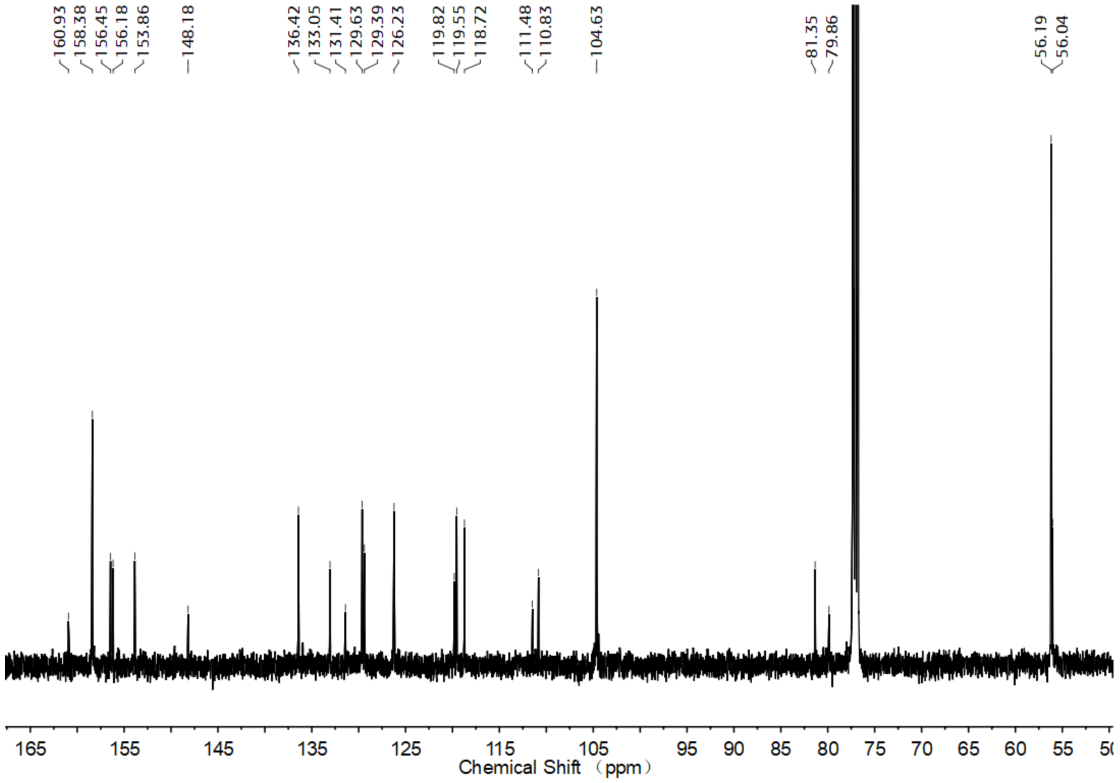

Figure S4. ^{13}C NMR spectrum of compound **3** in $CDCl_3$.

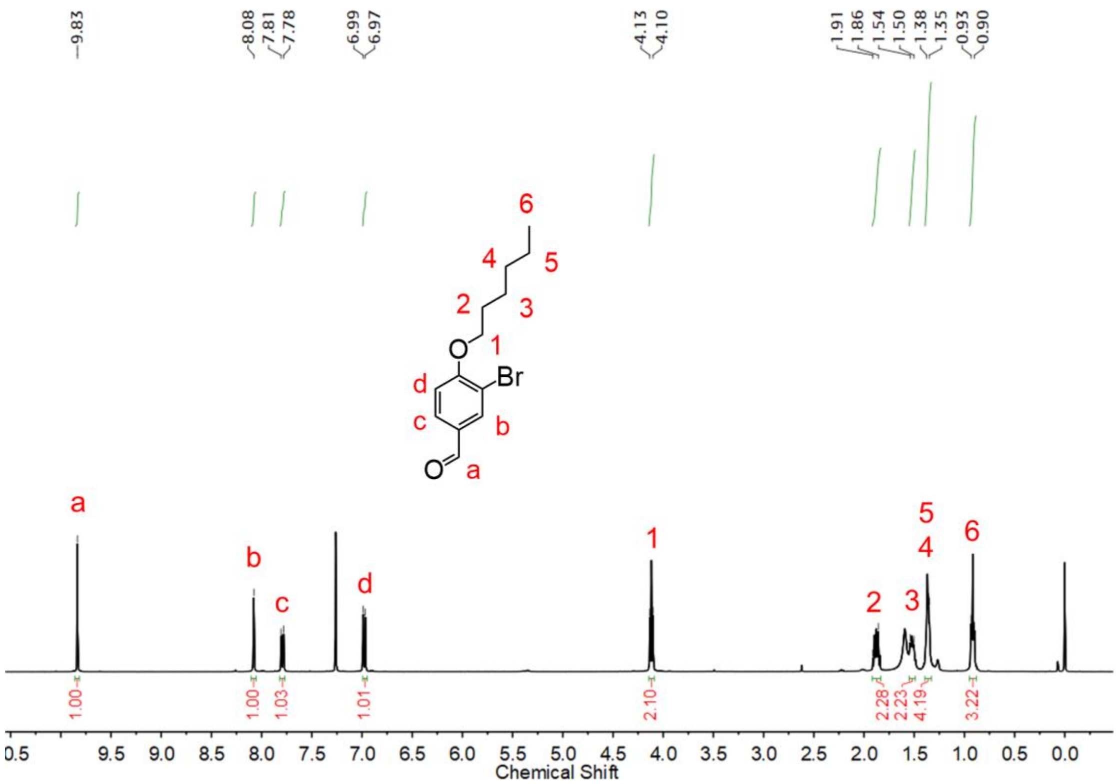

Figure S5. ^{1}H NMR spectrum of compound **4** in $CDCl_3$.

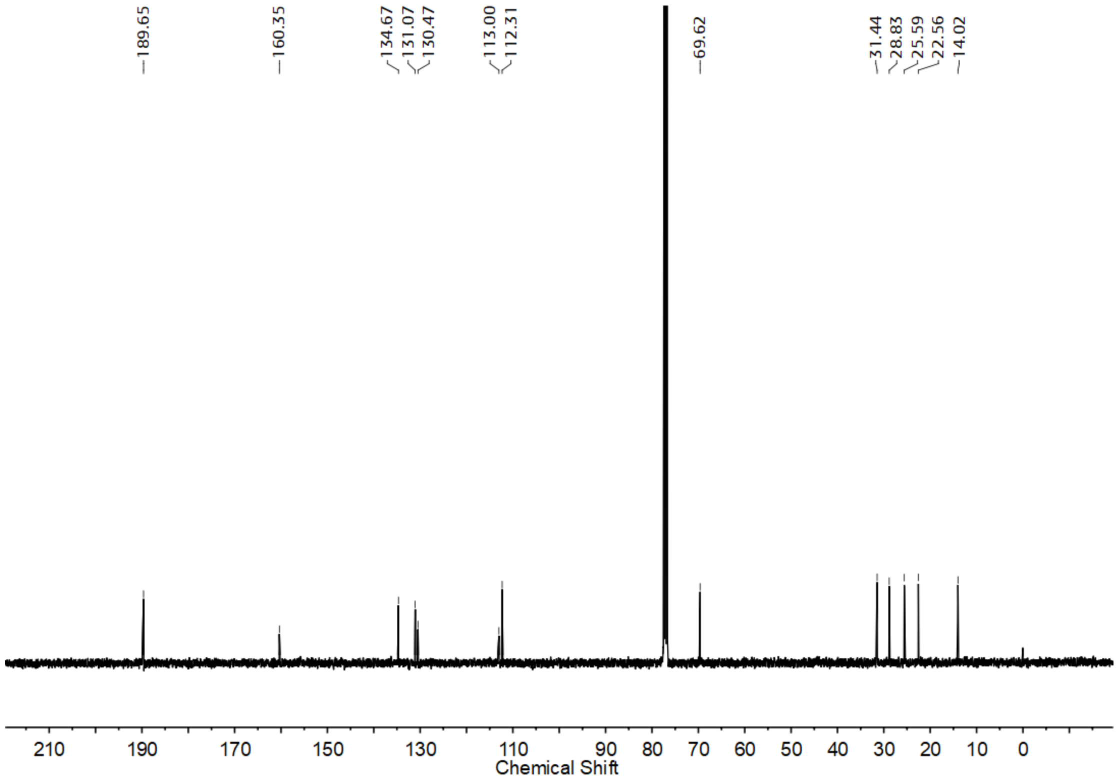

Figure S6. ^{13}C NMR spectrum of compound **4** in $CDCl_3$.

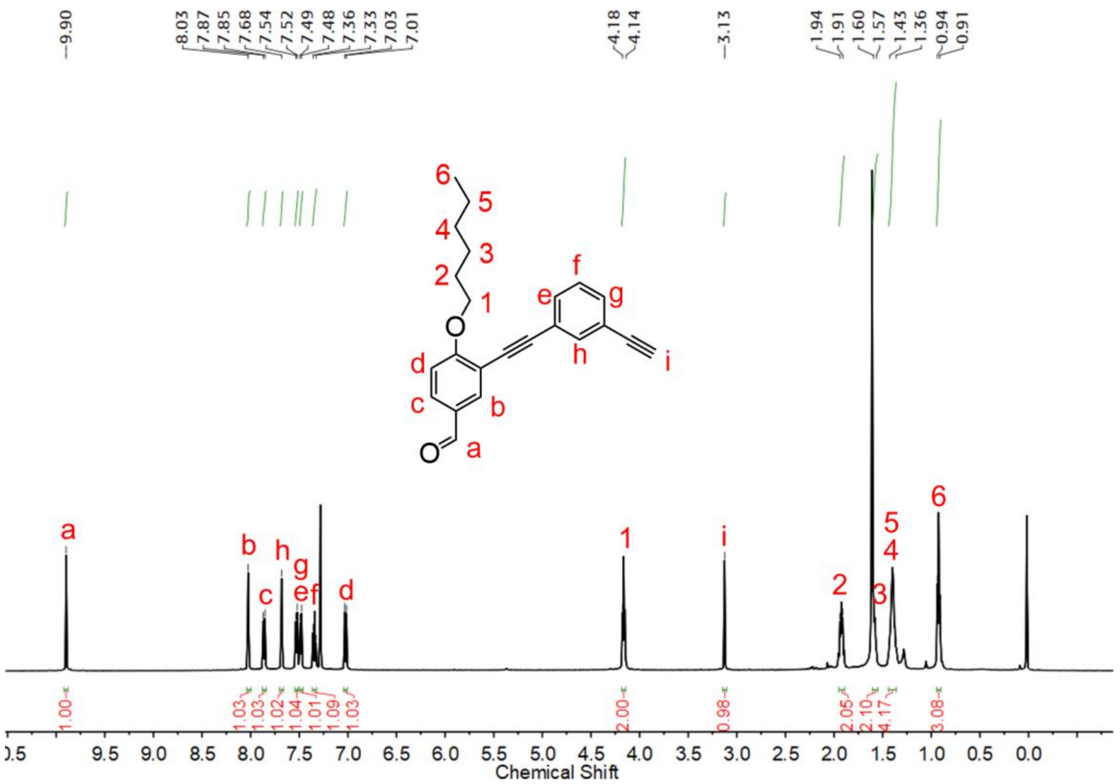

Figure S7. ^{1}H NMR spectrum of compound **6** in $CDCl_3$.

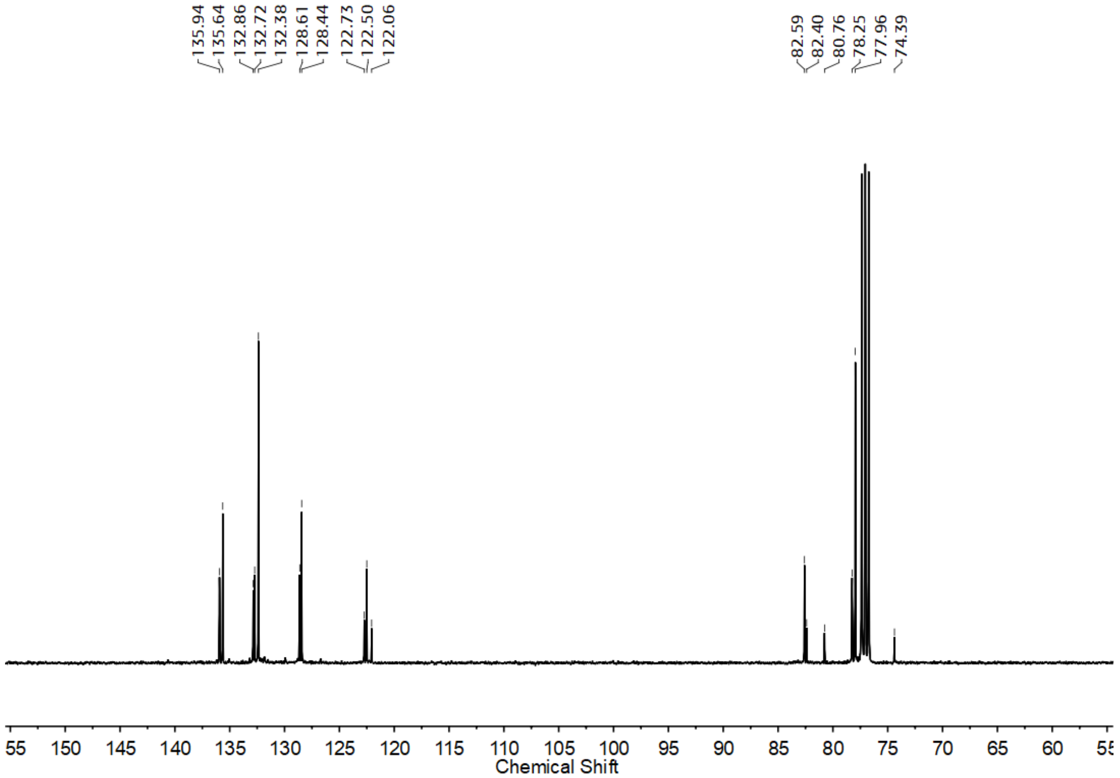

Figure S8. ^{13}C NMR spectrum of compound **6** in $CDCl_3$.

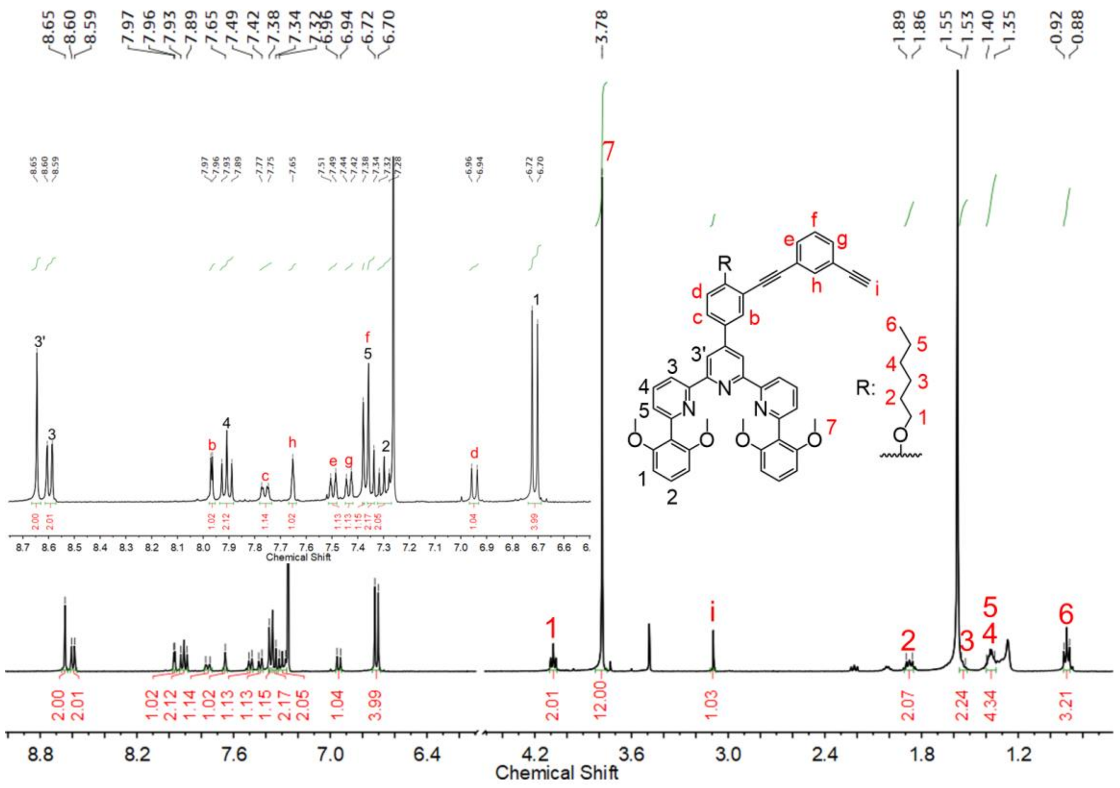

Figure S9. ^{1}H NMR spectrum of compound **7** in $CDCl_3$.

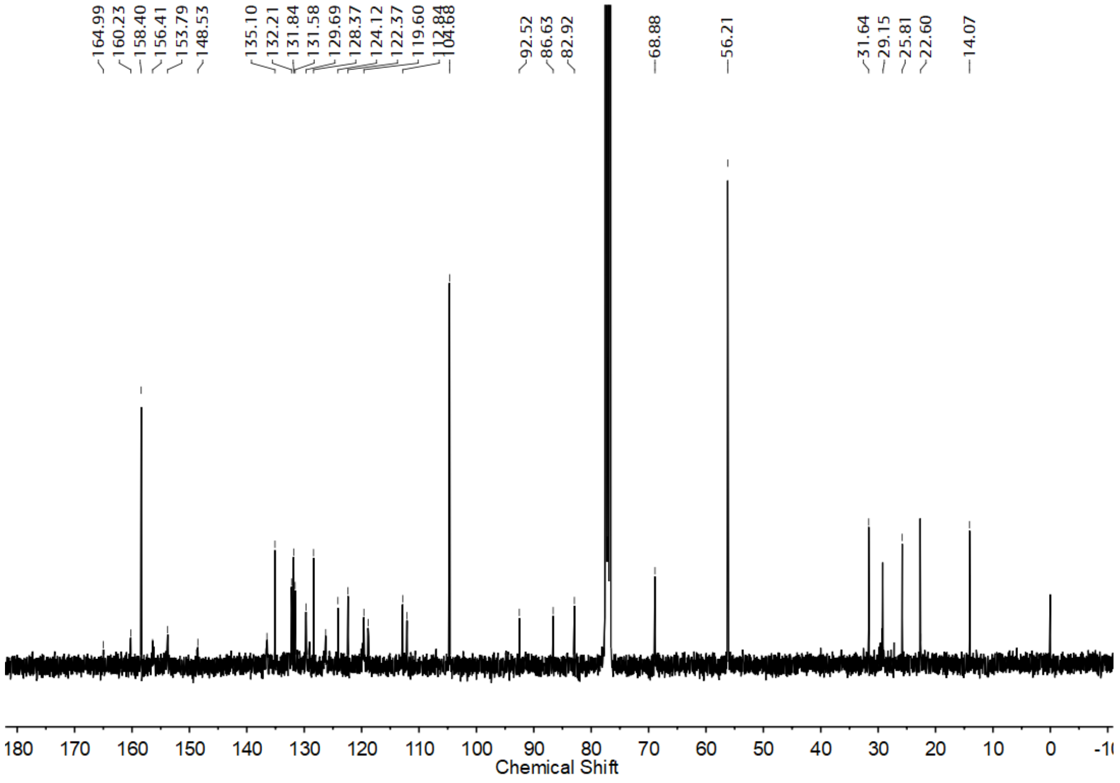

Figure S10. ^{13}C NMR spectrum of compound **7** in $CDCl_3$.

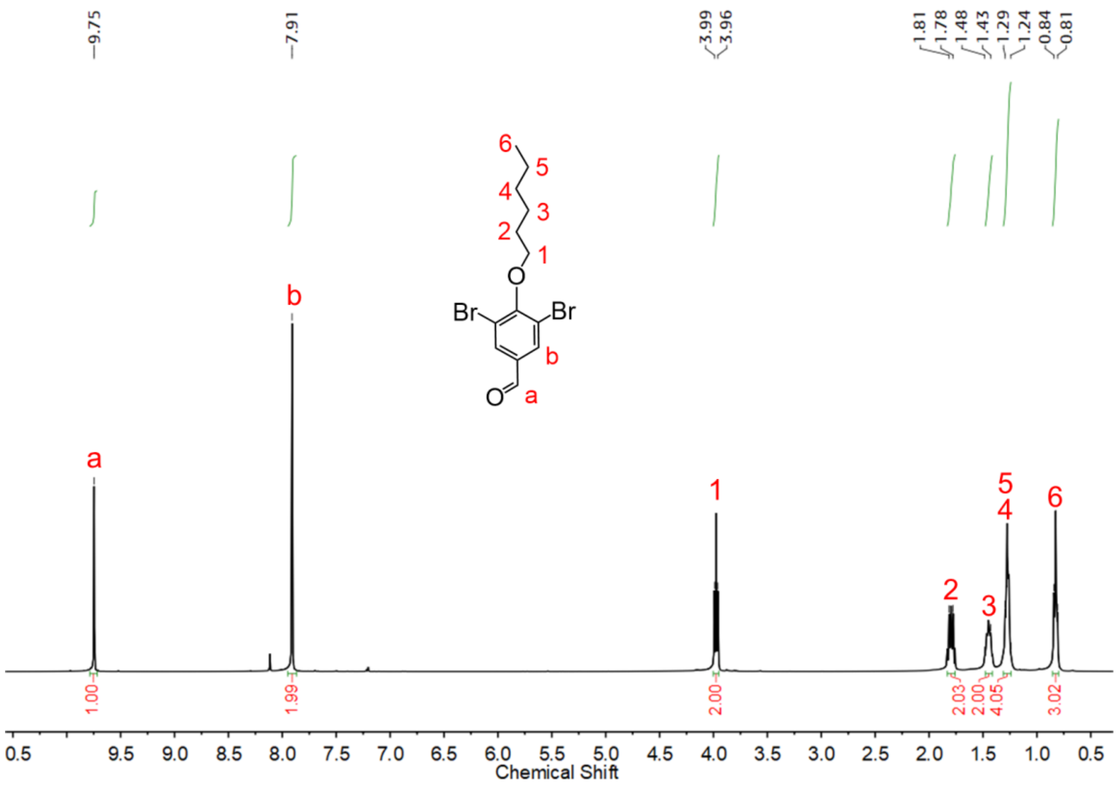

Figure S11. ^{1}H NMR spectrum of compound **8** in $CDCl_3$.

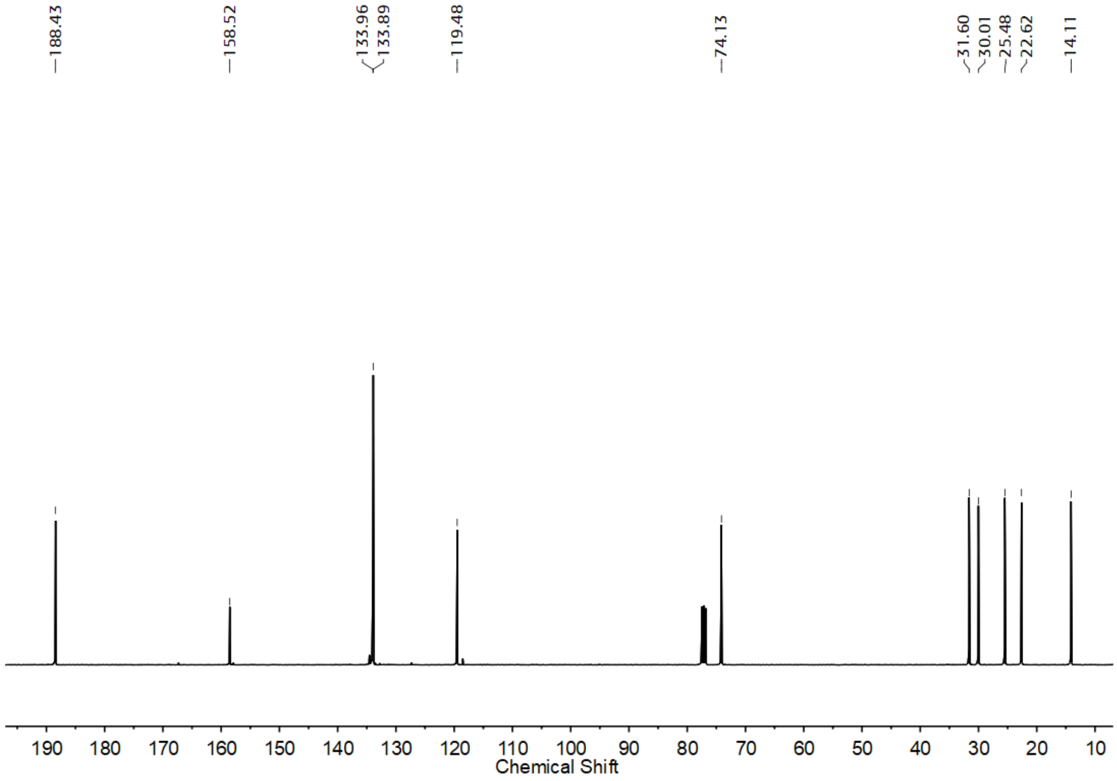

Figure S12. ^{13}C NMR spectrum of compound **8** in $CDCl_3$.

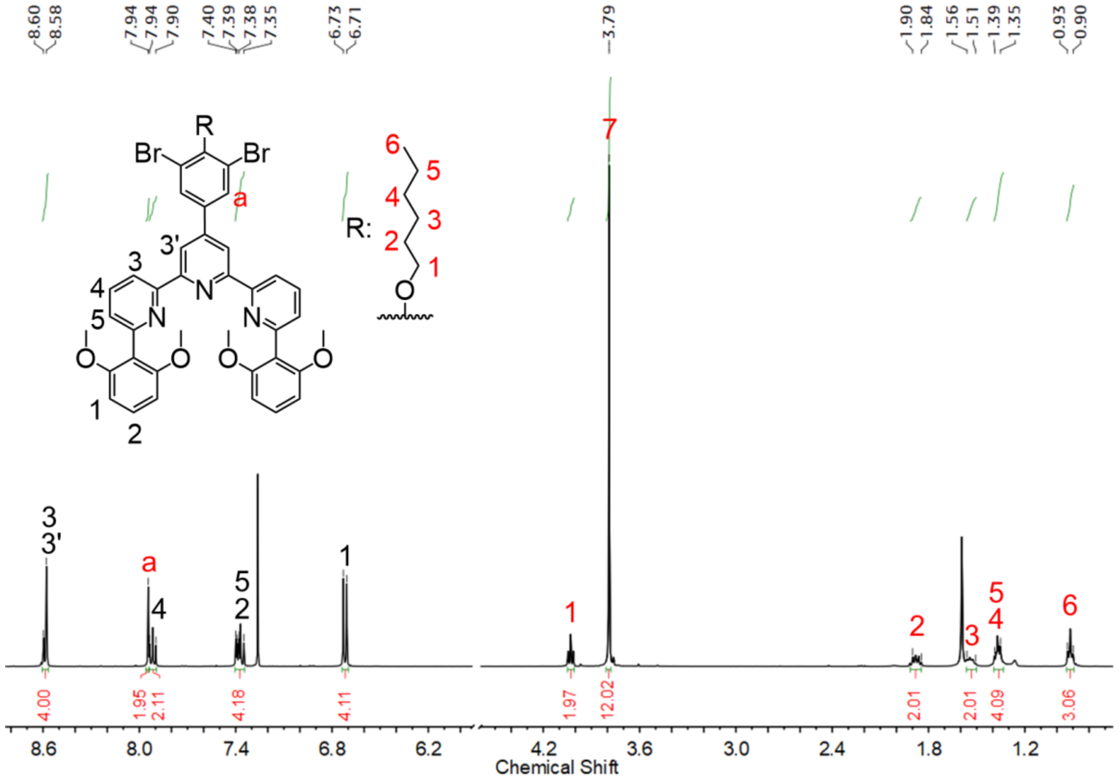

Figure S13. ^{1}H NMR spectrum of compound **9** in $CDCl_3$.

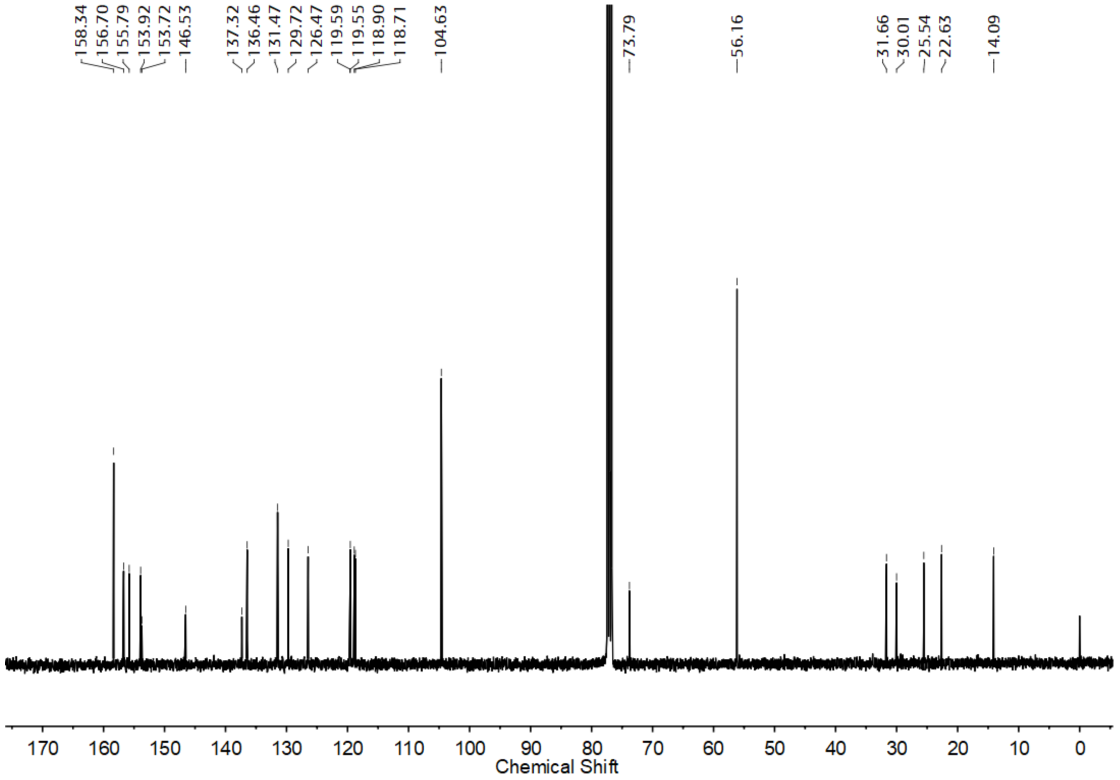

Figure S14. ^{13}C NMR spectrum of compound **9** in $CDCl_3$.

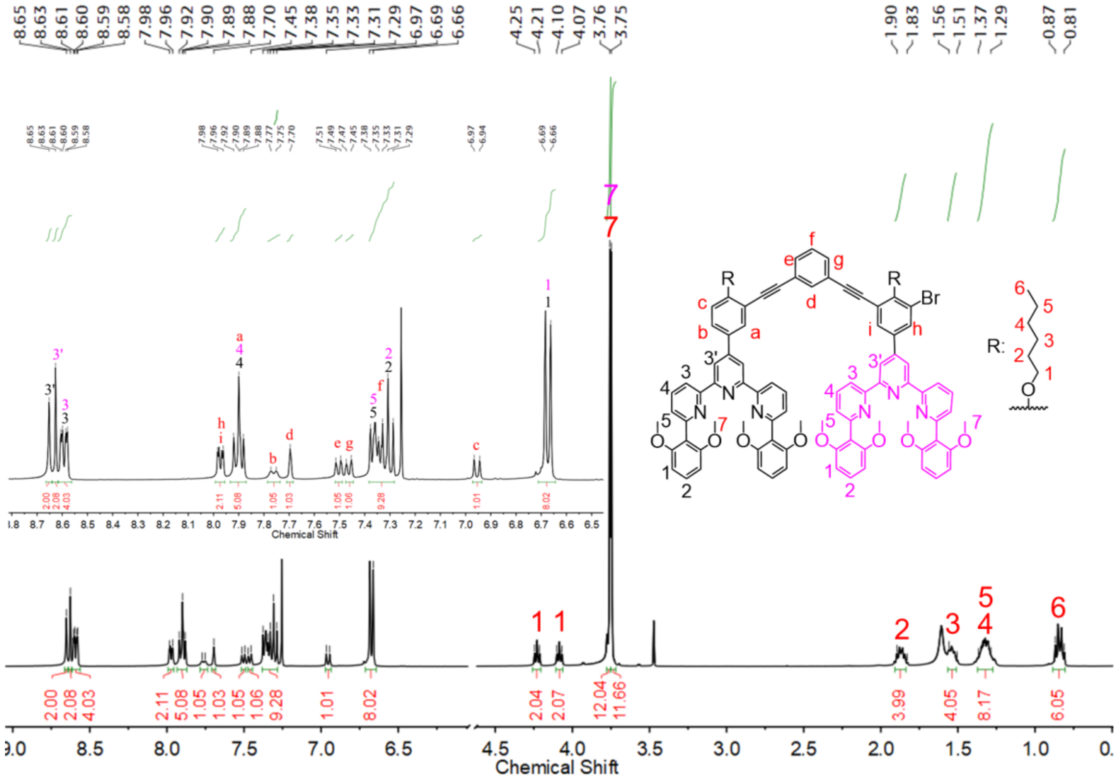

Figure S15. ^{1}H NMR spectrum of compound **10** in $CDCl_3$.

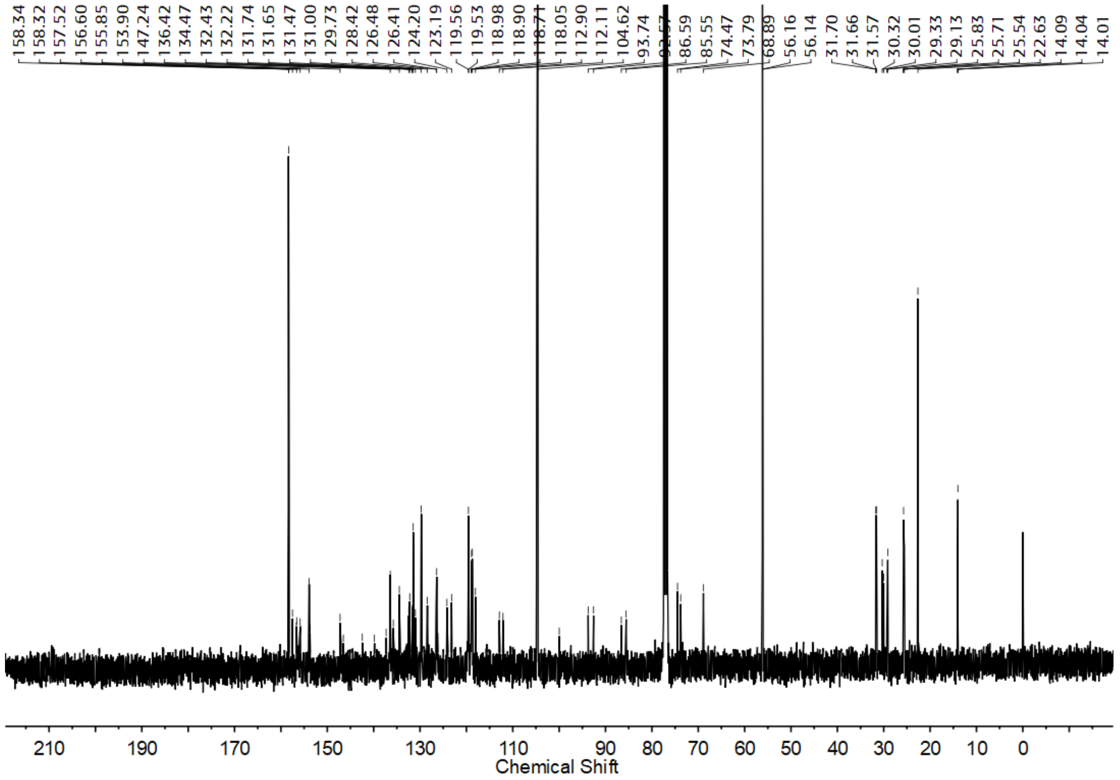

Figure S16. ^{13}C NMR spectrum of compound **10** in $CDCl_3$.

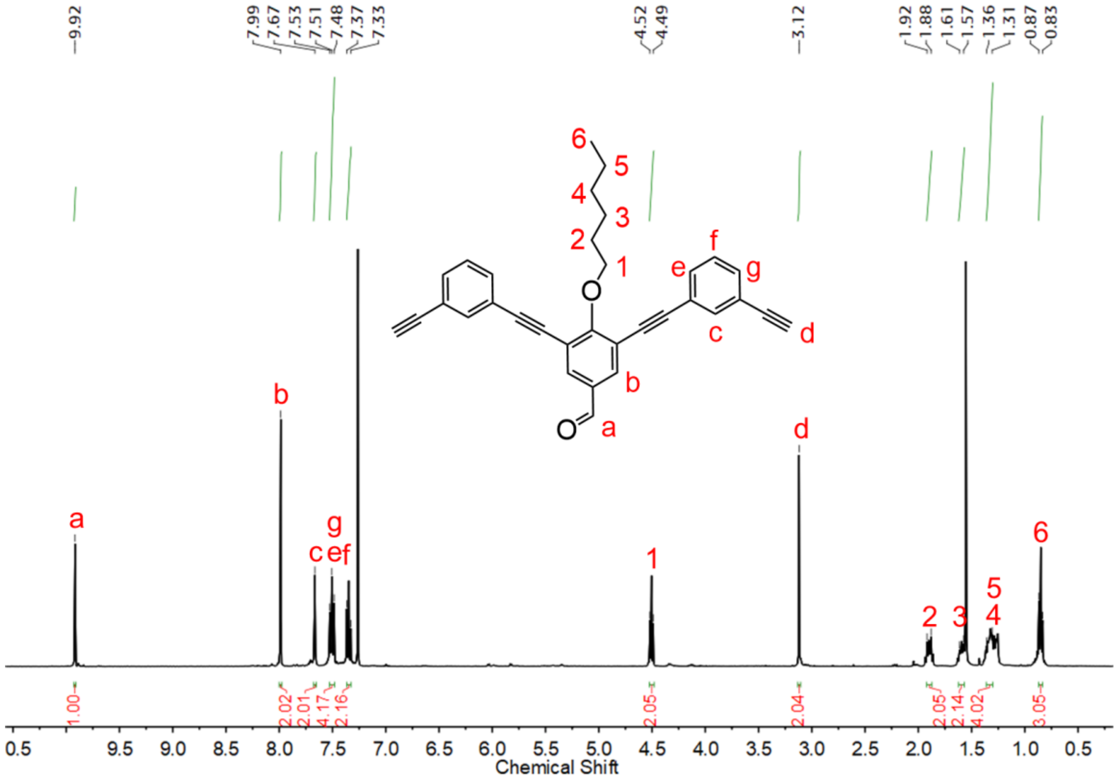

Figure S17. ^{1}H NMR spectrum of compound **11** in $CDCl_3$.

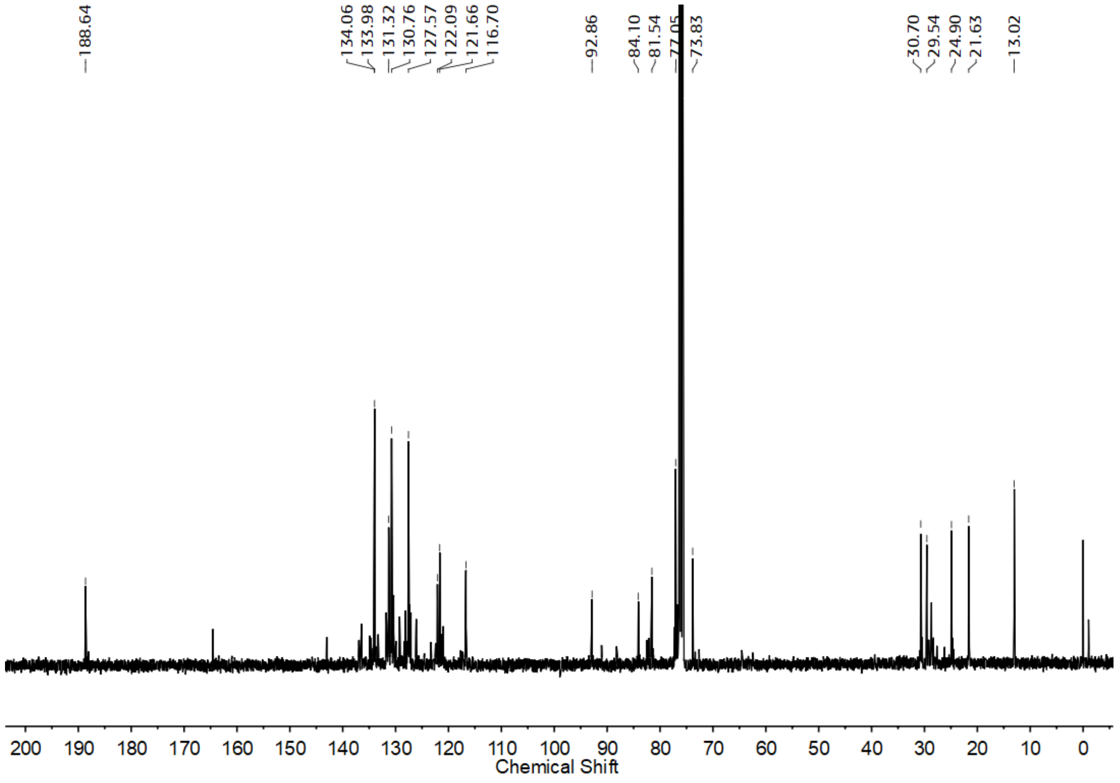

Figure S18. ^{13}C NMR spectrum of compound **11** in $CDCl_3$.

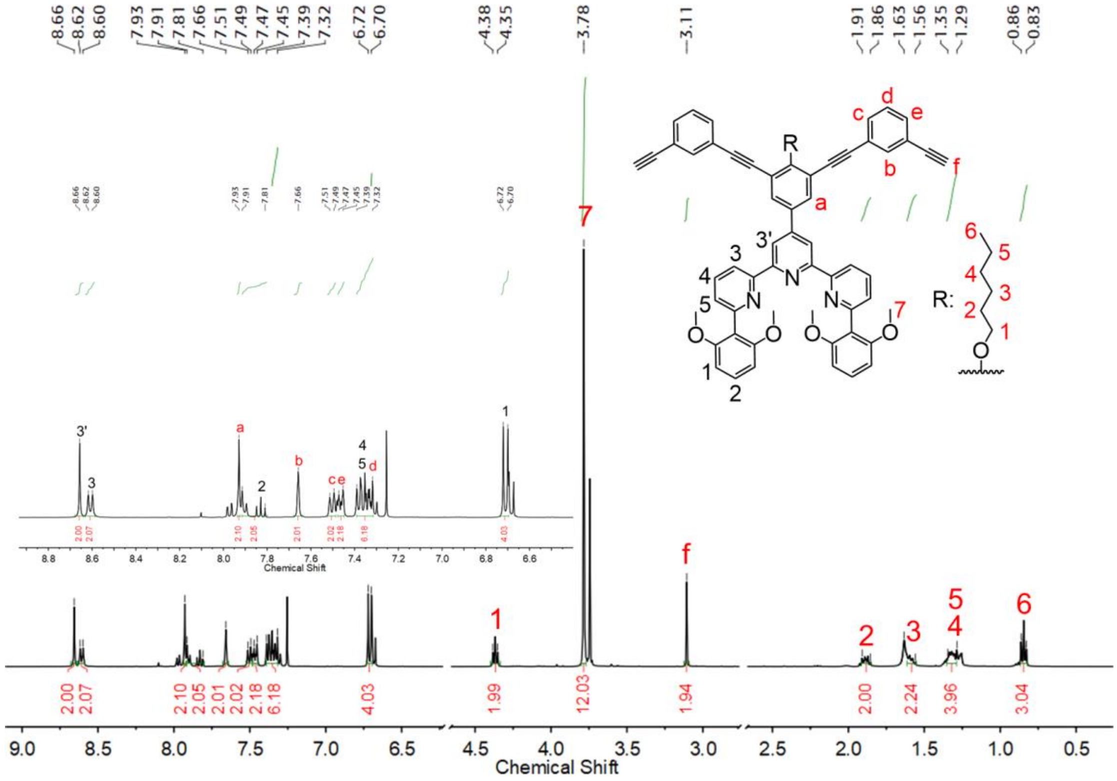

Figure S19. ^{1}H NMR spectrum of compound **12** in $CDCl_3$.

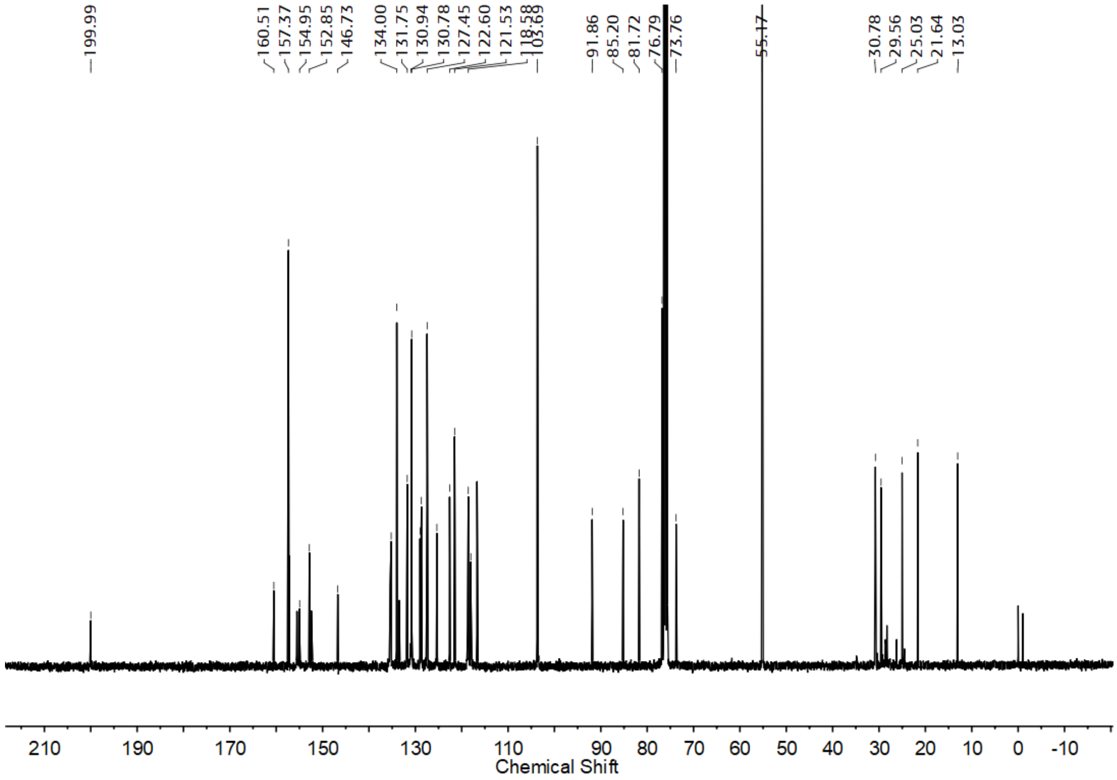

Figure S20. ^{13}C NMR spectrum of compound **12** in $CDCl_3$.

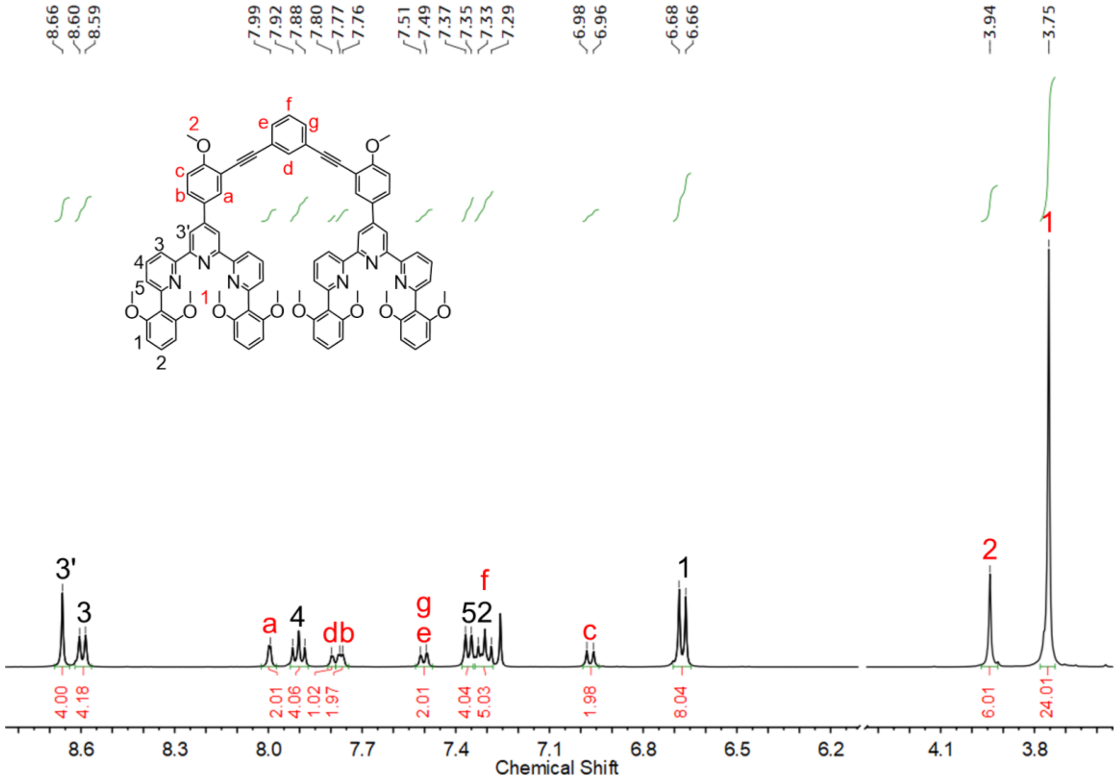

Figure S21. ^{1}H NMR spectrum of compound **L1** in $CDCl_3$.

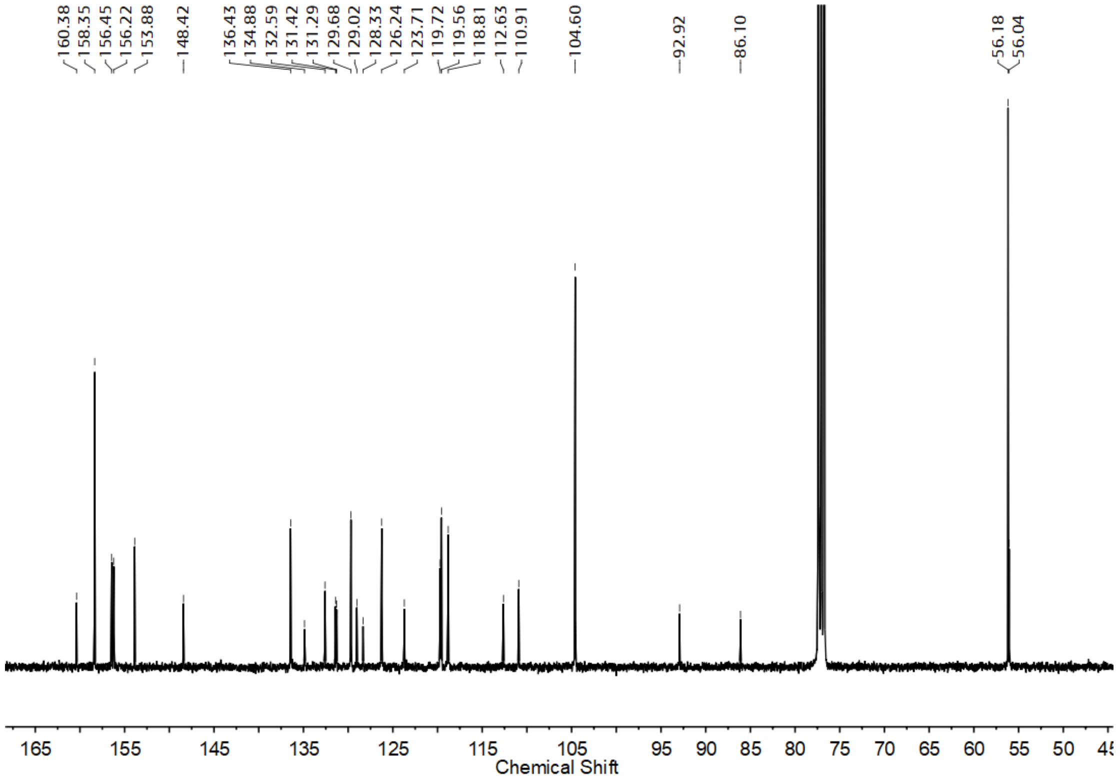

Figure S22. ^{13}C NMR spectrum of compound **L1** in $CDCl_3$.

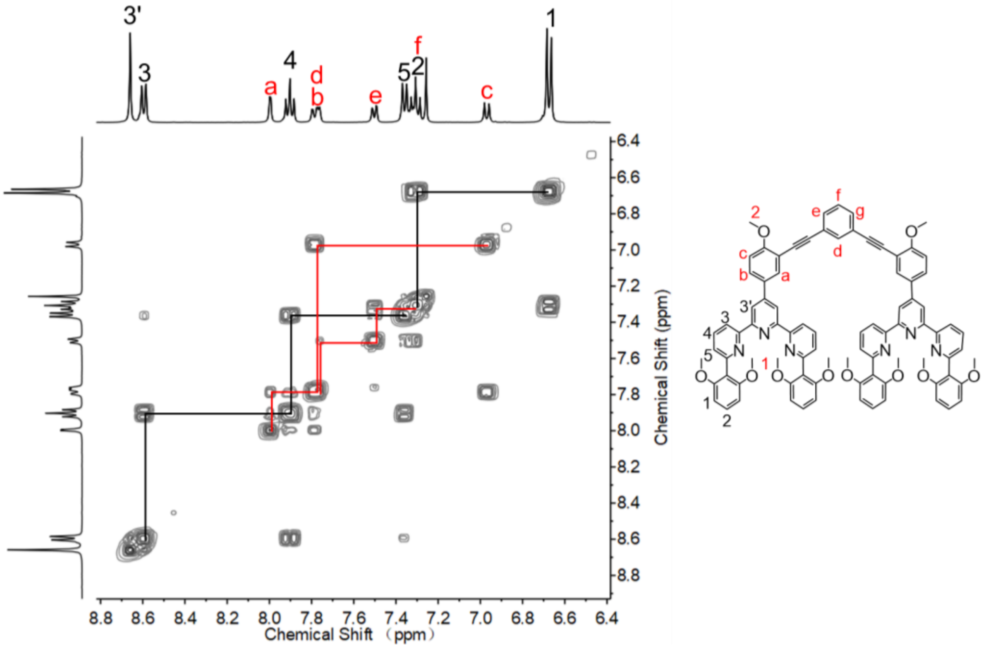

Figure S23. COSY spectrum of compound **L1** in $CDCl_3$.

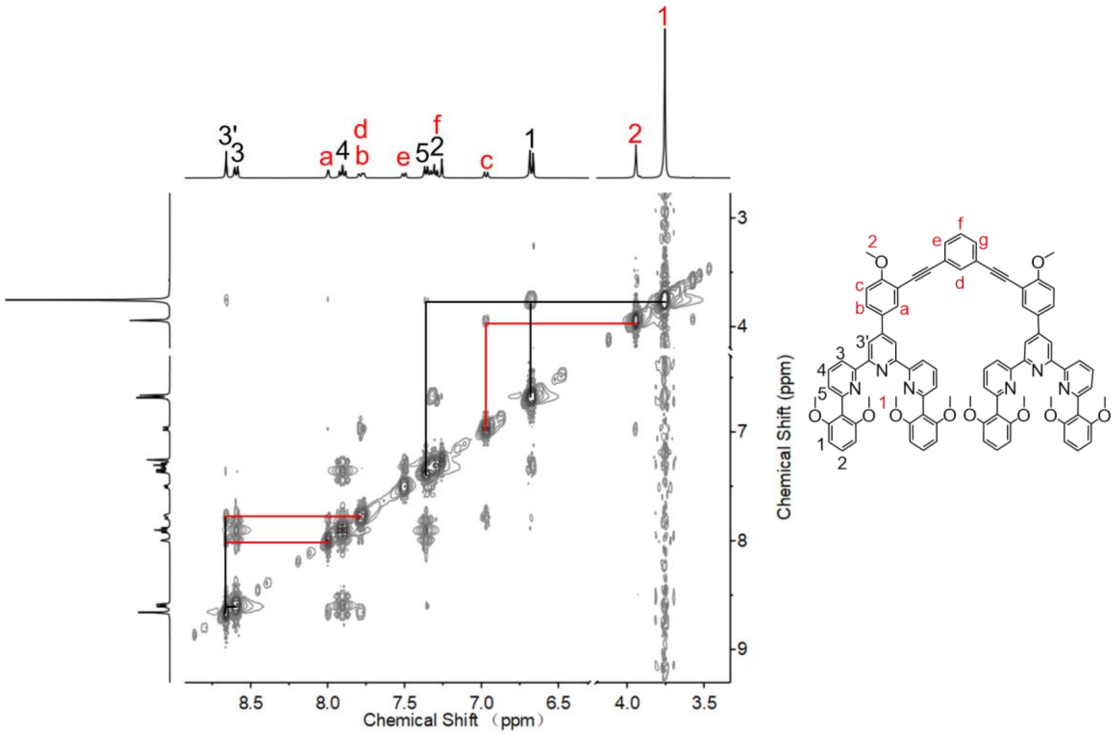

Figure S24. NOESY spectrum of compound **L1** in $CDCl_3$.

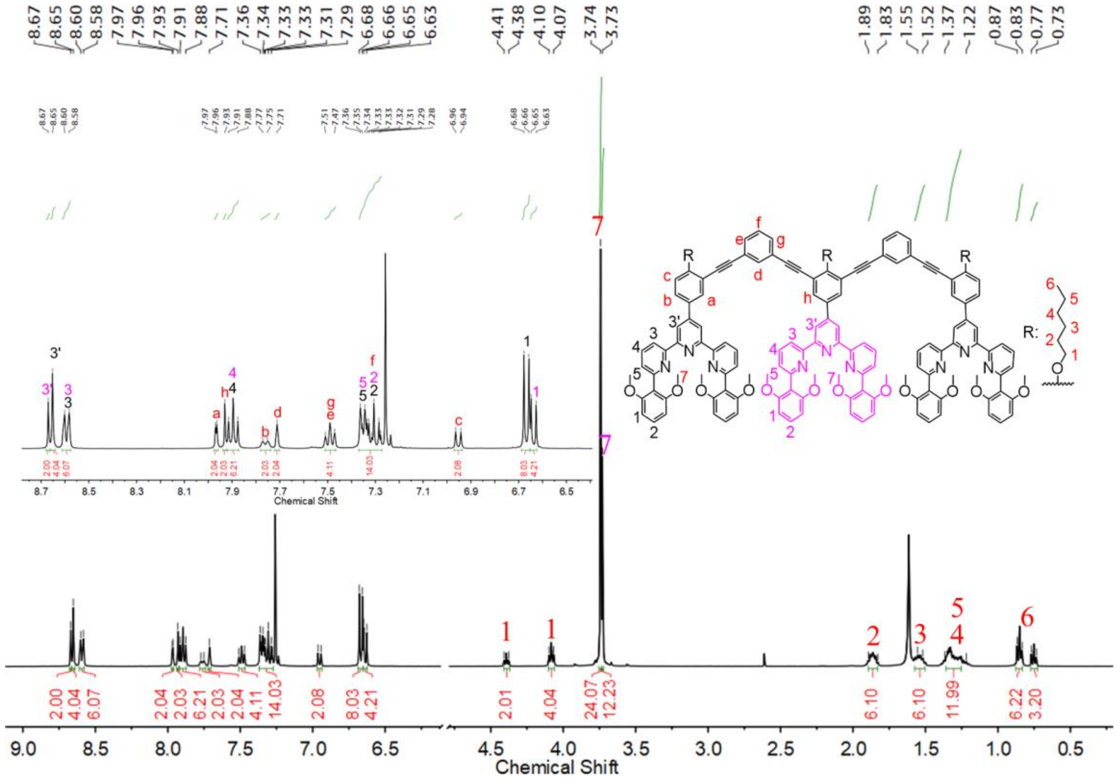

Figure S25. ^{1}H NMR spectrum of compound **L2** in $CDCl_3$.

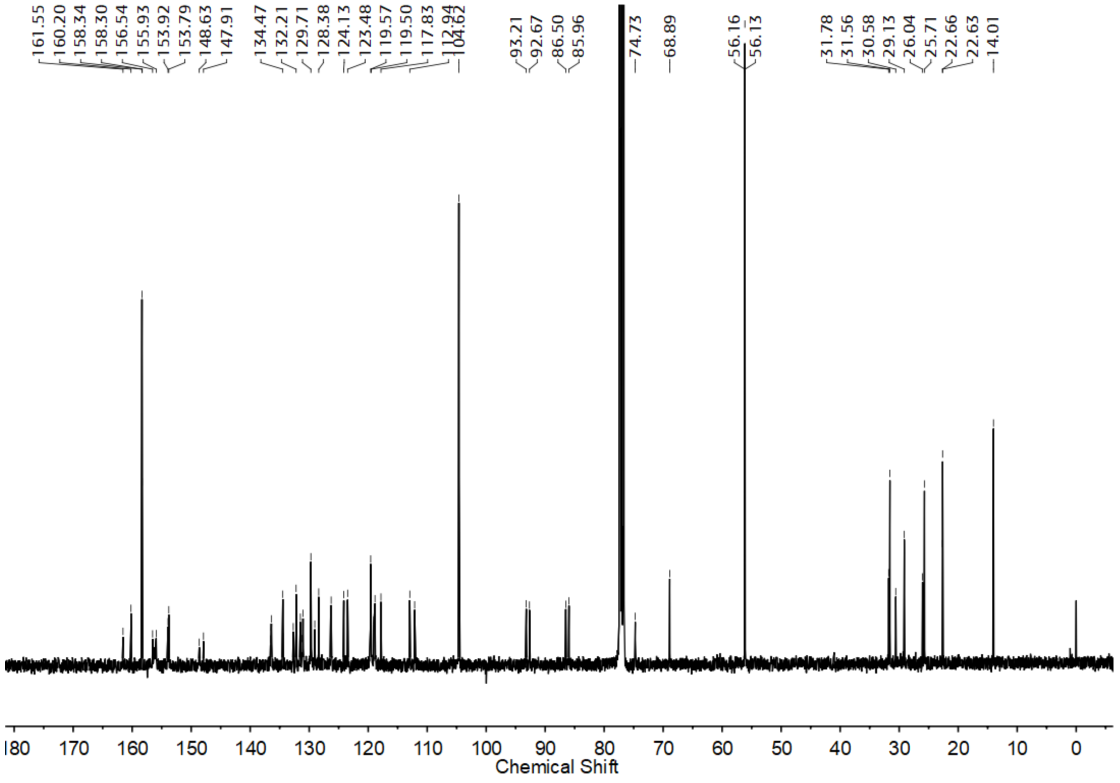

Figure S26. ^{13}C NMR spectrum of compound **L2** in $CDCl_3$.

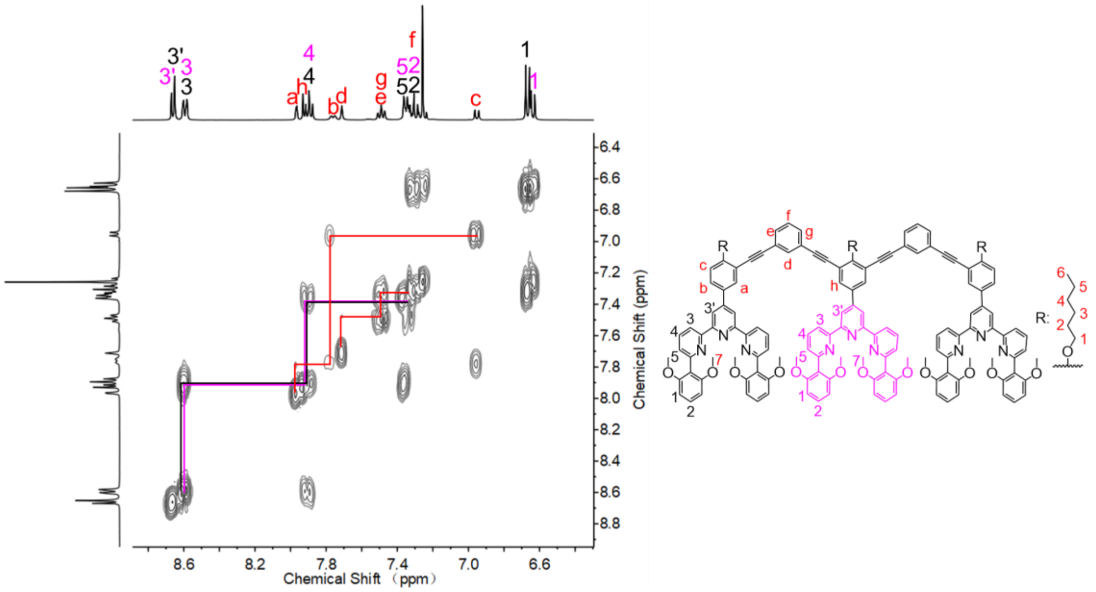

Figure S27. COSY spectrum of compound **L2** in $CDCl_3$.

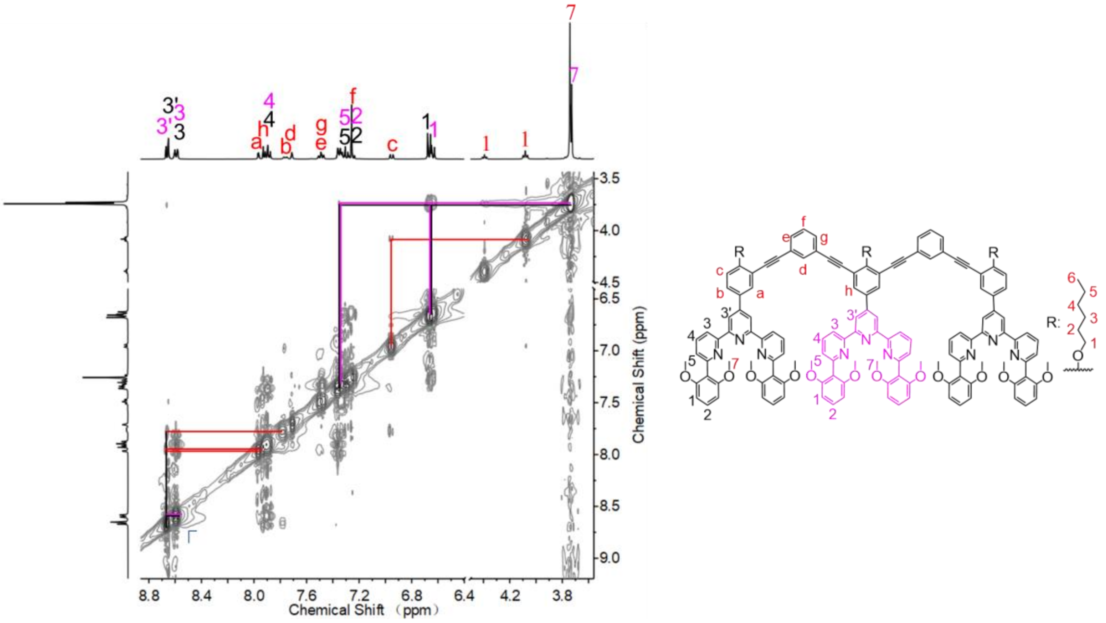

Figure S28. NOESY spectrum of compound **L2** in CD_3CN.

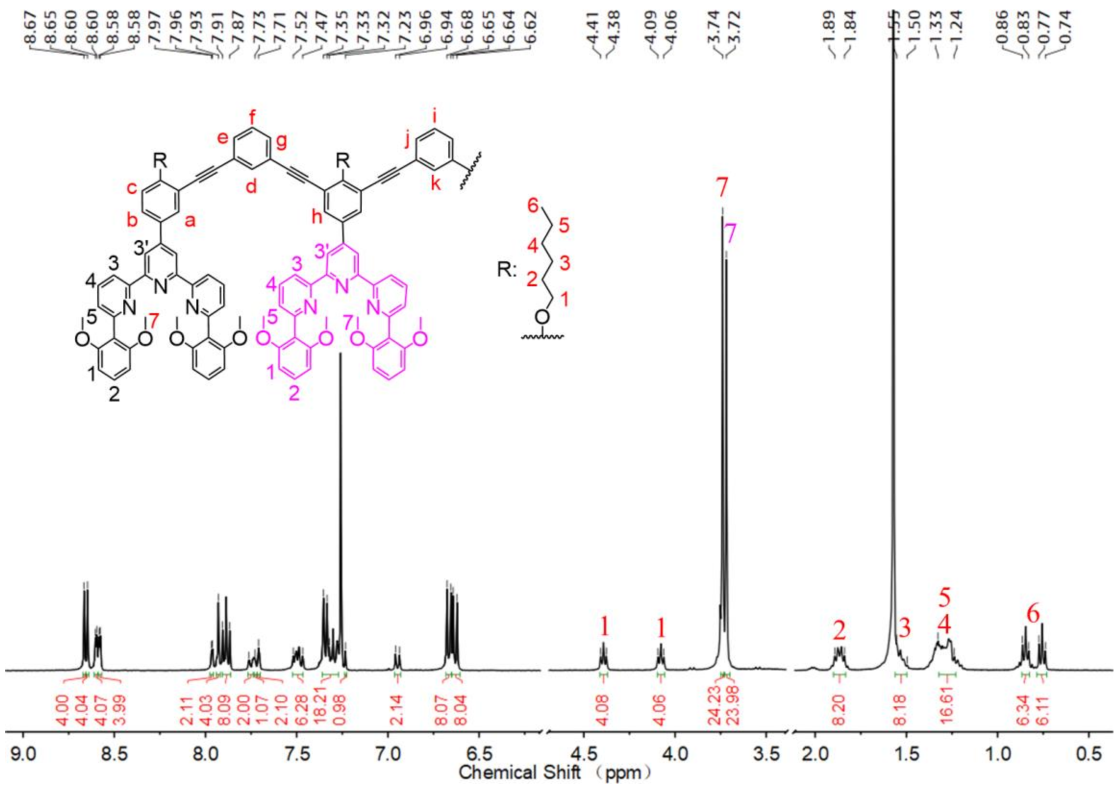

Figure S29. ^{1}H NMR spectrum of compound **L3** in $CDCl_3$.

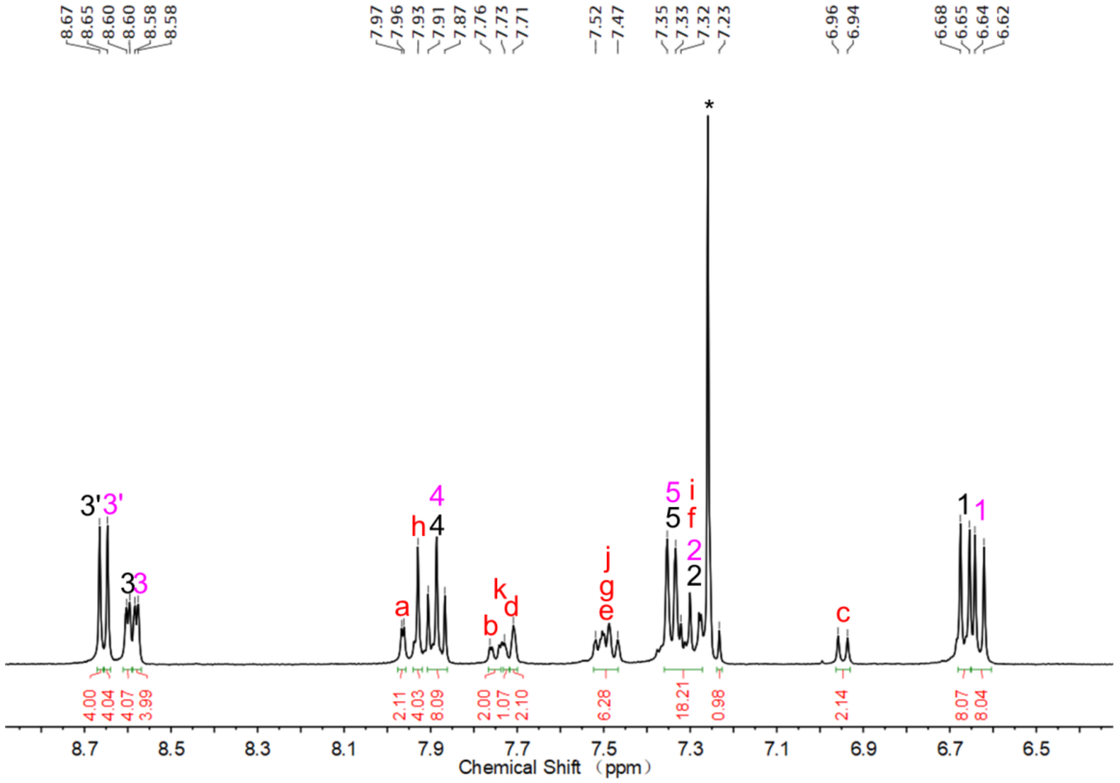

Figure S30. ^{1}H NMR spectrum of compound **L3** in $CDCl_3$.

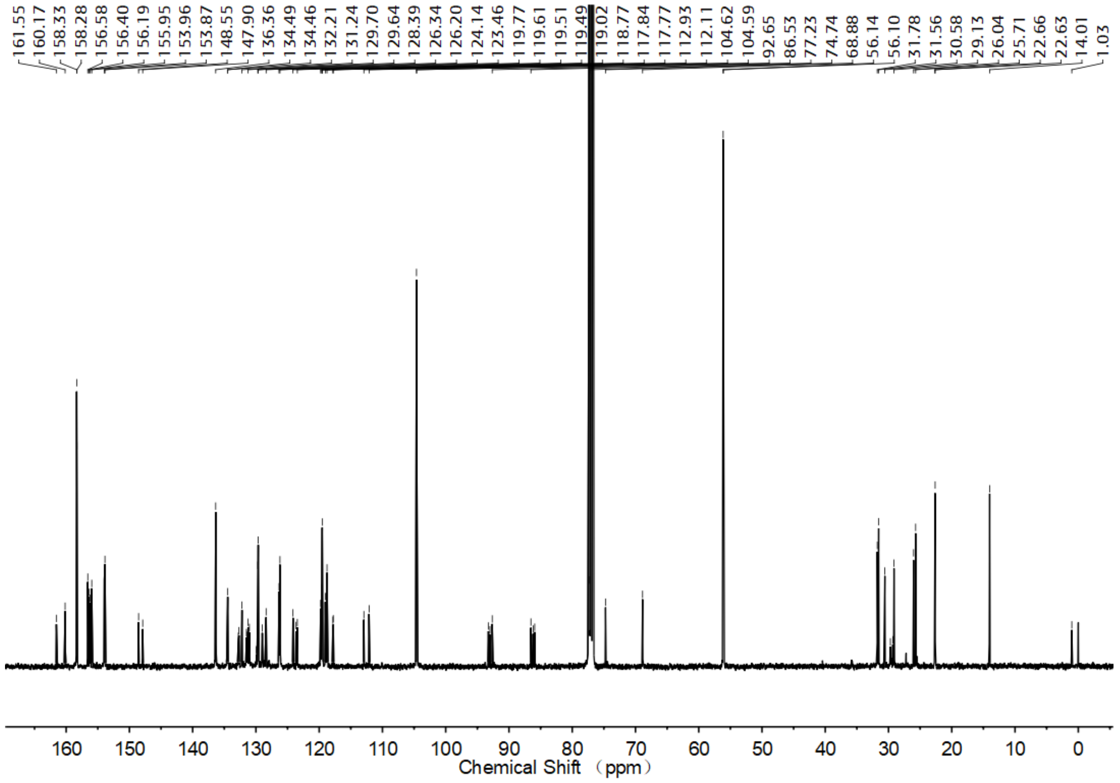

Figure S31. ^{13}C NMR spectrum of compound **L3** in $CDCl_3$.

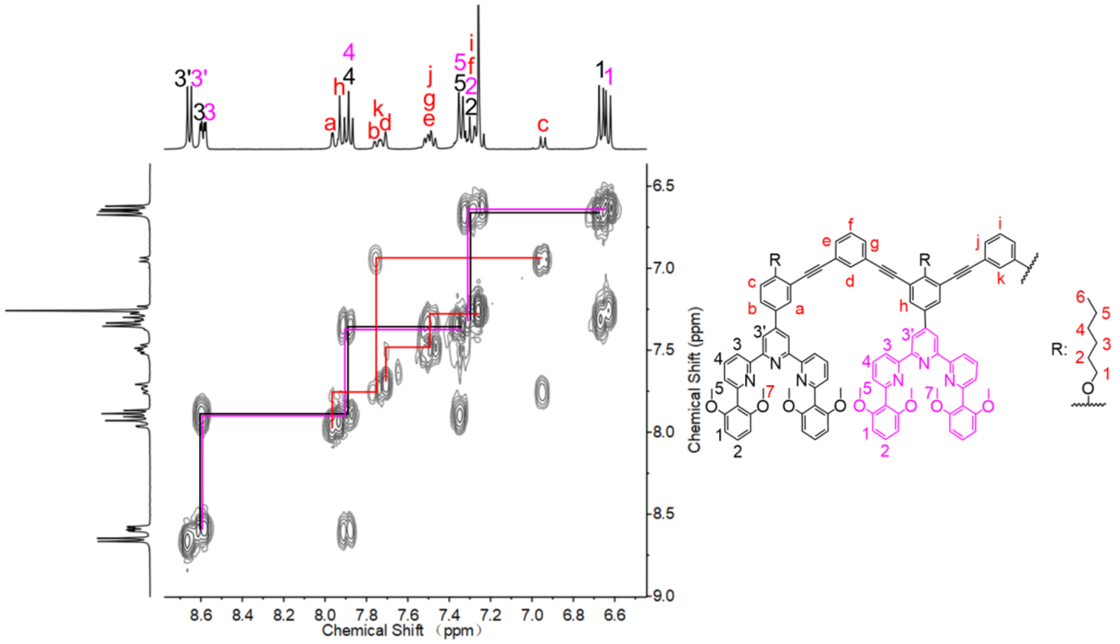

Figure S32. COSY spectrum of compound **L3** in $CDCl_3$.

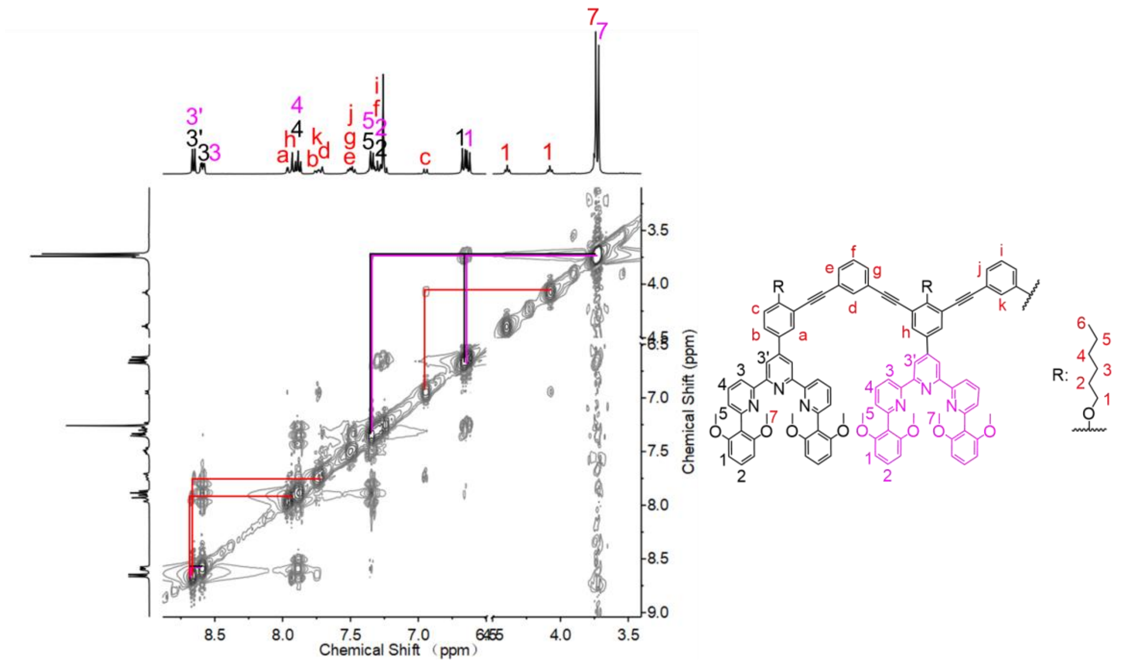

Figure S33. NOESY spectrum of compound **L3** in $CDCl_3$.

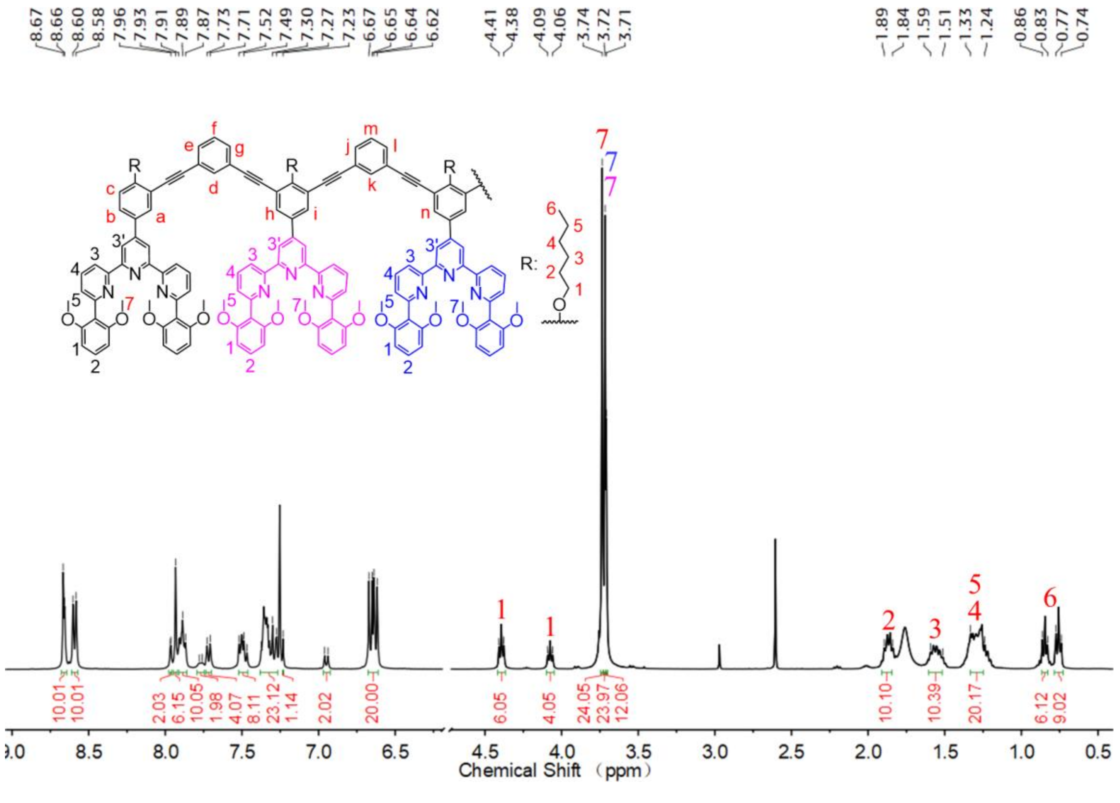

Figure S34. ^{1}H NMR spectrum of compound **L4** in $CDCl_3$.

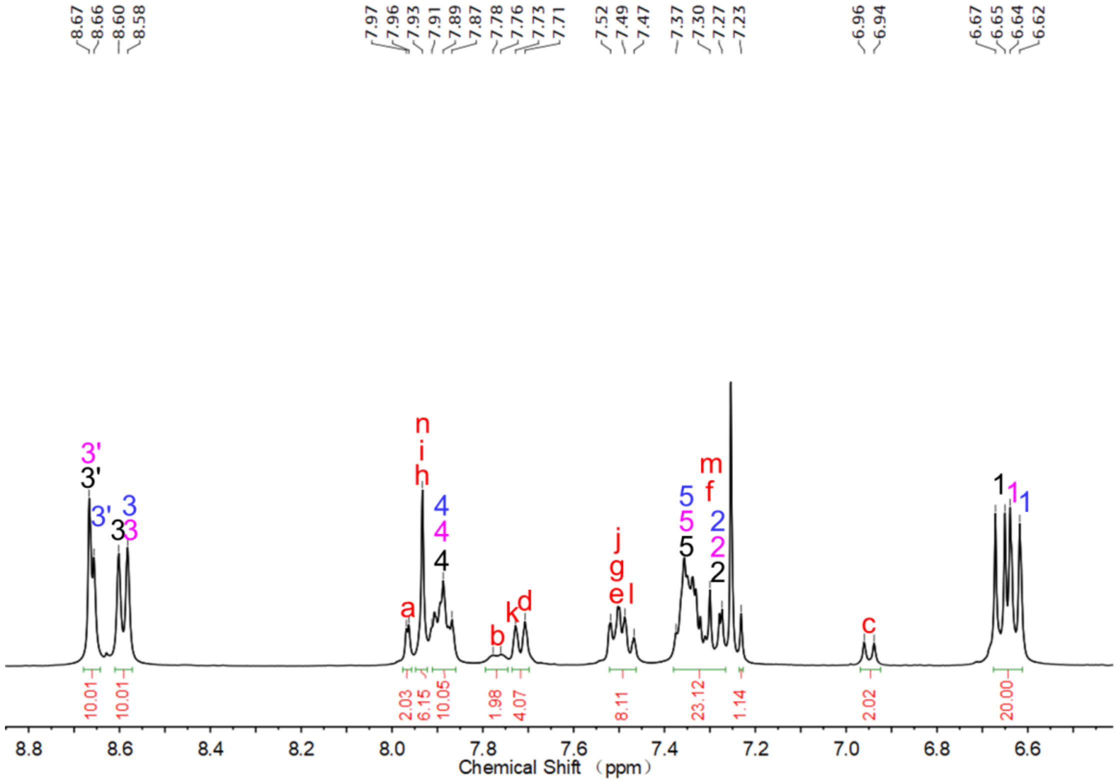

Figure S35. ^{1}H NMR spectrum of compound **L4** in $CDCl_3$.

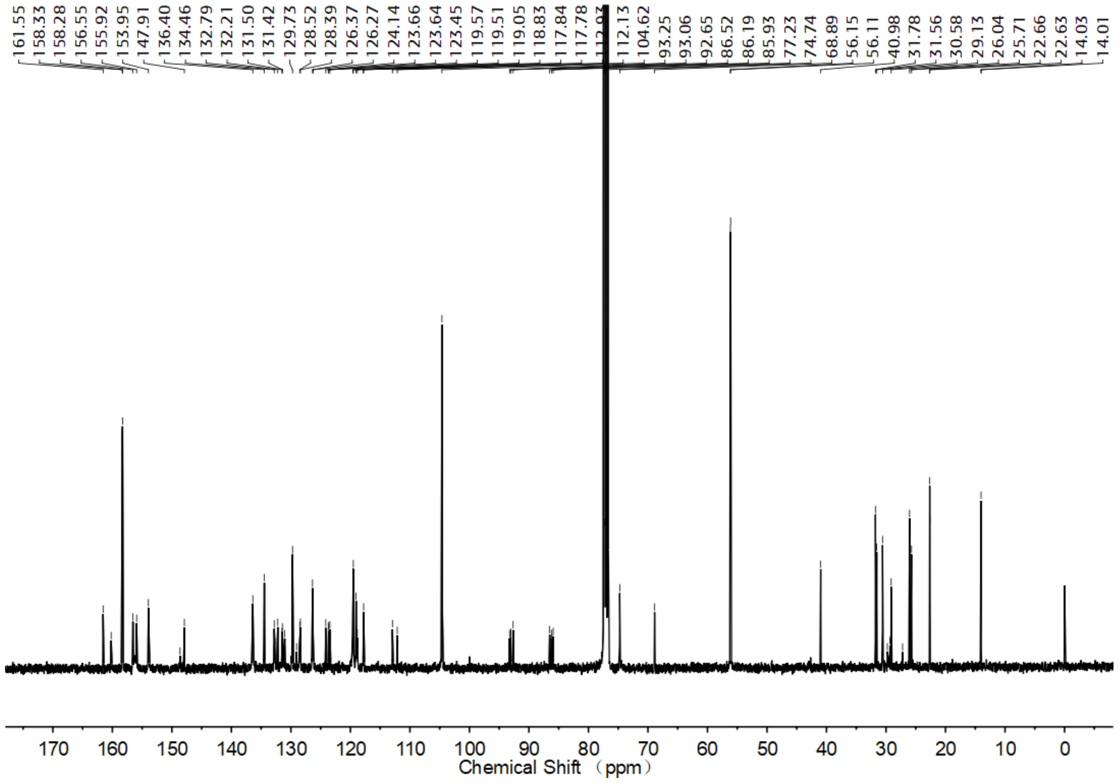

Figure S36. ^{13}C NMR spectrum of compound **L4** in $CDCl_3$.

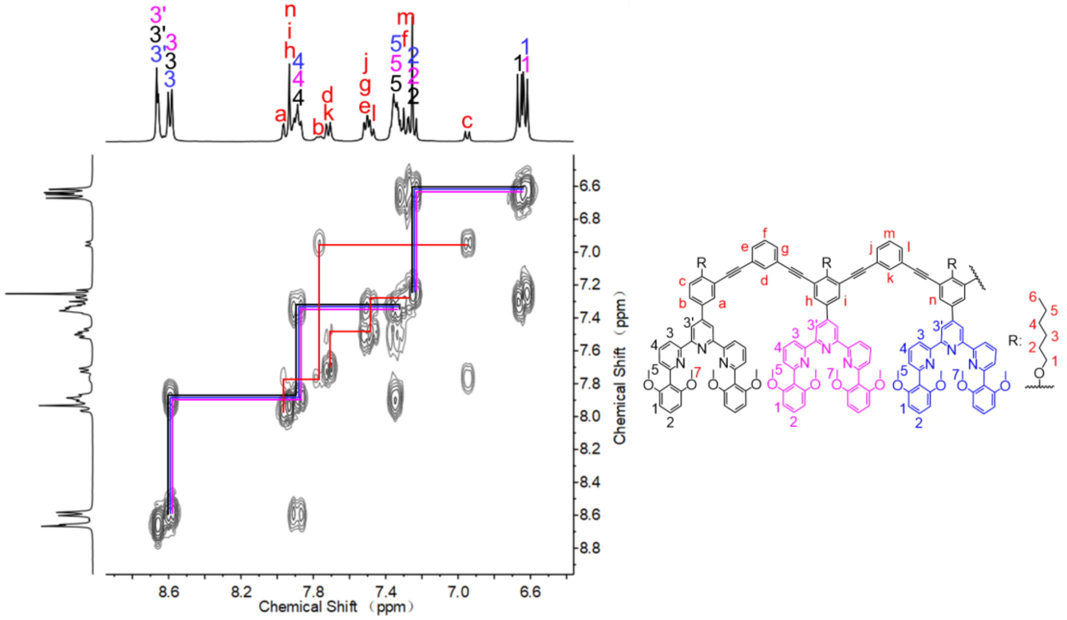

Figure S37. COSY spectrum of compound **L4** in $CDCl_3$.

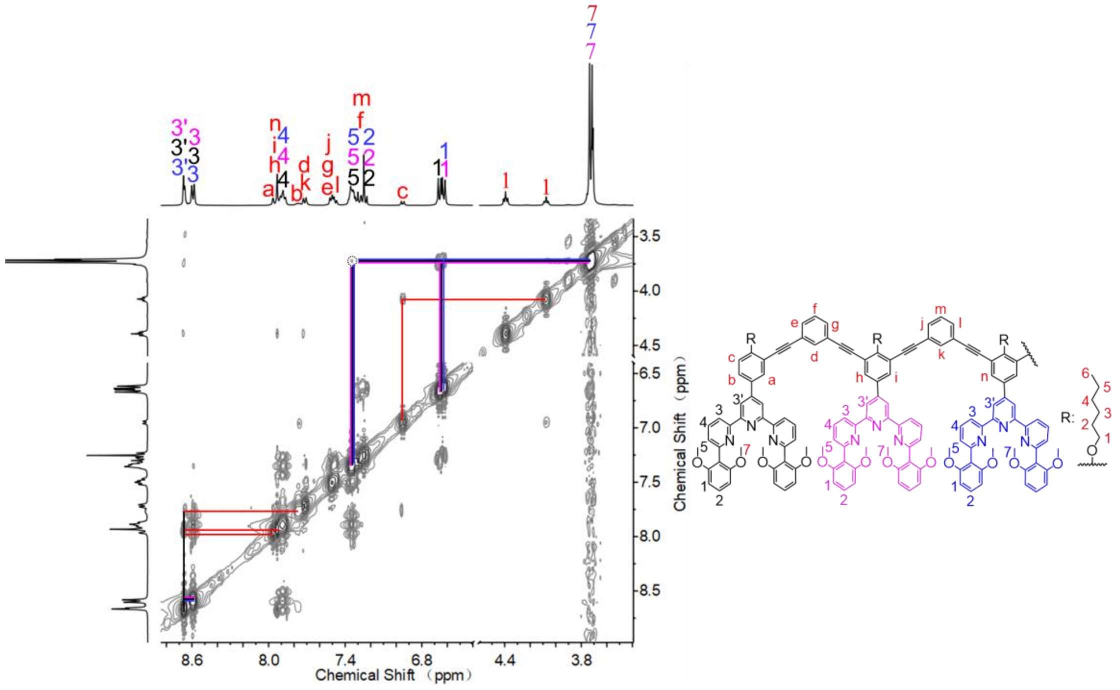

Figure S38. NOESY spectrum of compound **L4** in $CDCl_3$.

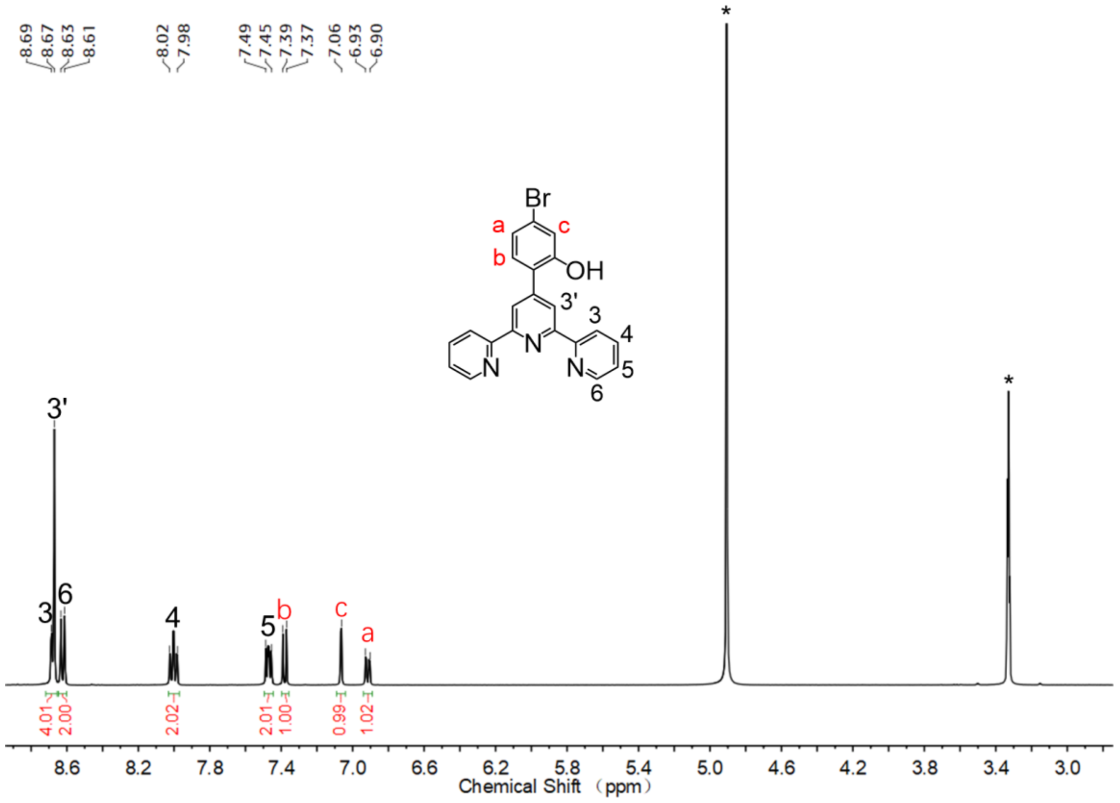

Figure S39. ^{1}H NMR spectrum of compound **13** in $CDCl_3$.

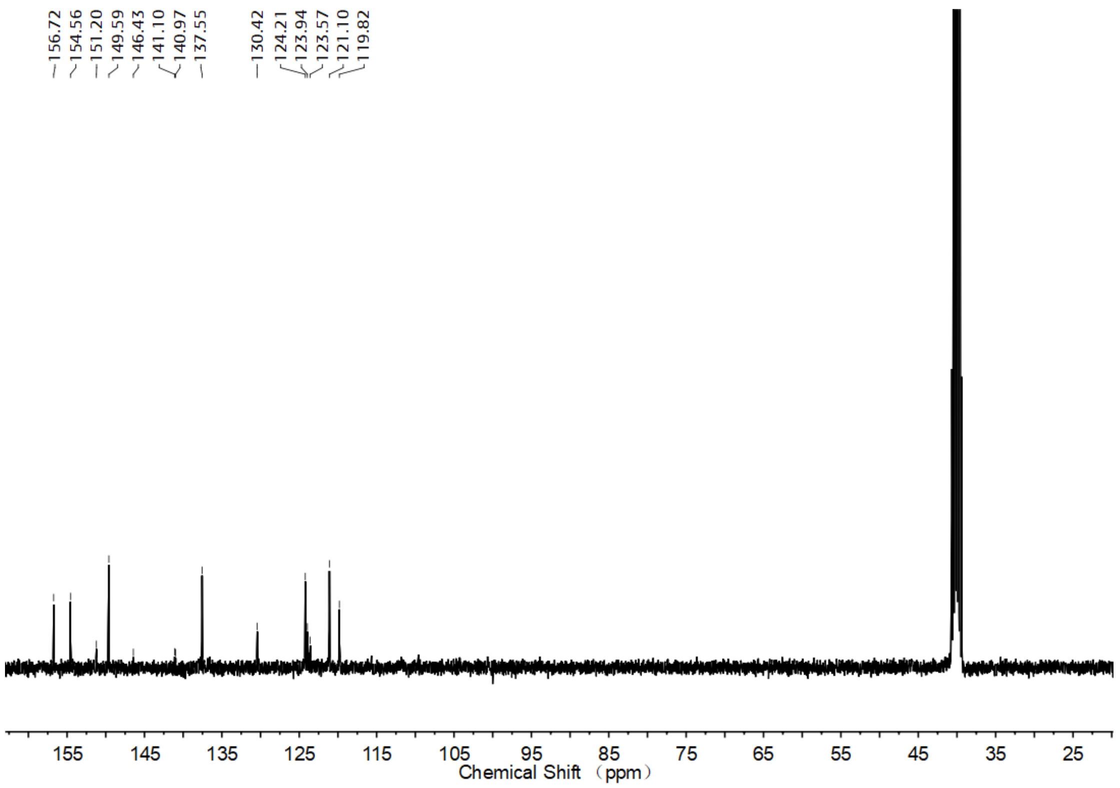

Figure S40. ^{13}C NMR spectrum of compound **13** in $CDCl_3$.

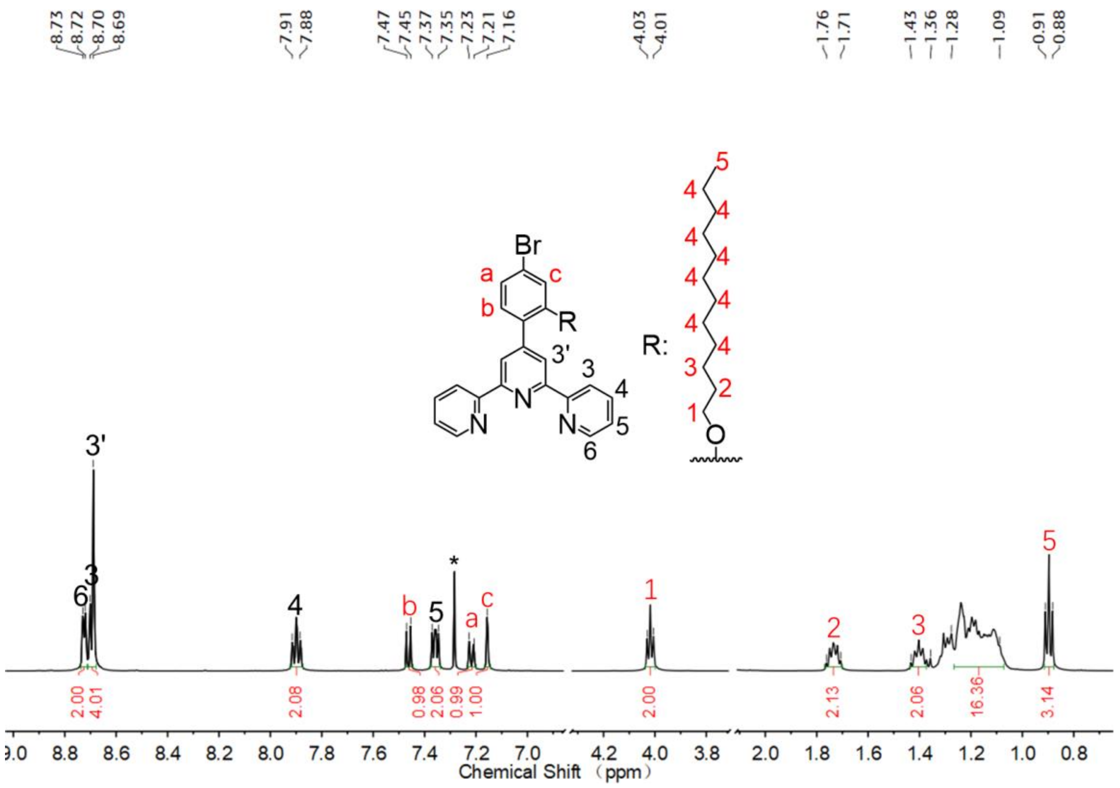

Figure S41. ^{1}H NMR spectrum of compound **14** in $CDCl_3$.

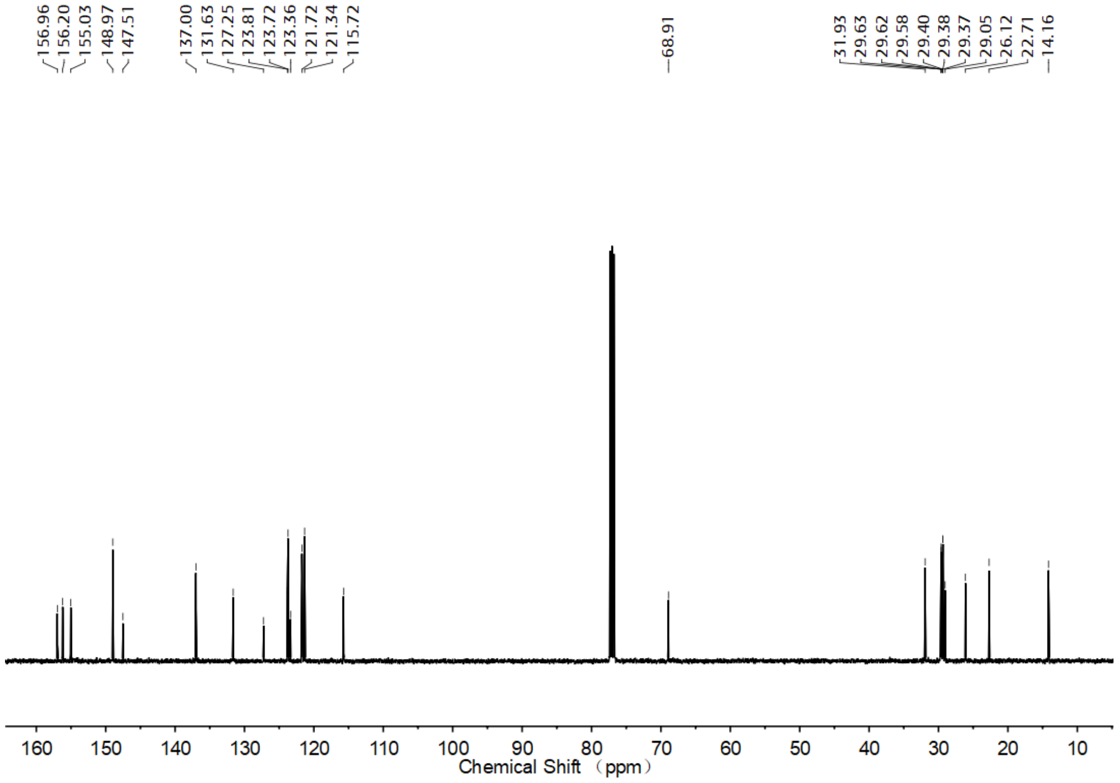

Figure S42. ^{13}C NMR spectrum of compound **14** in $CDCl_3$.

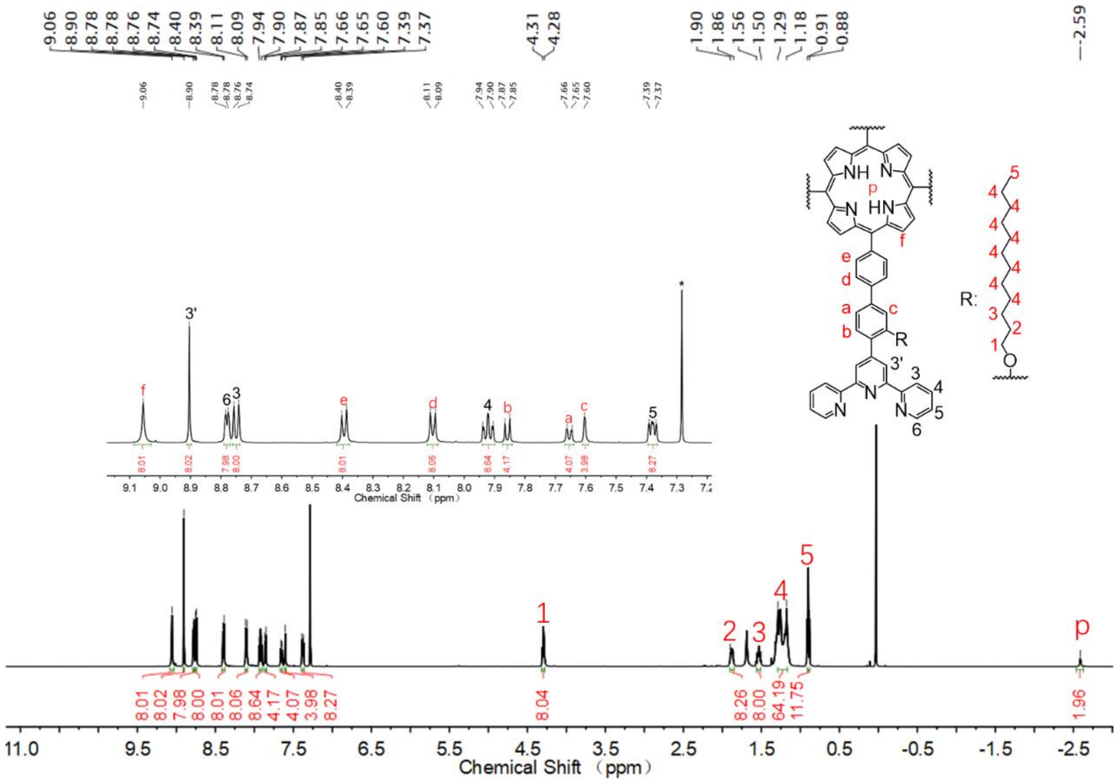

Figure S43. ^{1}H NMR spectrum of compound **L5** in $CDCl_3$.

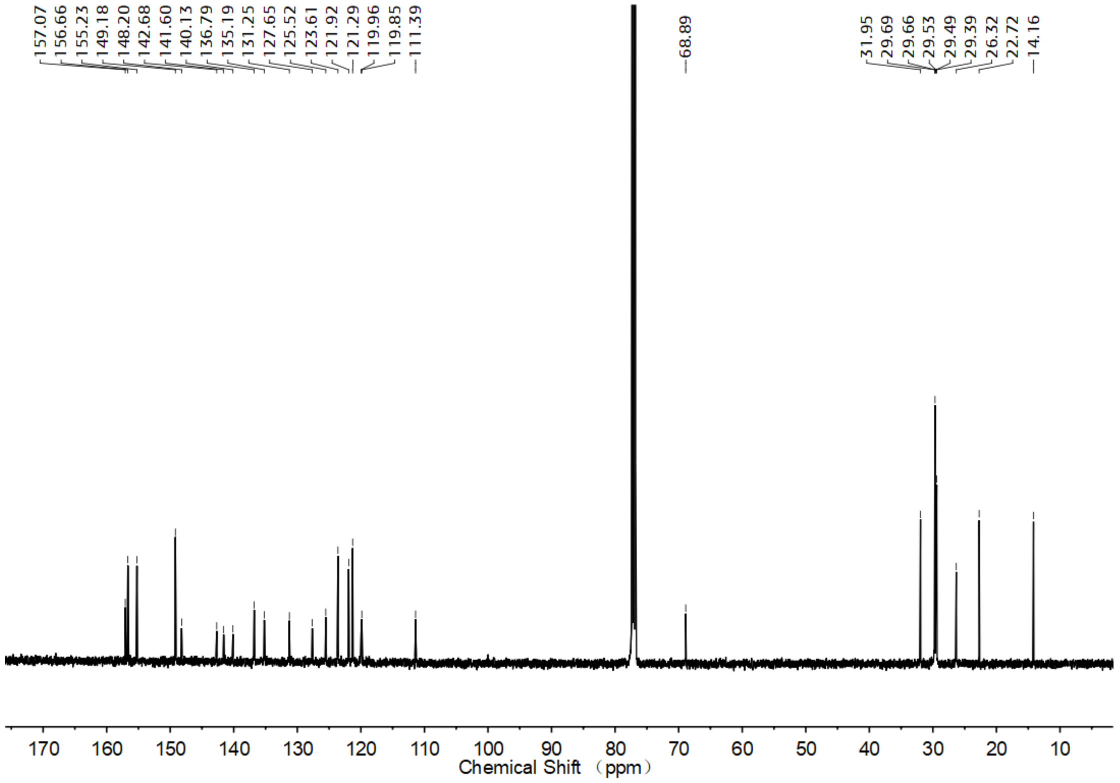

Figure S44. ^{13}C NMR spectrum of compound **L5** in $CDCl_3$.

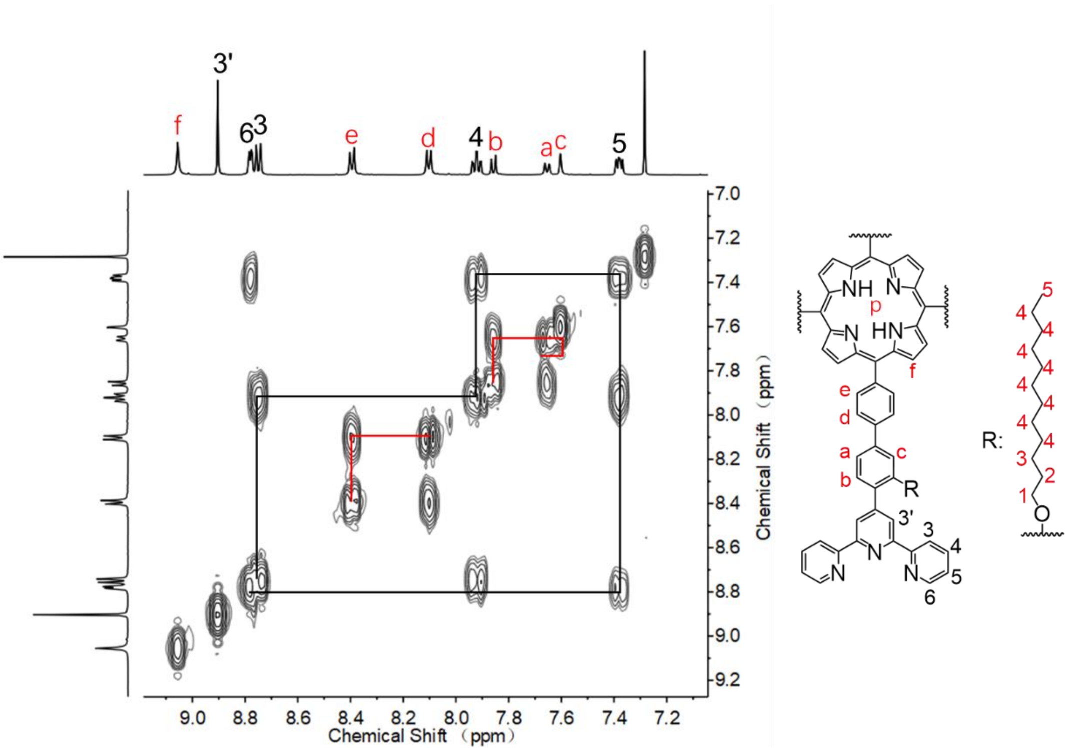

Figure S45. COSY spectrum of compound **L5** in $CDCl_3$.

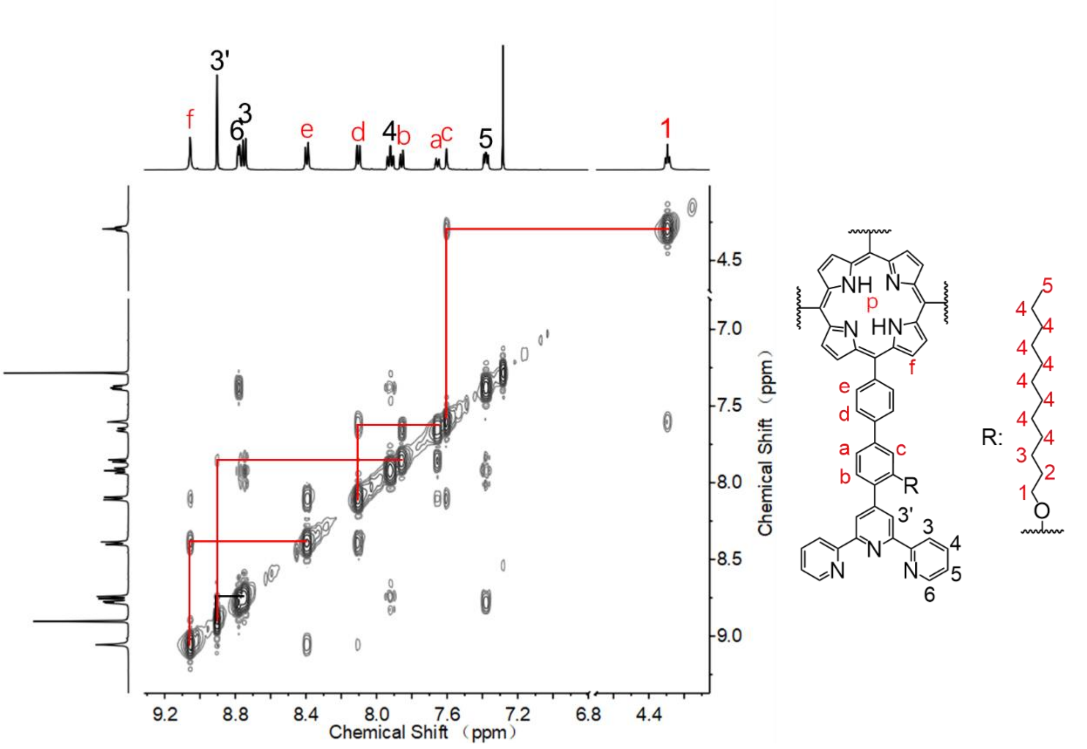

Figure S46. NOESY spectrum of compound **L5** in $CDCl_3$.

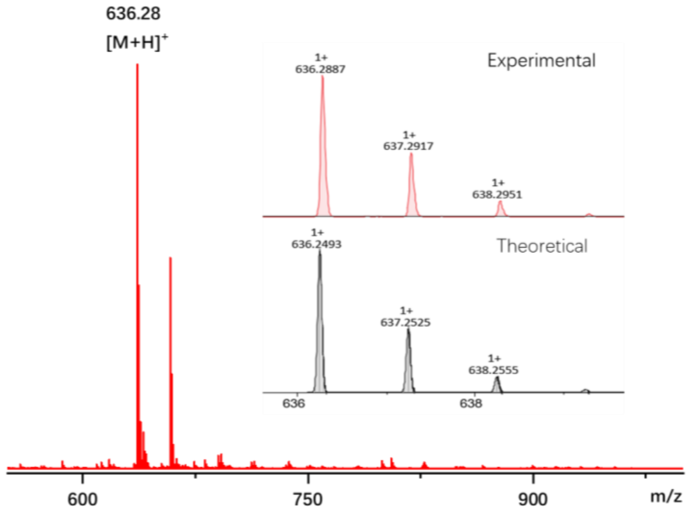

Figure S47. ESI-MS spectrum of compound **3**.

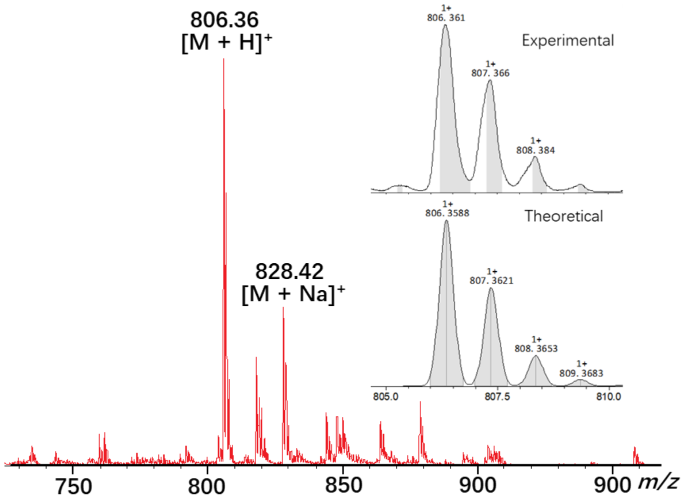

Figure S48. ESI-MS spectrum of compound **7**.

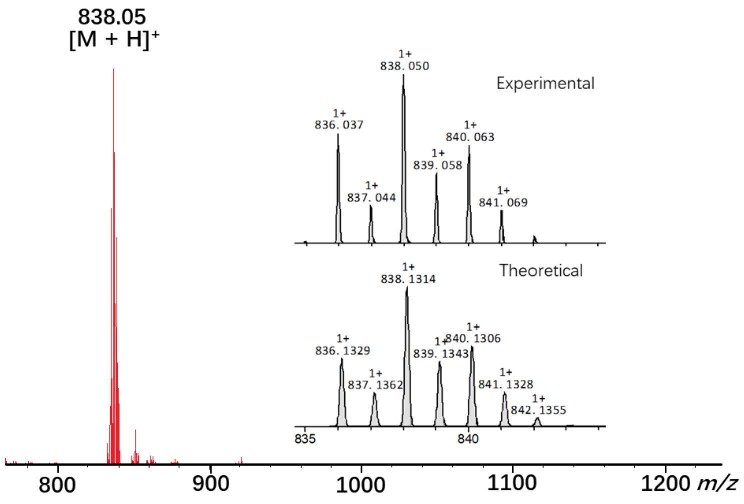

Figure S49. ESI-MS spectrum of compound **9**.

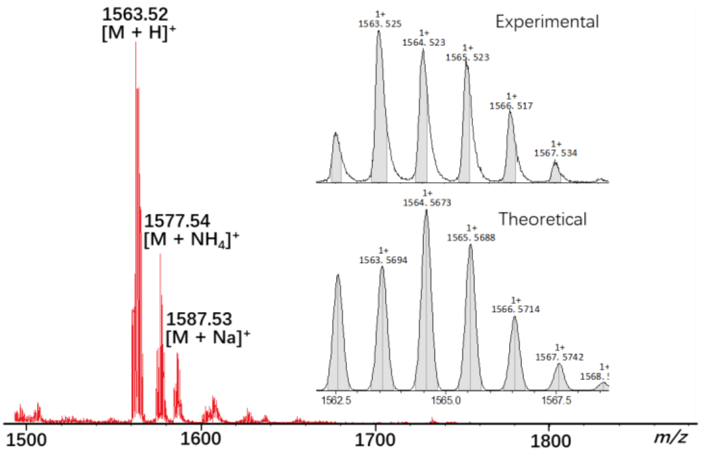

Figure S50. ESI-MS spectrum of compound **10**.

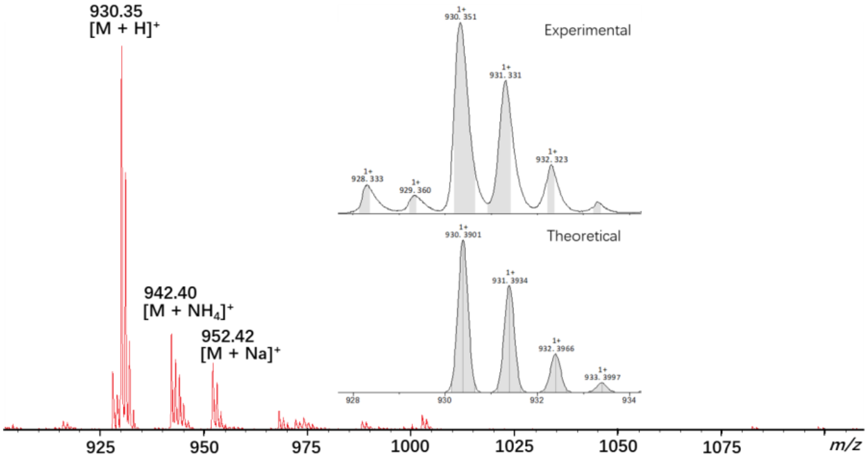

Figure S51. ESI-MS spectrum of compound **12**.

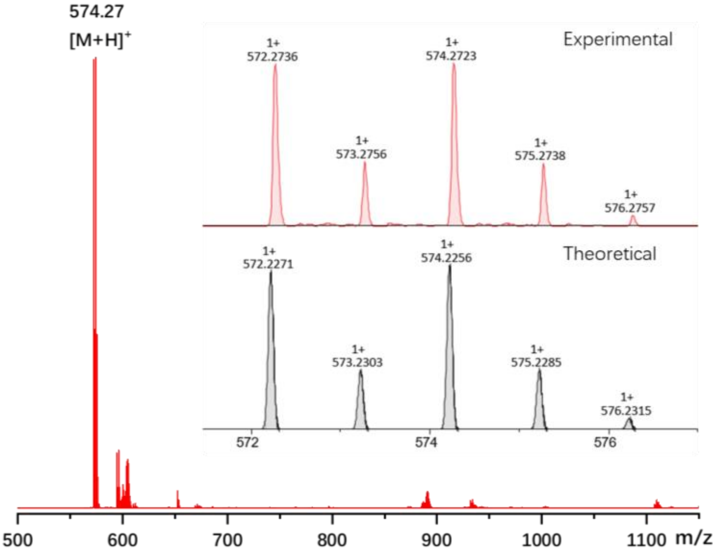

Figure S52. ESI-MS spectrum of compound **13**.

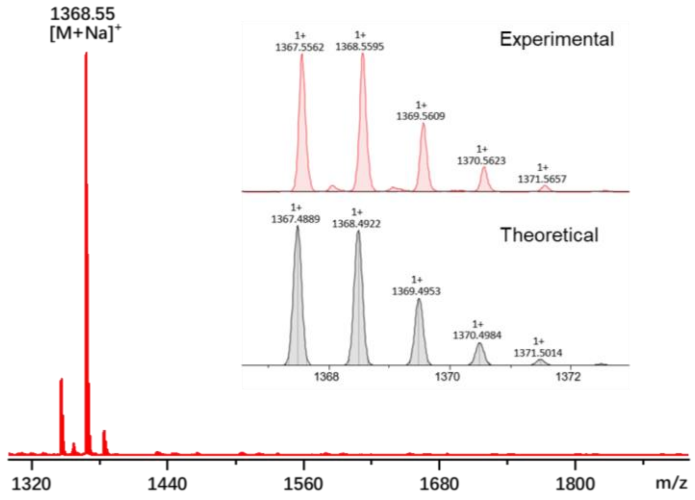

Figure S53. ESI-MS spectrum of compound **L1**.

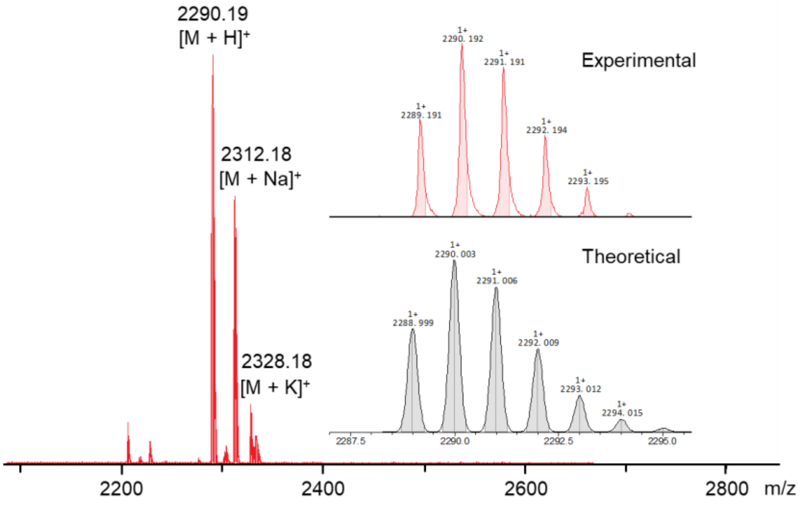

Figure S54. ESI-MS spectrum of compound **L2**.

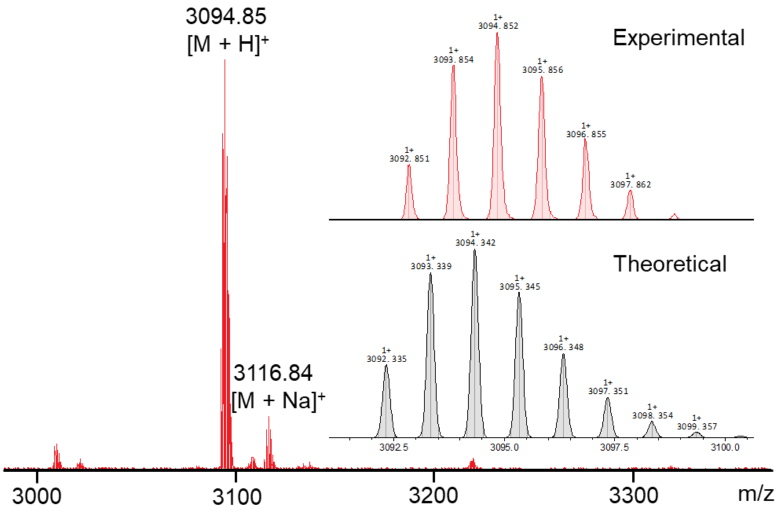

Figure S55. ESI-MS spectrum of compound **L3**.

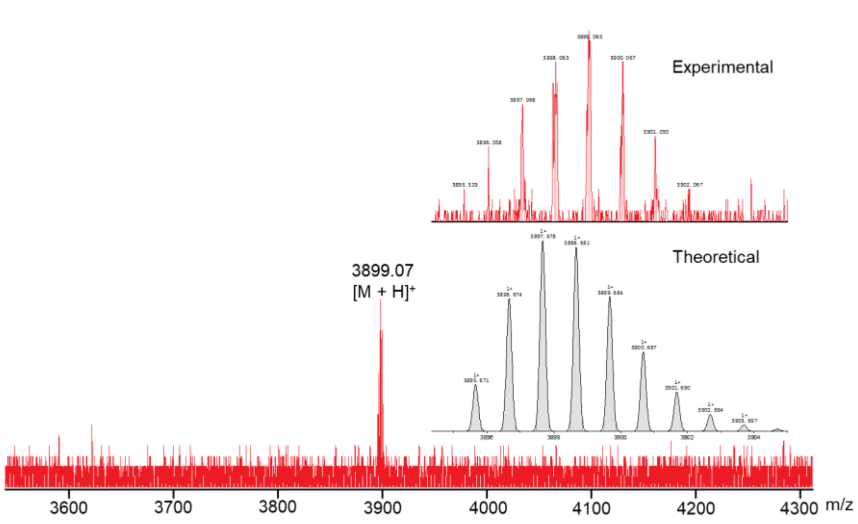

Figure S56. ESI-MS spectrum of compound **L4**.

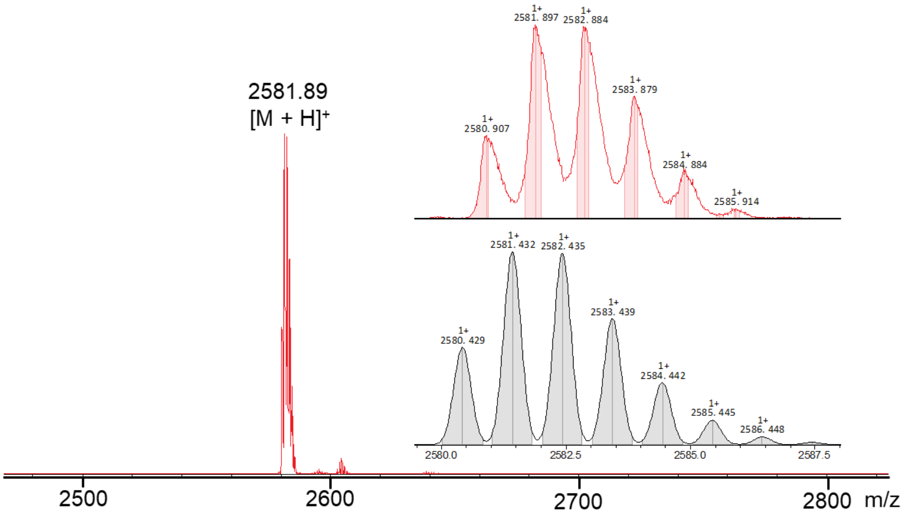

Figure S57. ESI-MS spectrum of compound **L5**.

4. Self-assembly of G0-G4.

Dissolve ligands in 20ml of a mixed solvent of CH_2Cl_2/MeOH = 2:1, a solution of $Cd(NO_3)_2 \cdot 4H_2O$ in CH_3OH (2 mL) was added. After being stirred at 85 °C for 24 h, excess NH_4PF_6 was added into the solution to precipitate the complex, which was filtered, washed with H_2O, and then dried *in vacuo*.

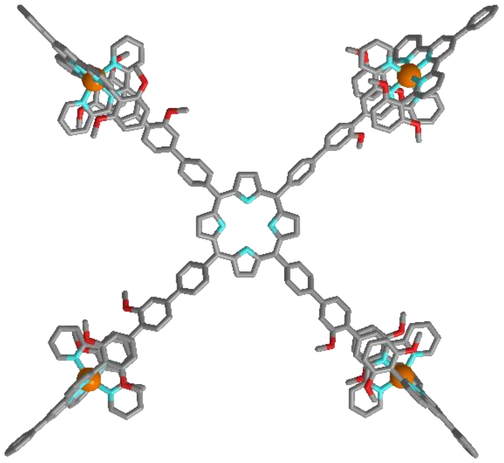

^{1}H NMR (400 MHz, CD_3CN) δ = 9.09 (s, 8H), 8.95 (s, 8H), 8.67-8.65 (d, 8H), 8.53 (s, 8H), 8.48-8.46 (d, 8H), 8.41-8.39 (d, 8H), 8.33-8.31 (d, 8H), 8.17-8.07 (m, 24H), 8.01-7.99 (d, 4H), 7.95-7.89 (m, 16H), 7.72-7.66 (m, 12H), 7.46-7.43 (t, 8H), 7.17-7.15 (d, 8H), 6.87-6.83 (t, 8H), 5.87-5.85 (d, 16H), 4.56-4.53 (t, 8H), 2.84 (s, 48H), 1.66-1.62 (m, 8H), 1.44-1.40 (m, 8H), 1.32-1.13 (m, 64H), 0.73-0.70 (m, 12H), -2.70 (s, 2H). ESI-MS (*m/z*): 669.79 $[M-8\ PF_6^-]^{8+}$ (calcd. *m/z* = 669.75), 786.19 $[M-7\ PF_6^-]^{7+}$ (calcd. *m/z* = 786.14), 941.4 $[M-6\ PF_6^-]^{6+}$ (calcd. *m/z* = 941.5), 1158.7 $[M-5\ PF_6^-]^{5+}$ (calcd. *m/z* = 1158.8).

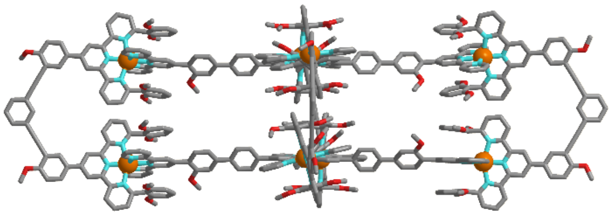

^{1}H NMR (400 MHz, CD_3CN) δ = 9.09 (s, 16H), 9.03 (s, 16H), 8.78-8.76 (d, 16H), 8.60 (s, 8H), 8.53 (s, 16H), 8.49-8.47 (d, 8H), 8.44-8.40 (t, 24H), 8.36-8.32 (m, 24H), 8.16-8.13 (t, 16H), 8.02-7.94 (m, 56H), 7.74-7.72 (d, 12H), 7.65-7.61 (t, 4H), 7.50-7.46 (m, 24H), 6.95-6.94 (d, 16H), 6.88-6.84 (t, 16H), 5.83-5.81 (d, 32H), 4.63-4.60 (t, 16H), 4.17 (s, 24H), 2.77 (s, 96H), 1.74-1.69 (m, 24H), 1.52-1.47 (m, 24H), 1.41-1.16 (m, 128H), 0.75-0.72 (t, 24H), -2.93 (s, 4H). ESI-MS (*m/z*): 1106.35 $[M-16\ PF_6^-]^{11+}$ (calcd. *m/z* = 1106.35), 1231.49 $[M-15\ PF_6^-]^{10+}$ (calcd. *m/z* = 1231.49), 1384.32 $[M-14\ PF_6^-]^{9+}$ (calcd. *m/z* = 1384.32), 1575.48 $[M-13\ PF_6^-]^{8+}$ (calcd. *m/z* = 1575.48), 1821.26 $[M-12\ PF_6^-]^{7+}$ (calcd. *m/z* = 1821.25), 2148.96 $[M-11\ PF_6^-]^{6+}$ (calcd. *m/z* = 2148.99), 2607.94 $[M-10\ PF_6^-]^{5+}$ (calcd. *m/z* = 2609.96).

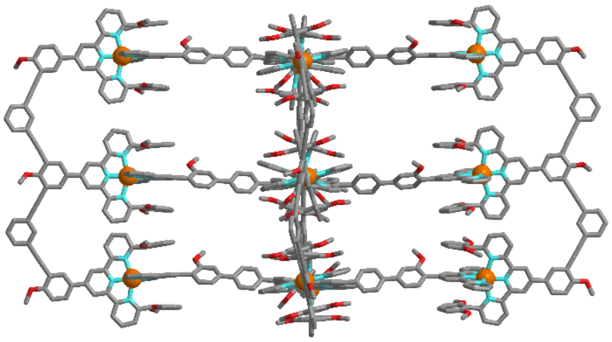

^{1}H NMR (400 MHz, CD_3CN) δ = 9.17 (s, 8H), 9.07 (s, 16H), 8.96 (s, 16H), 8.79 (s, 8H), 8.80-8.78 (d, 8H), 8.77-8.74 (d, 16H), 8.67 (s, 8H), 8.58 (s, 8H), 8.47 (s, 24H), 8.40-8.35 (m, 40H), 8.29-8.20 (m, 36H), 8.14-8.09 (m, 36H), 7.98-7.86 (m, 76H), 7.82-7.72 (m, 28H), 7.67-7.63 (m, 12H), 7.48-7.43(m, 32H), 6.88-6.87 (d, 16H), 6.83-6.79 (t, 16H), 6.76-6.72 (t, 8H), 6.54-6.52 (d, 8H), 5.78-5.75 (d, 32H), 5.64-5.62 (d, 16H), 4.75-4.72 (t, 8H), 4.62-4.51 (t, 24H), 4.38-4.35 (t, 16H), 2.72 (s, 96H), 2.57 (s, 48H), 2.09-2.01 (m, 48H), 1.77-1.64 (m, 48H), 1.58-1.44 (m, 72H), 1.38-1.11 (m, 168H), 1.04-1.00 (m, 36H), 0.74-0.67 (m, 36H), -3.01 (s, 4H), -3.27 (s, 2H). ESI-MS (*m/z*): 1213.64 $[M-16\ PF_6^-]^{16+}$ (calcd. *m/z* = 1213.22), 1304.28 $[M-15\ PF_6^-]^{15+}$ (calcd. *m/z* = 1303.76), 1407.79 $[M-14\ PF_6^-]^{14+}$ (calcd. *m/z* = 1407.25), 1527.16 $[M-13\ PF_6^-]^{13+}$ (calcd. *m/z* = 1526.65), 1666.55 $[M-12\ PF_6^-]^{12+}$ (calcd. *m/z* = 1665.96), 1831.28 $[M-11\ PF_6^-]^{11+}$ (calcd. *m/z* = 1830.59), 2028.91 $[M-10\ PF_6^-]^{10+}$ (calcd. *m/z* = 2028.15), 2270.59 $[M-9\ PF_6^-]^{9+}$ (calcd. *m/z* = 2269.61), 2572.41 $[M-8\ PF_6^-]^{8+}$ (calcd. *m/z* = 2571.43), 2960.61 $[M-7\ PF_6^-]^{7+}$ (calcd. *m/z* = 2959.50).

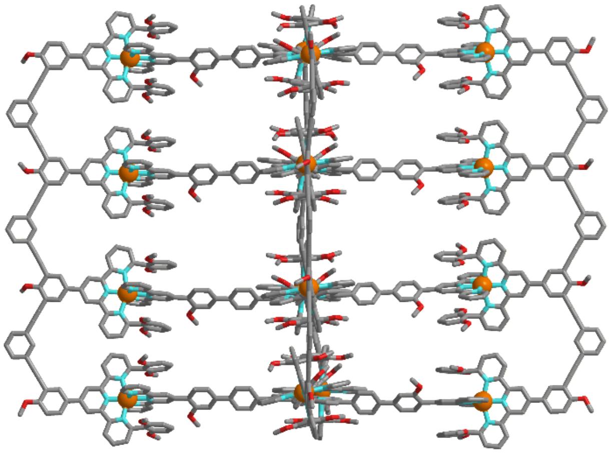

^{1}H NMR (400 MHz, CD_3CN) δ = 9.11 (s, 16H), 9.03 (s, 16H), 8.92 (s, 16H), 8.77-8.70 (m, 48H), 8.61 (s, 16H), 8.54 (s, 8H), 8.42 (s, 16H), 8.35-8.28 (m, 64H), 8.24-8.22 (d, 16H), 8.16-8.13 (m, 32H), 8.09-8.03 (m, 48H), 7.94-7.91 (m, 48H), 7.88-7.85 (m, 32H), 7.81-7.69 (m, 48H), 7.66-7.60 (m, 16H), 7.43-7.37 (m, 40H), 6.85-6.83 (d, 16H), 6.78-6.74 (t, 16H), 6.69-6.65 (t, 16H), 6.45-6.43 (d, 16H), 5.73-5.71 (d, 32H), 5.58-5.56 (d, 32H), 4.72-4.69 (t, 16H), 4.54-4.50 (t, 32H), 4.35-4.32 (t, 16H), 2.68 (s, 96H), 2.51 (s, 96H), 2.04-1.97 (m, 64H), 1.73-1.58 (m, 64H), 1.53-1.41 (m, 80H), 1.31-1.05 (m, 240H), 1.01-0.97 (m, 48H), 0.68-0.61 (m, 48H), -3.03 (s, 4H), -3.32 (s, 4H). ESI-MS (*m/z*): 1179.66 $[M-22\ PF_6^-]^{22+}$ (calcd. *m/z* = 1179.46), 1242.70 $[M-21\ PF_6^-]^{21+}$ (calcd. *m/z* = 1242.53), 1312.12 $[M-20\ PF_6^-]^{20+}$ (calcd. *m/z* = 1311.91), 1388.84 $[M-19\ PF_6^-]^{19+}$ (calcd. *m/z* = 1388.59), 1473.98 $[M-18\ PF_6^-]^{18+}$ (calcd. *m/z* = 1473.79), 1569.27 $[M-17\ PF_6^-]^{17+}$ (calcd. *m/z* = 1569.01), 1676.35 $[M-16\ PF_6^-]^{16+}$ (calcd. *m/z*

= 1676.13), 1797.69 [M-15 PF_6^-]$^{15+}$ (calcd. *m/z* = 1797.54), 1936.71 [M-14 PF_6^-]$^{14+}$ (calcd. *m/z* = 1936.30), 2096.76 [M-13 PF_6^-]$^{13+}$ (calcd. *m/z* = 2096.40), 2283.66 [M-12 PF_6^-]$^{12+}$ (calcd. *m/z* = 2283.18), 2504.42 [M-11 PF_6^-]$^{11+}$ (calcd. *m/z* = 2503.92), 2769.37 [M-10 PF_6^-]$^{10+}$ (calcd. *m/z* = 2768.81).

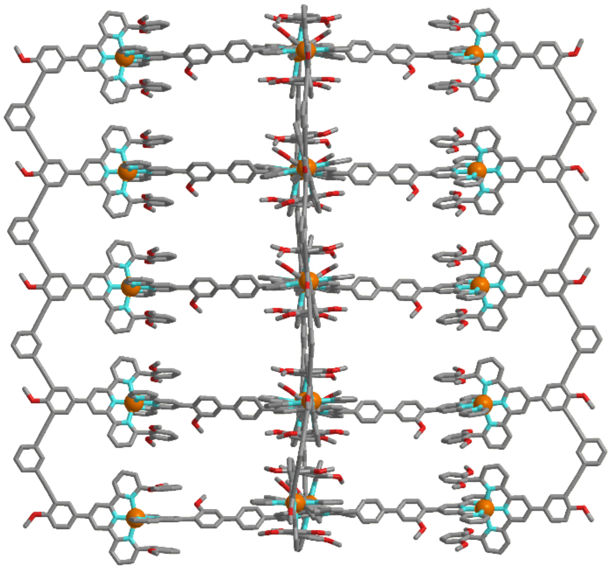

^{1}H NMR (400 MHz, CD_3CN) δ 9.11 (s, 24H), 9.03 (s, 16H), 8.91 (s, 16H), 8.75-8.71 (m, 64H), 8.62 (s, 24H), 8.54 (s, 8H), 8.41 (s, 16H), 8.35-8.27 (m, 72H), 8.23-8.21 (m, 20H), 8.16-8.12 (m, 40H), 8.07-8.02 (m, 60H), 7.93-7.84 (m, 116H), 7.79-7.59 (m, 88H), 7.41-7.38 (m, 48H), 6.84-6.82 (d, 16H), 6.77-6.73 (t, 16H), 6.66-6.63 (t, 24H), 6.43-6.41 (d, 16H), 6.37-6.36 (d, 8H), 5.72-5.70 (d, 32H), 5.55-5.51 (m, 48H), 4.72-4.69 (t, 24H), 4.53-4.46 (m, 40H), 4.35-4.30 (t, 16H), 2.67 (s, 96H), 2.50 (s, 96H), 2.47 (s, 48H), 1.87-1.70 (m, 80H), 1.68-1.58 (m, 80H), 1.31-1.06 (m, 360H), 1.00-0.97 (m, 60H), 0.67-0.59 (m, 60H), -3.04(s, 4H), -3.35(s, 6H). ESI-MS (*m/z*): 1516.28 [M-22 PF_6^-]$^{22+}$ (calcd. *m/z* = 1516.13), 1595.35 [M-21 PF_6^-]$^{21+}$ (calcd. *m/z* = 1595.23), 1682.47 [M-20 PF_6^-]$^{20+}$ (calcd. *m/z* = 1682.24), 1778.69 [M-19 PF_6^-]$^{19+}$ (calcd. *m/z* = 1778.41), 1885.41 [M-18 PF_6^-]$^{18+}$ (calcd. *m/z* = 1885.27), 2004.79 [M-17 PF_6^-]$^{17+}$ (calcd. *m/z* = 2004.69), 2139.33 [M-16 PF_6^-]$^{16+}$ (calcd. *m/z* = 2139.65), 2291.37 [M-15 PF_6^-]$^{15+}$ (calcd. *m/z* = 2291.32), 2465.53 [M-14 PF_6^-]$^{14+}$ (calcd. *m/z* = 2465.34), 2666.41 [M-13 PF_6^-]$^{13+}$ (calcd. *m/z* = 2666.14), 2900.82 [M-12 PF_6^-]$^{12+}$ (calcd. *m/z* = 2900.40).

5. NMR, MS, TWIM-MS spectra, AFM and TEM of G0-G4.

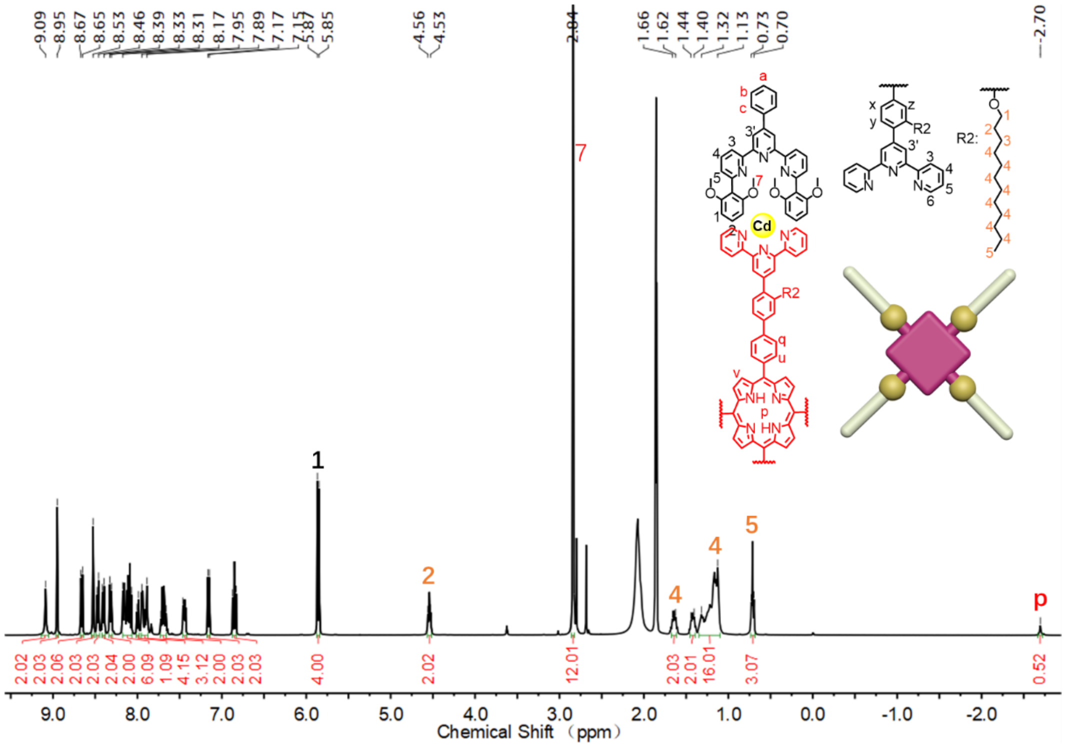

Figure S58. ^{1}H NMR spectrum of compound **G0** in CD_3CN.

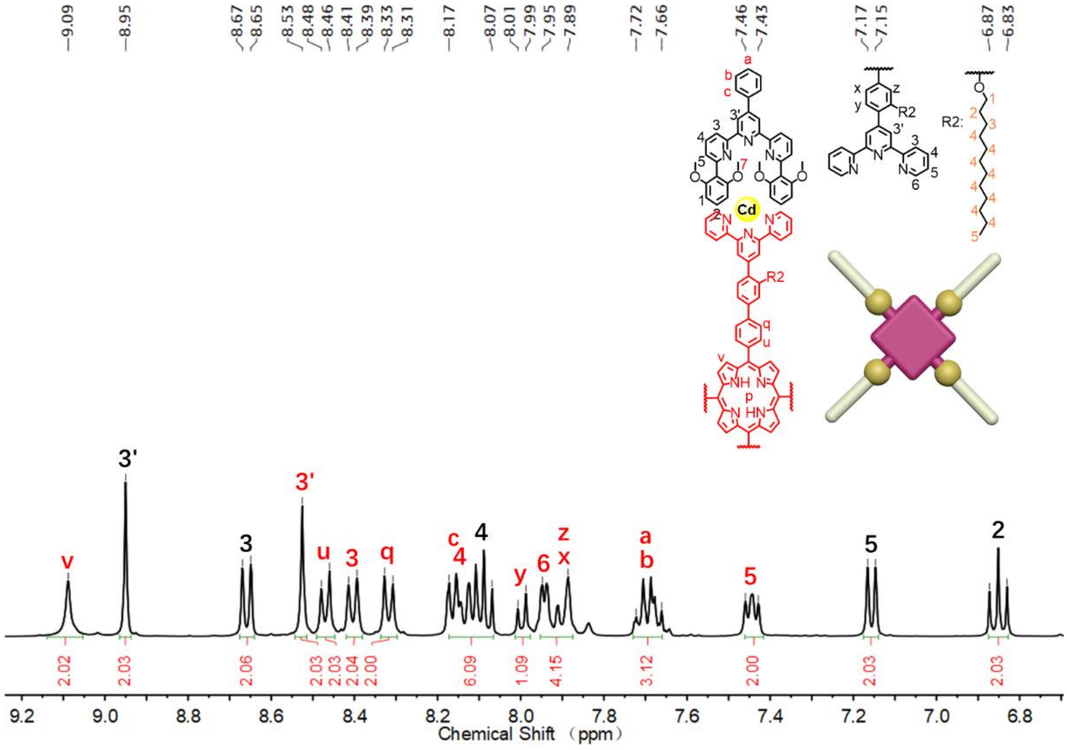

Figure S59. ^{1}H NMR spectrum of compound **G0** in CD_3CN.

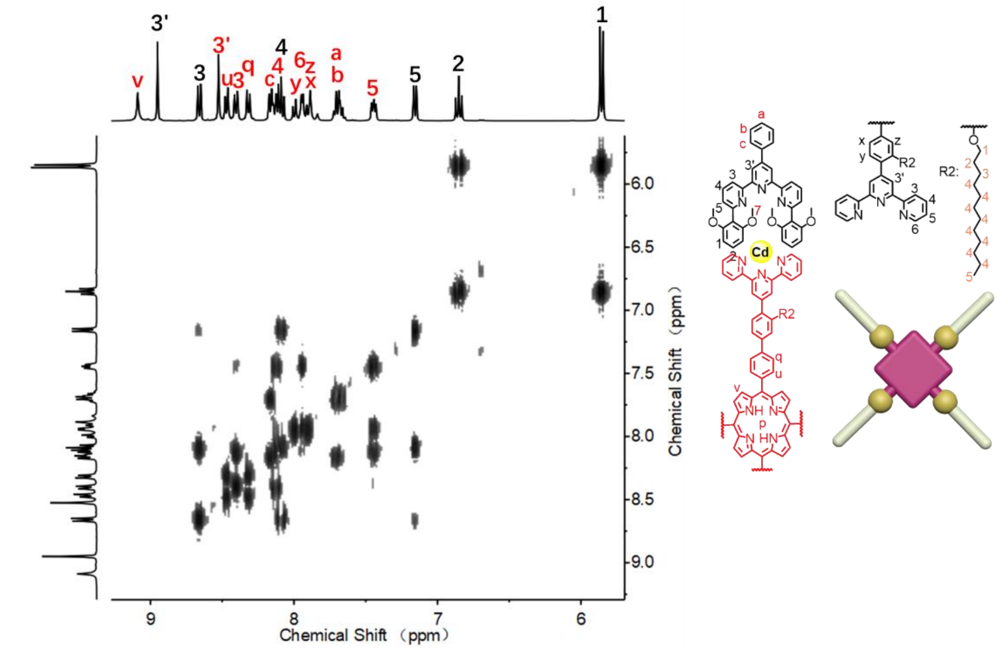

Figure S60. COSY spectrum of compound **G0** in CD_3CN.

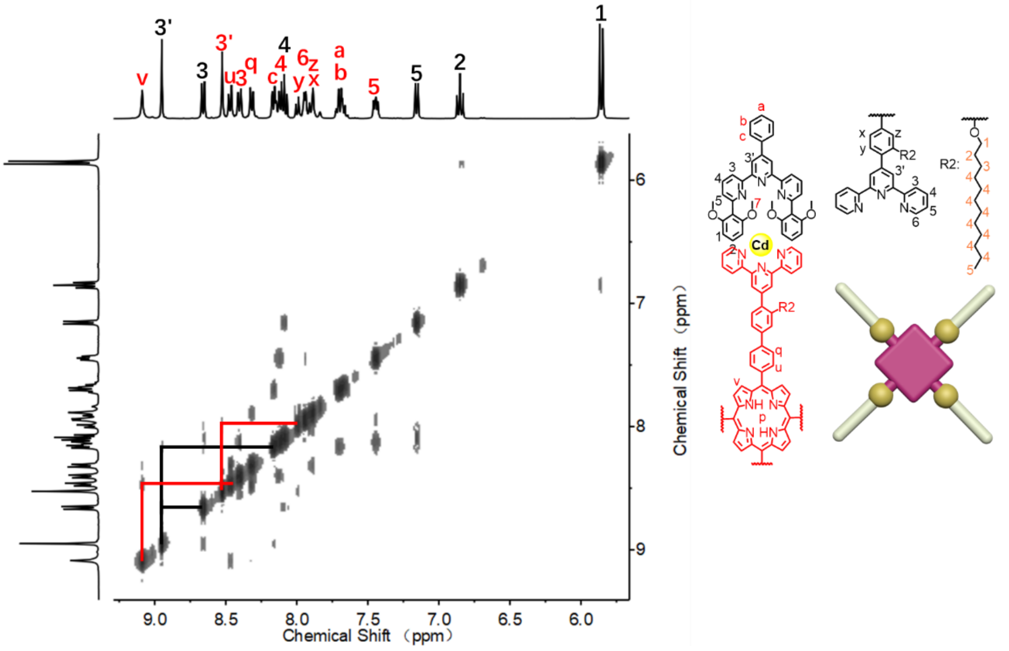

Figure S61. NOESY spectrum of compound **G0** in CD_3CN.

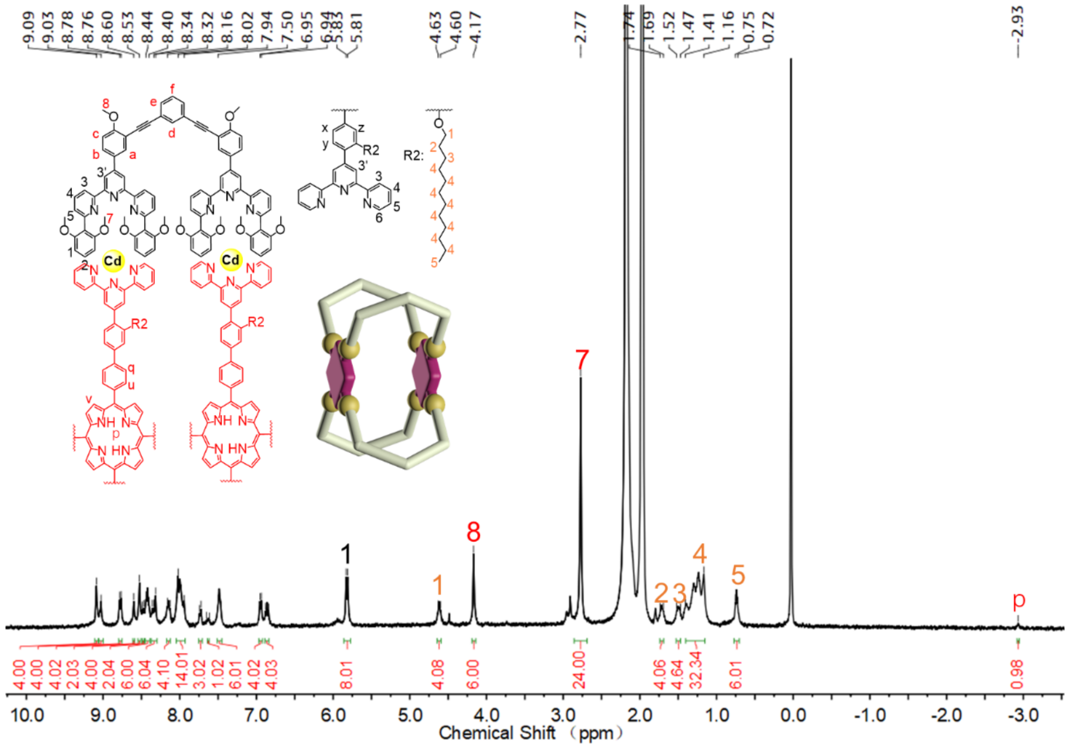

Figure S62. ^{1}H NMR spectrum of compound **G1** in CD_3CN.

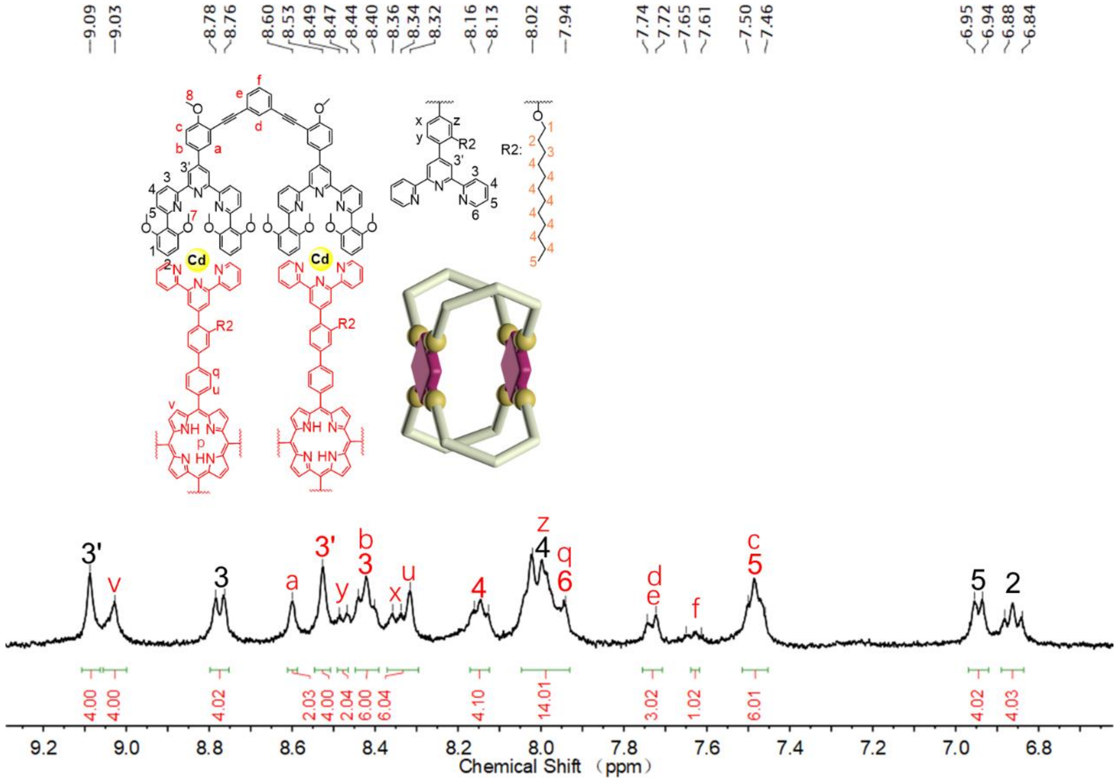

Figure S63. ^{1}H NMR spectrum of compound **G1** in CD_3CN.

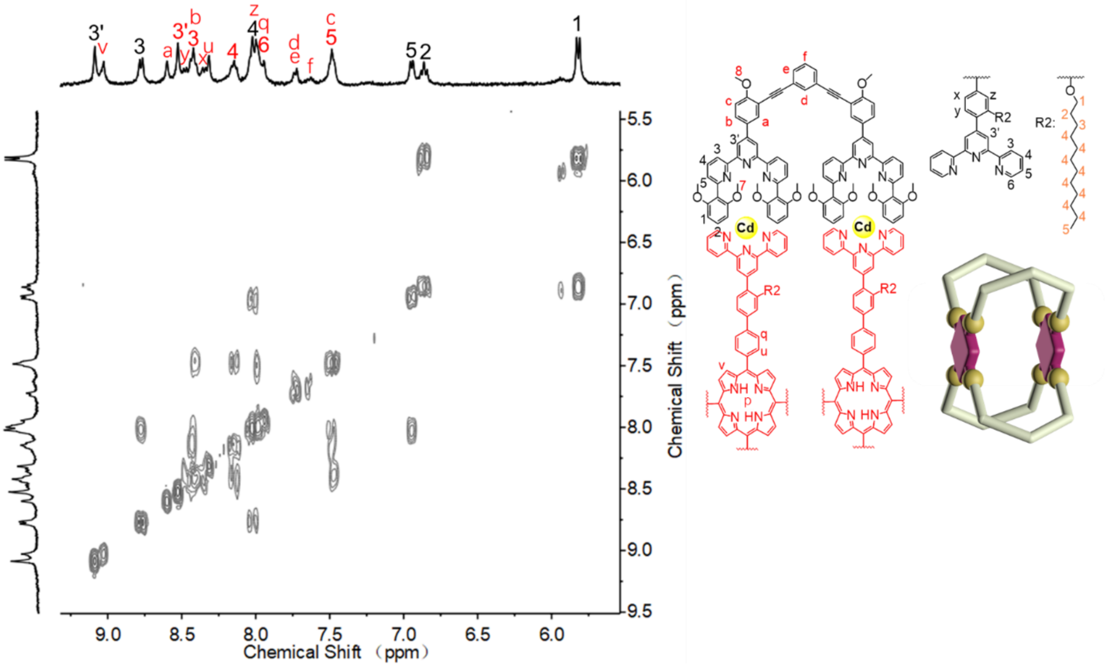

Figure S64. COSY spectrum of compound **G1** in CD_3CN.

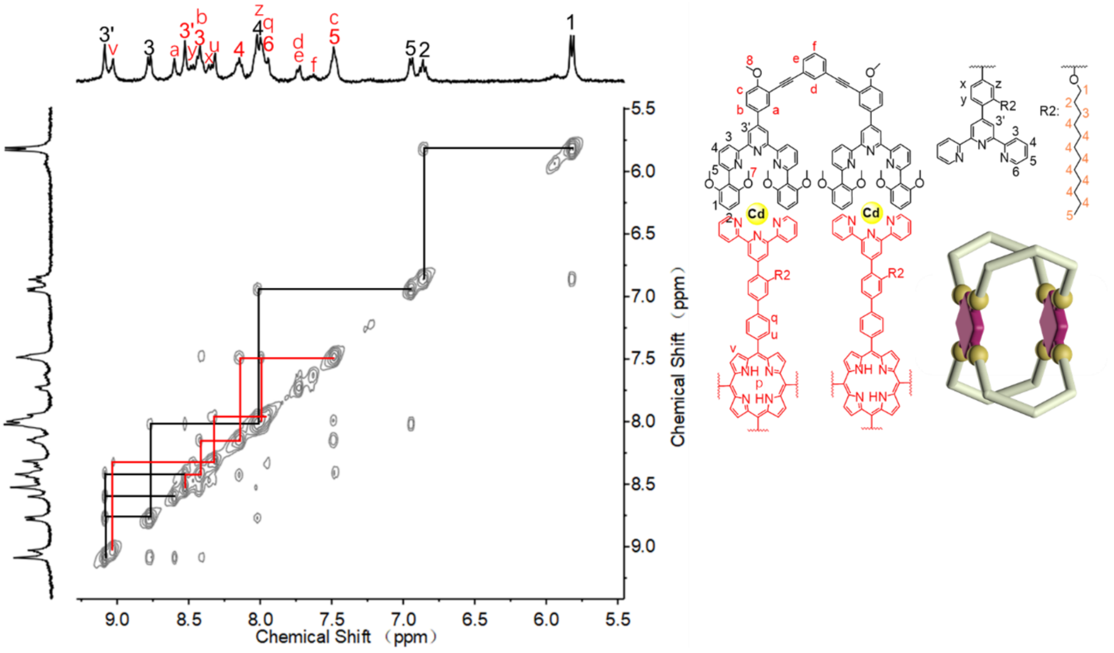

Figure S65. NOESY spectrum of compound **G1** in CD_3CN.

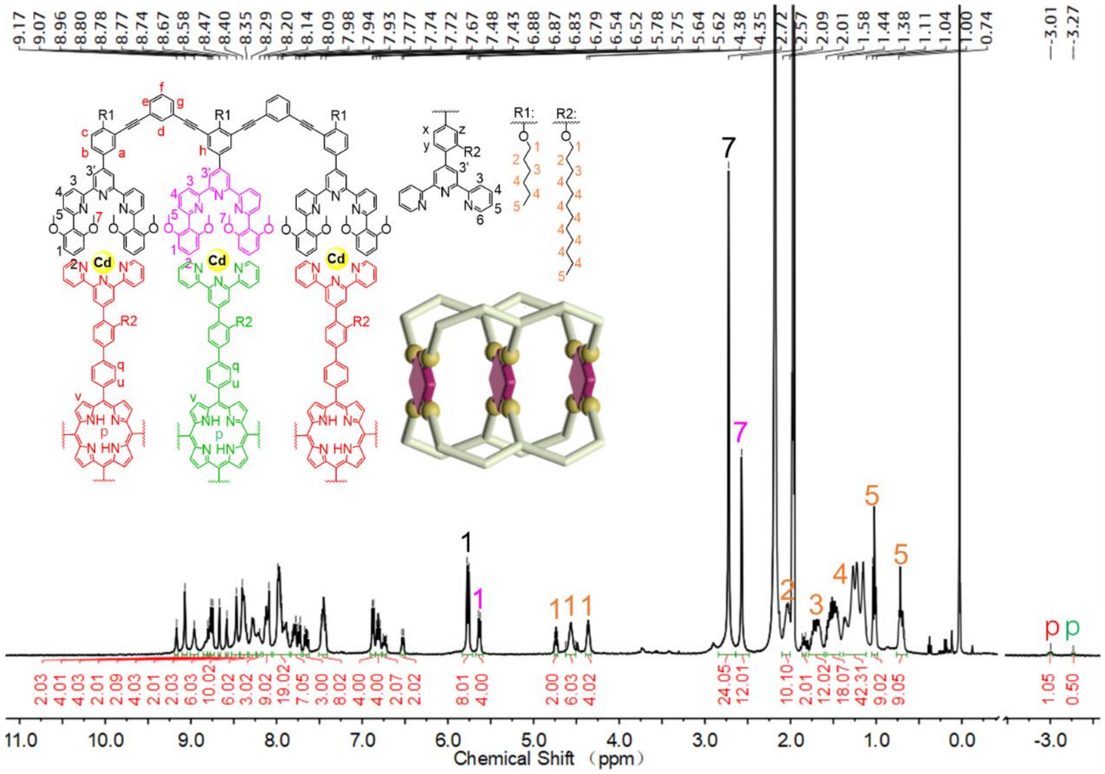

Figure S66. ^{1}H NMR spectrum of compound **G2** in CD_3CN.

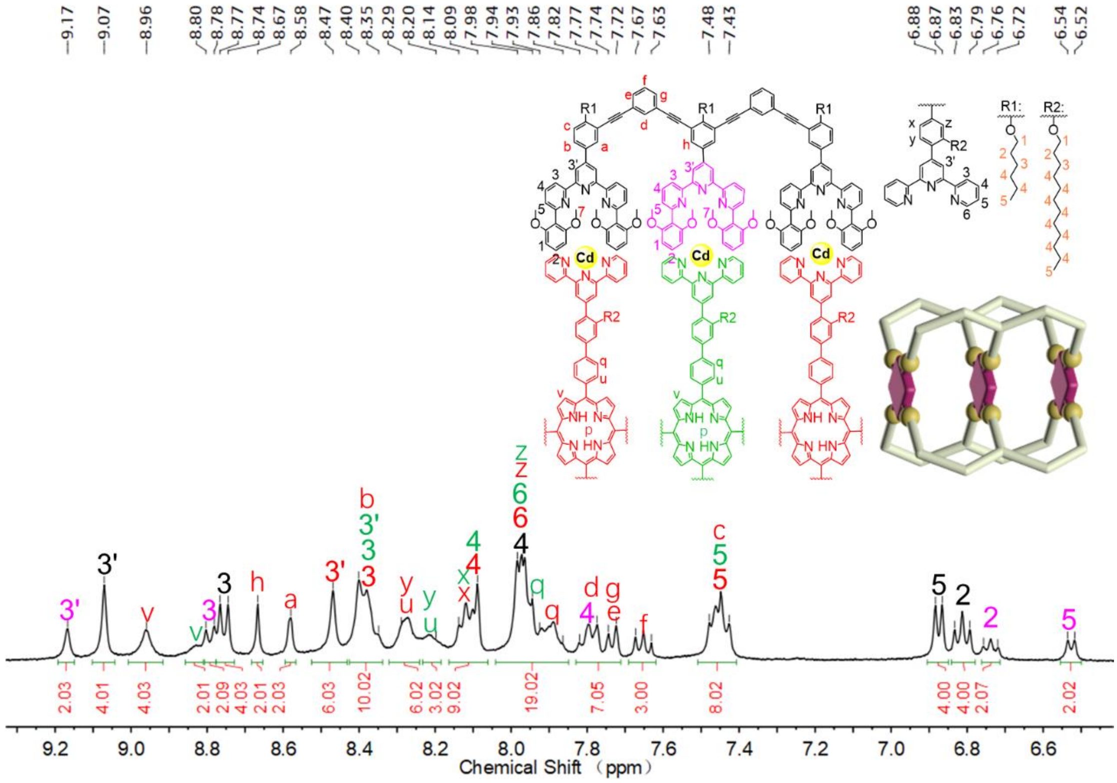

Figure S67. ^{1}H NMR spectrum of compound **G2** in CD_3CN.

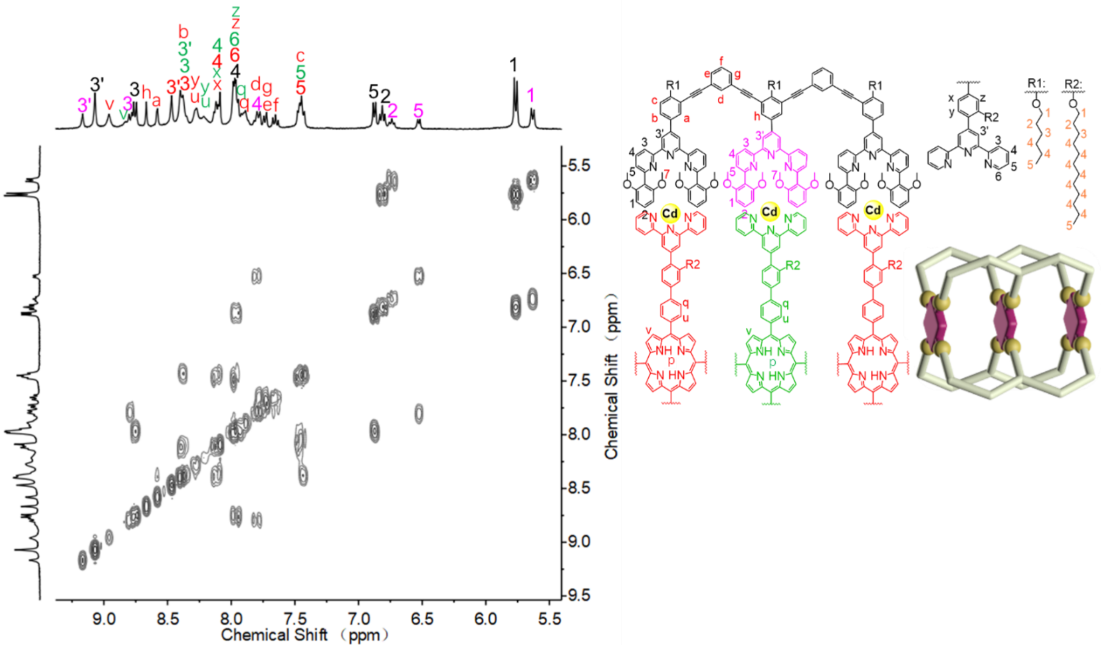

Figure S68. COSY spectrum of compound **G2** in CD_3CN.

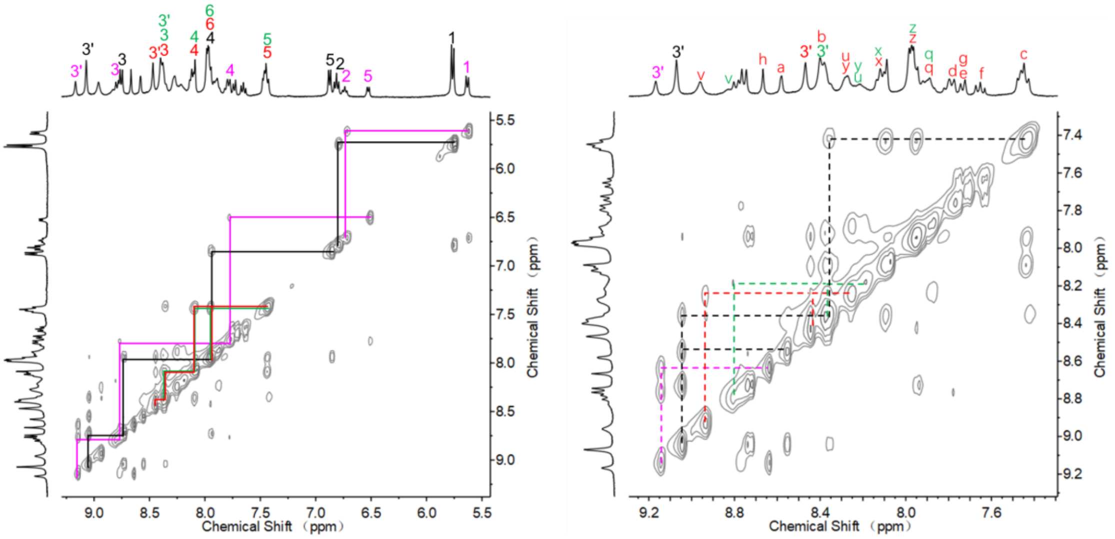

Figure S69. NOESY spectrum of compound **G2** in CD_3CN.

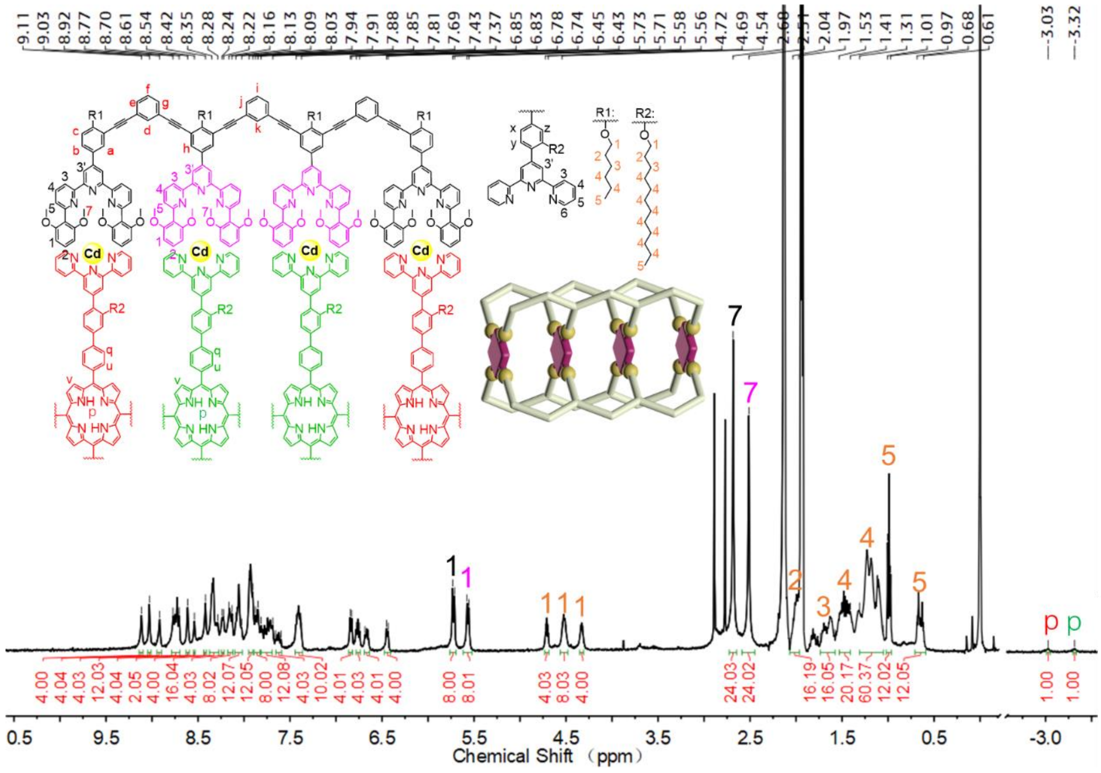

Figure S70. ^{1}H NMR spectrum of compound **G3** in CD_3CN.

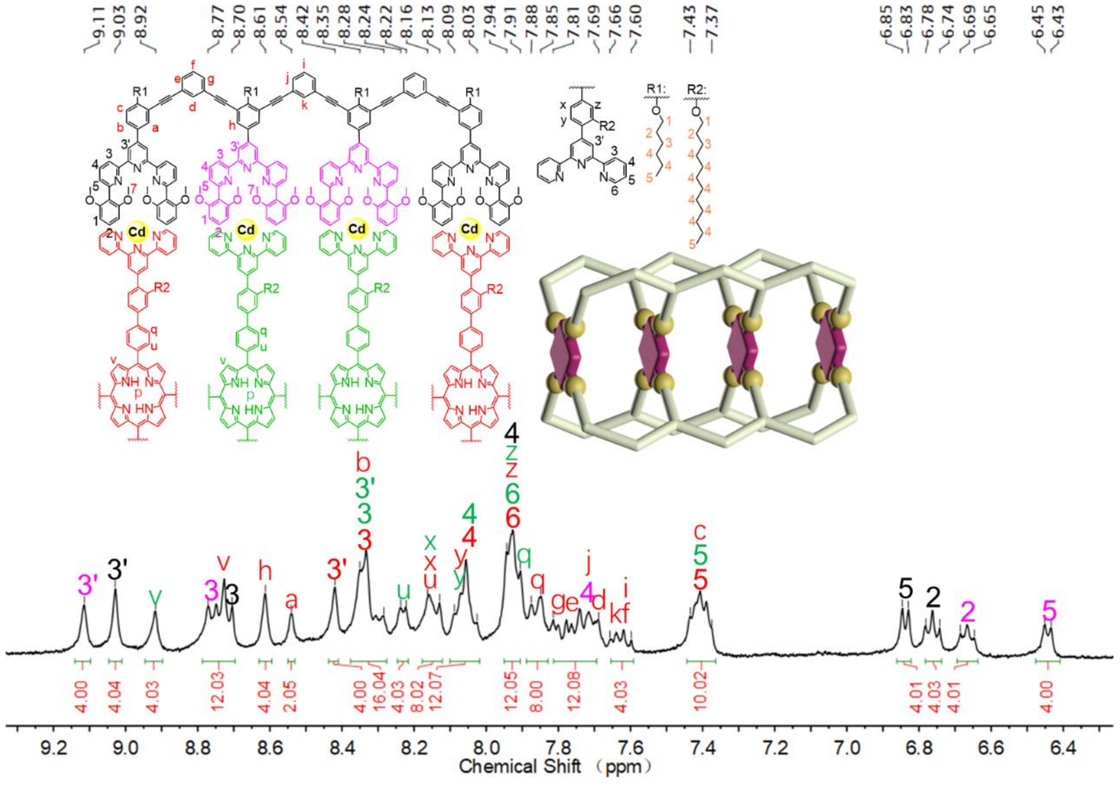

Figure S71. ^{1}H NMR spectrum of compound **G3** in CD_3CN.

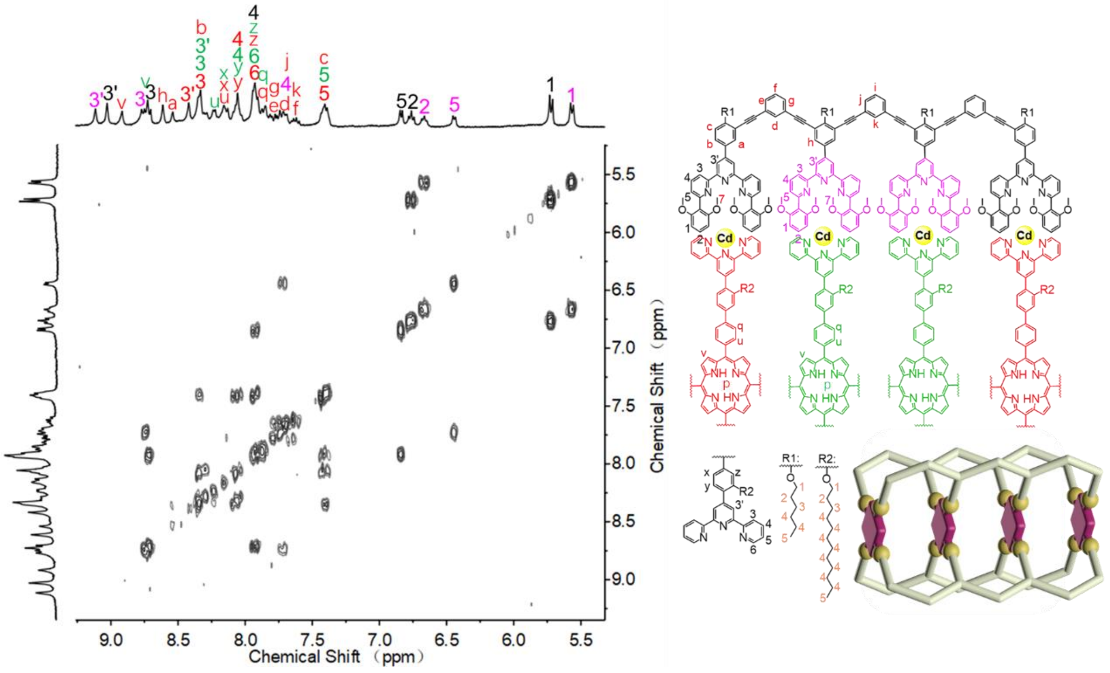

Figure S72. COSY spectrum of compound **G3** in CD_3CN.

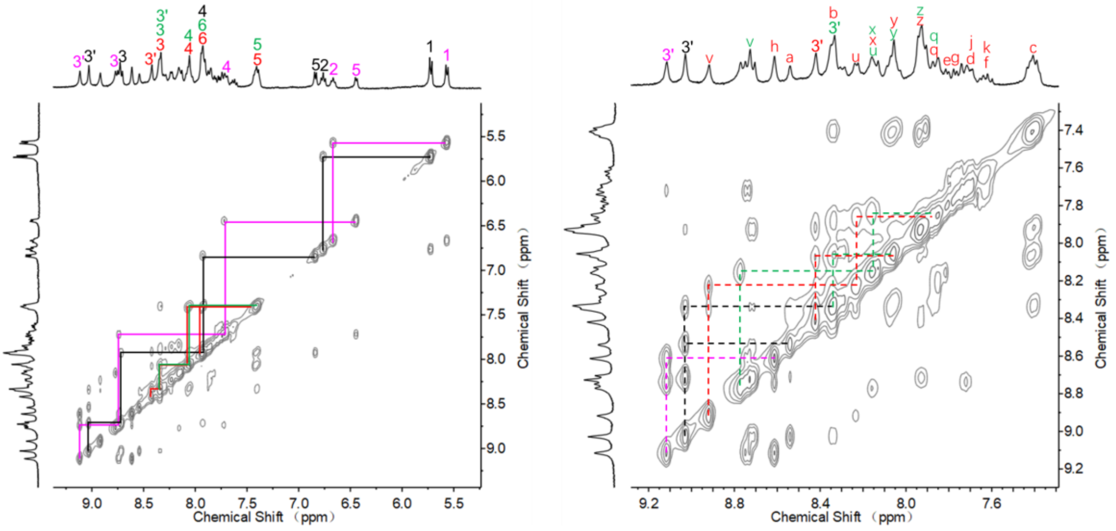

Figure S73. NOESY spectrum of compound **G3** in CD_3CN.

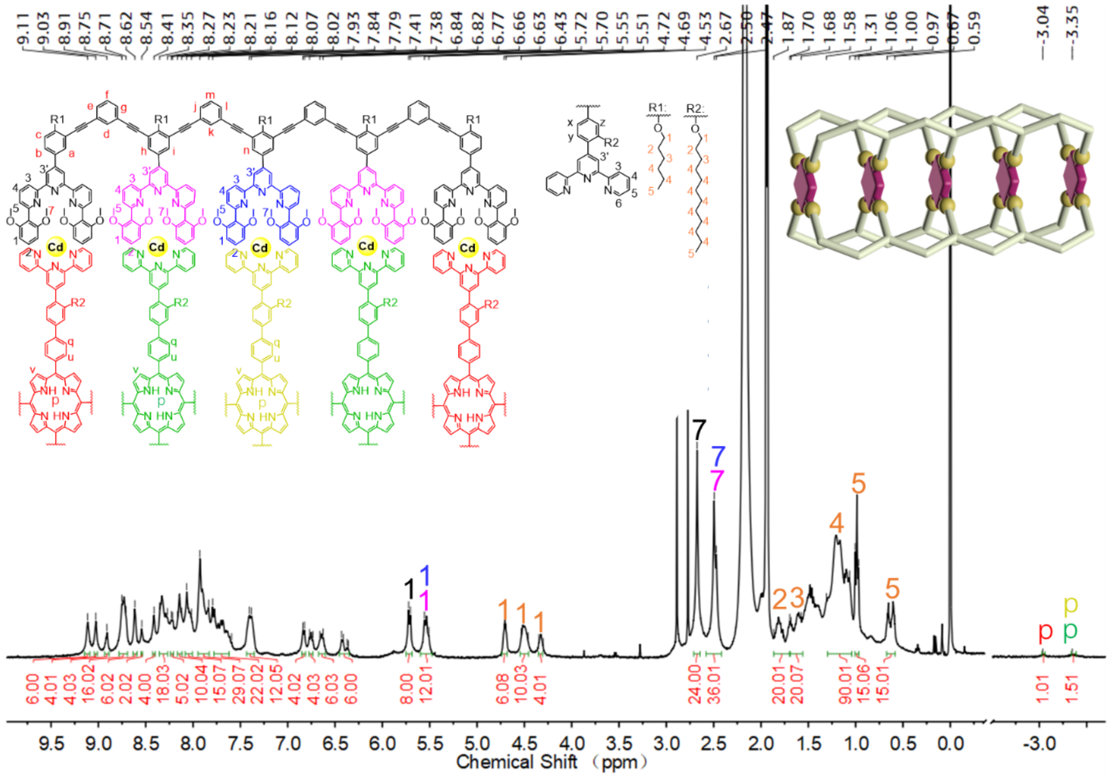

Figure S74. ^{1}H NMR spectrum of compound **G4** in CD_3CN.

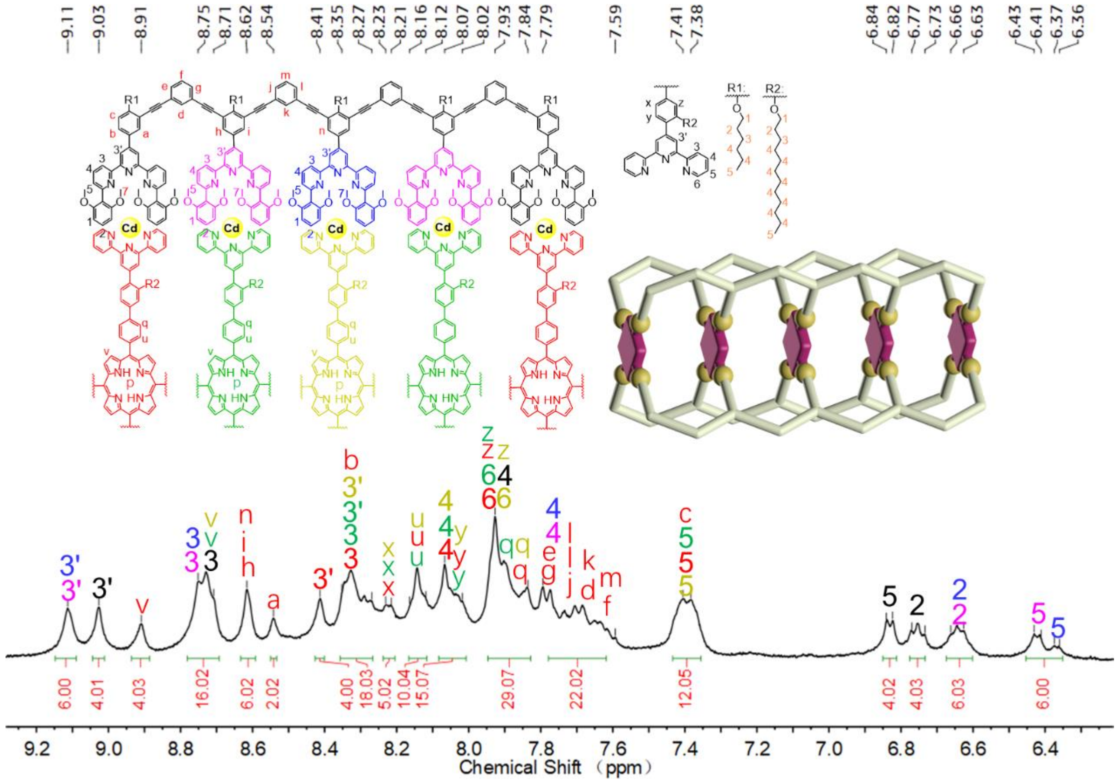

Figure S75. ^{1}H NMR spectrum of compound **G4** in CD_3CN.

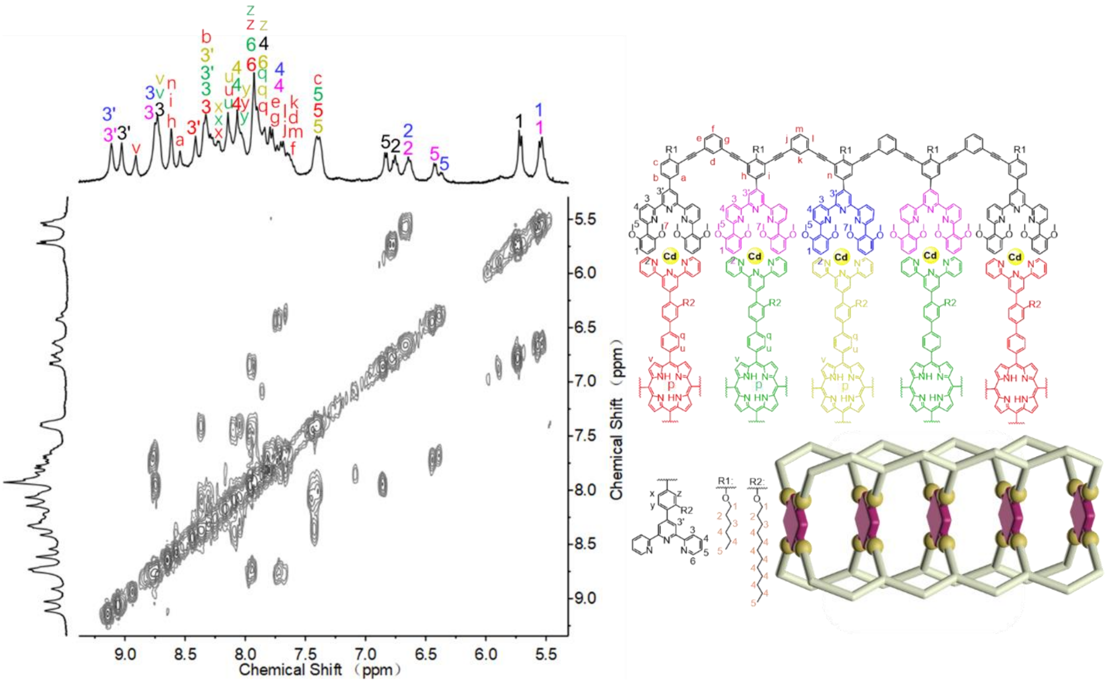

Figure S76. COSY spectrum of compound **G4** in CD_3CN.

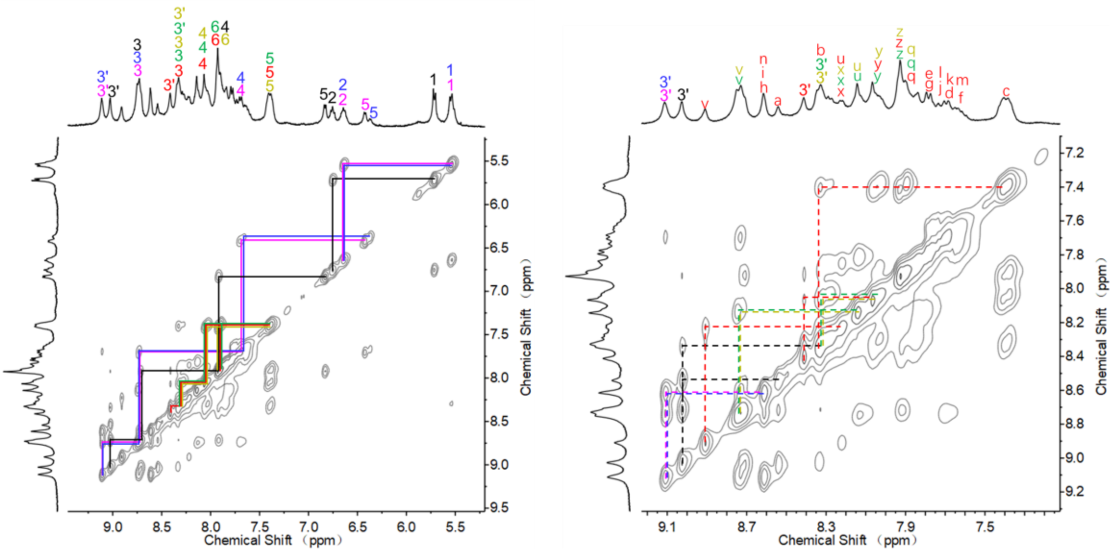

Figure S77. NOESY spectrum of compound **G4** in CD_3CN.

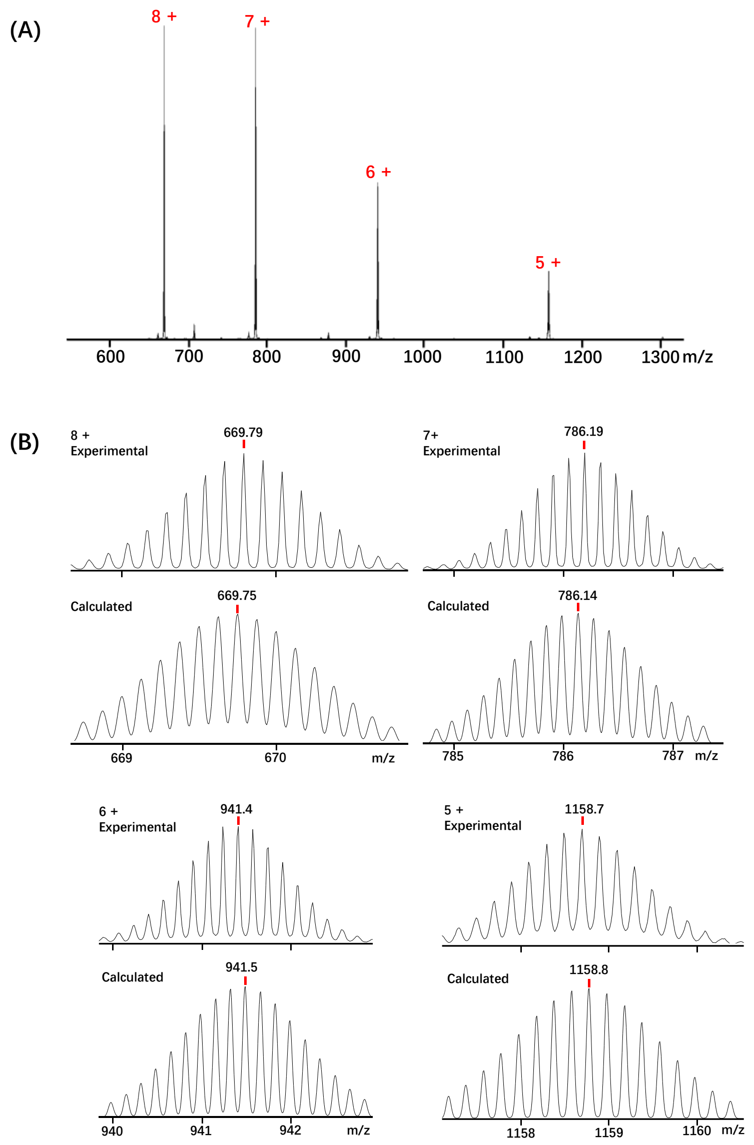

Figure S78: (A) ESI-MS spectrum of **G0**, (B) Calculated (bottom) and experimental (top) isotope patterns for the different charge states observed from **G0** (PF_6^- as counterion).

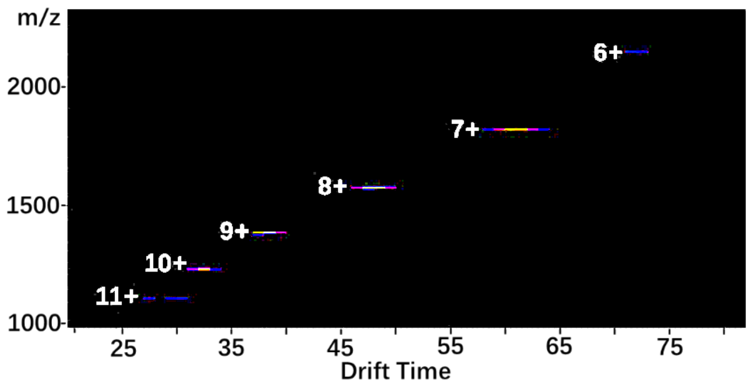

Figure S79. ESI-TWIM-MS plot of **G1**.

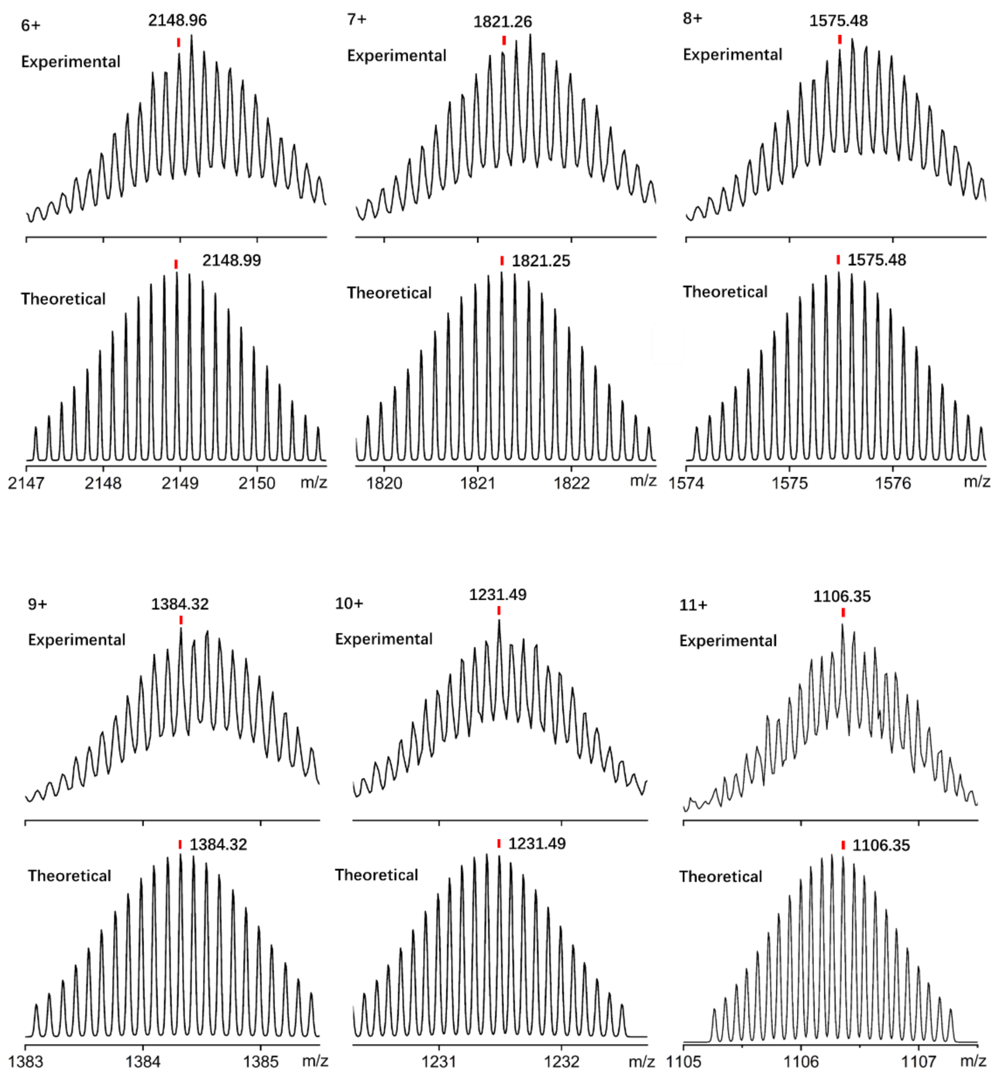

Figure S80. Theoretical (bottom) and experimental (top) isotope patterns for the different charge states observed from **G1**(PF_6^- as counterion).

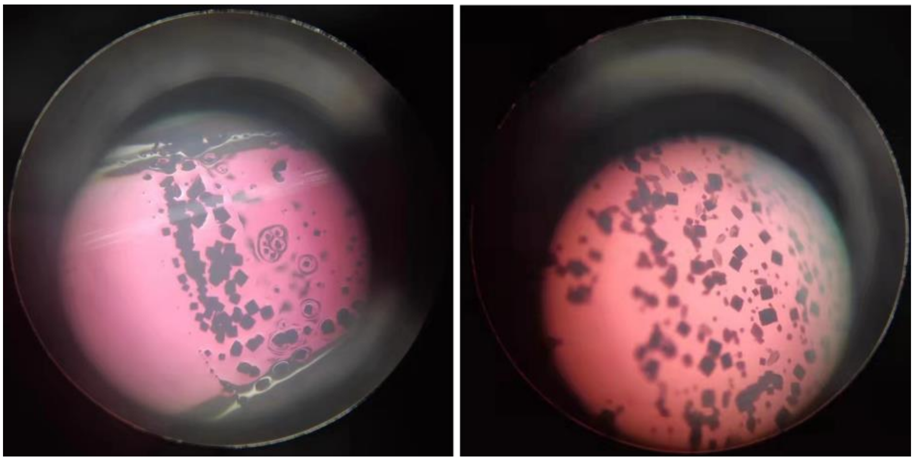

Figure S81. The image of **G1** and **C_{60}@G1** crystals grown under slow vapor diffusion (10 days) of ethyl acetate into a DMF solution.

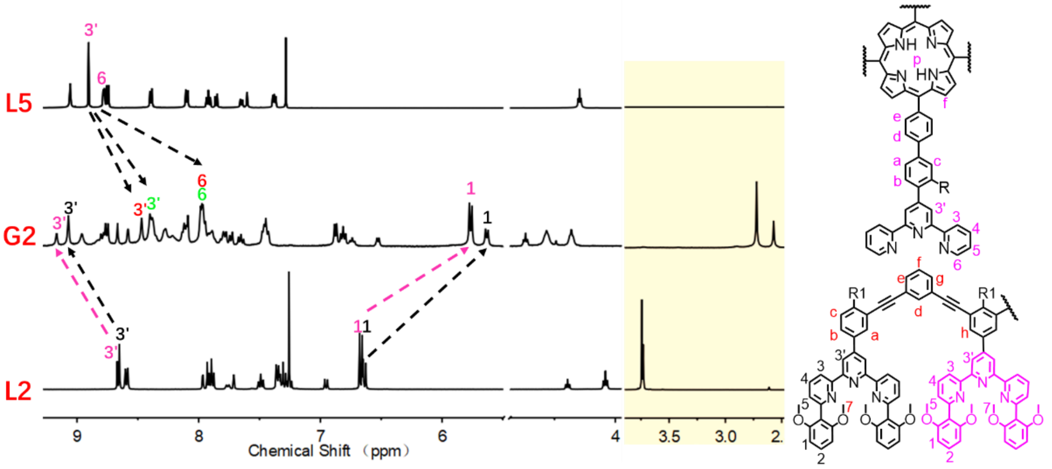

Figure S82. Comparison diagram of ^{1}H NMR spectra of **G2** and its module ligands **L2** and **L5**.

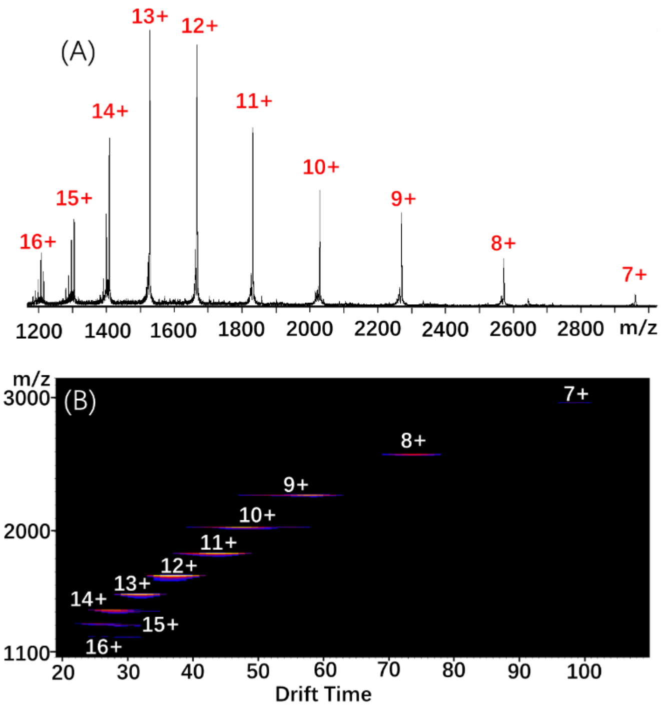

Figure S83. ESI-MS spectrum and ESI-TWIM-MS plot of **G2**.

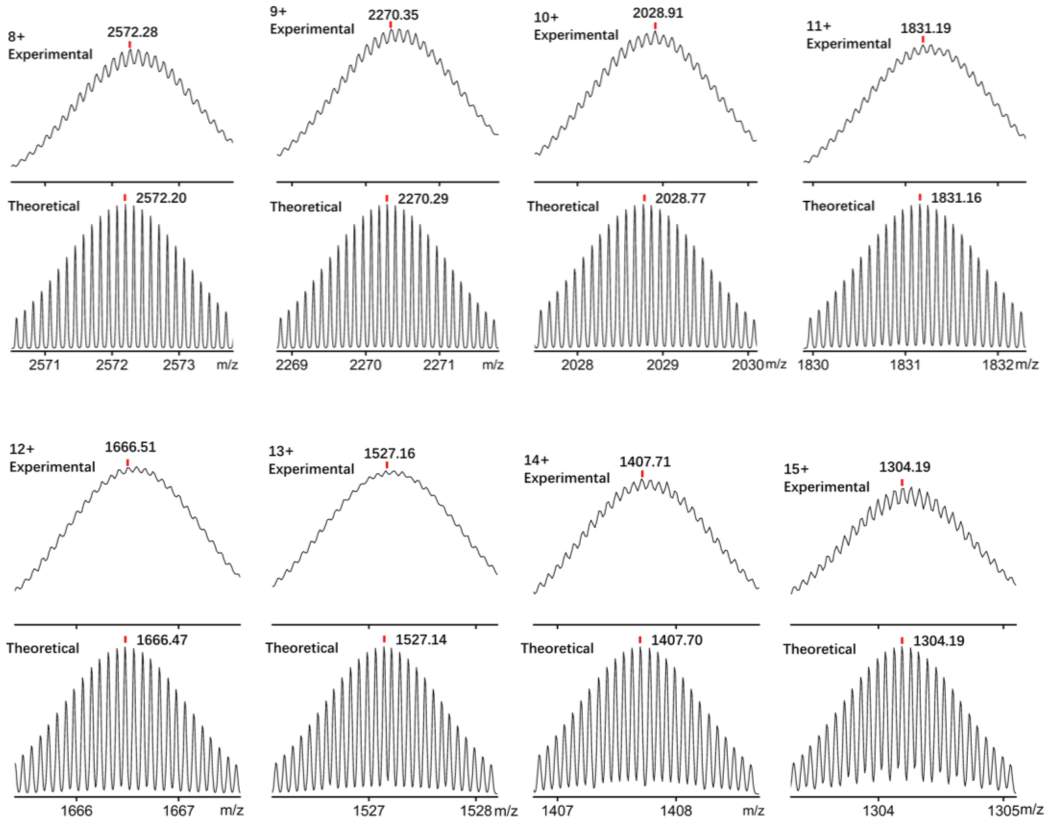

Figure S84: Theoretical (bottom) and experimental (top) isotope patterns for the different charge states observed from **G2**(PF_6^- as counterion).

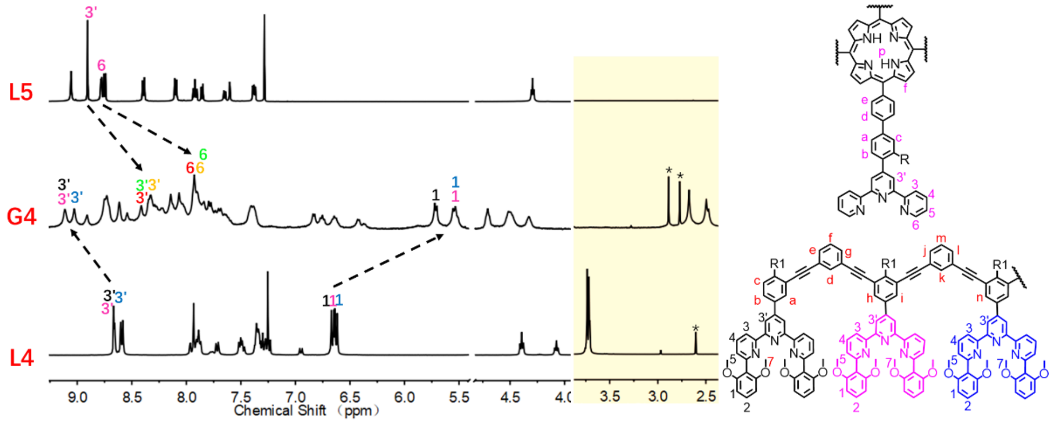

Figure S85. Comparison diagram of ^{1}H NMR spectra of **G4** and its module ligands **L4** and **L5**.

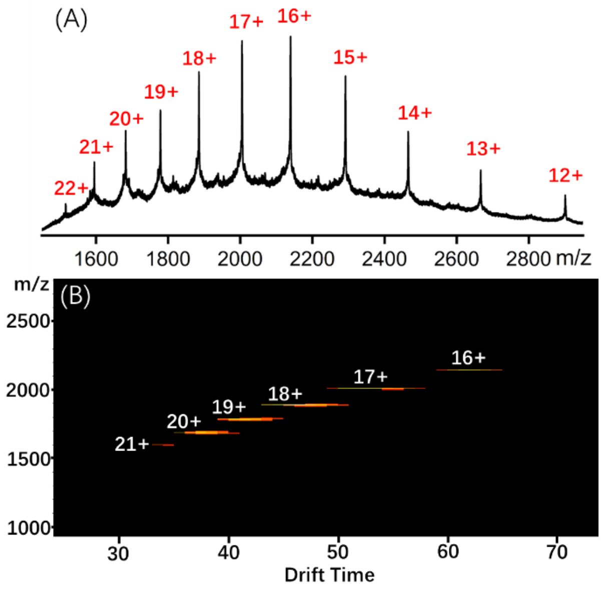

Figure S86. ESI-MS spectrum and ESI-TWIM-MS plot of **G4**.

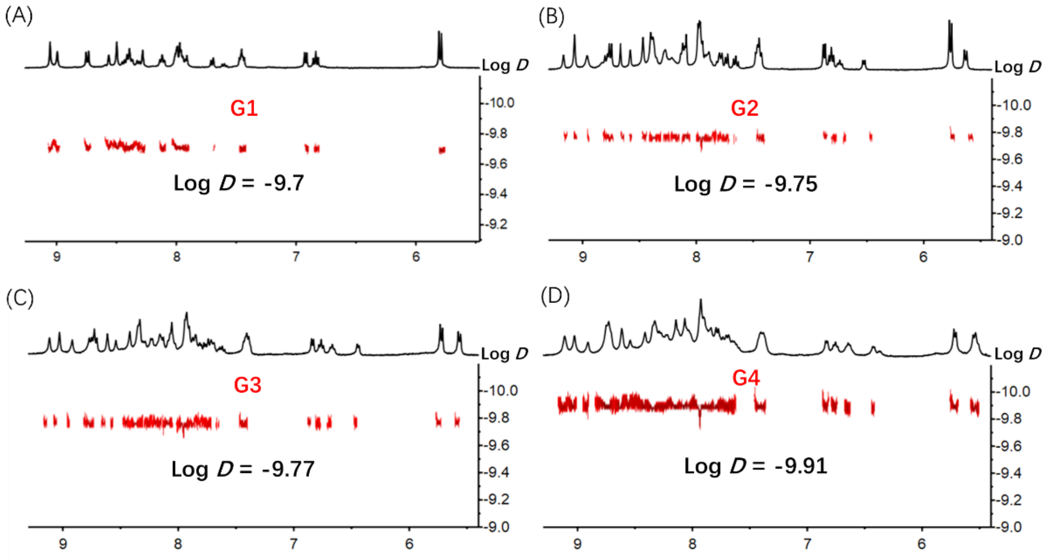

Figure S87. DOSY spectra (500 MHz, 298K) of **G1-G4** in CD_3CN.

The hydrodynamic radius of **G1-G4** can be estimated, according to the Stokes-Einstein Equation. Where D is the diffusion constant, k is the Boltzmann's constant, T is the temperature, μ is the viscosity of solvents, and R is the radius:

$$D = kT/6\pi\mu R$$

$$D\,(G1) = 10^{-9.7}\ m^2\ s^{-1},\ D\,(G2) = 10^{-9.75}\ m^2\ s^{-1},\ D\,(G3) = 10^{-9.77}\ m^2\ s^{-1},\ D\,(G4) = 10^{-9.91}\ m^2\ s^{-1}$$

$$k = 1.38\times10^{-23}\ N\ m\ K^{-1},$$

$$T = 298\ K,$$

$$\mu = 3.67\times10^{-4}\ N\ m^{-2}\ s\ (CD_3CN,\ 298\ K),$$

R(**G1**) = 3.0 nm, R(**G2**) = 3.3 nm, R(**G3**) = 3.5 nm, R(**G4**) = 4.8 nm.

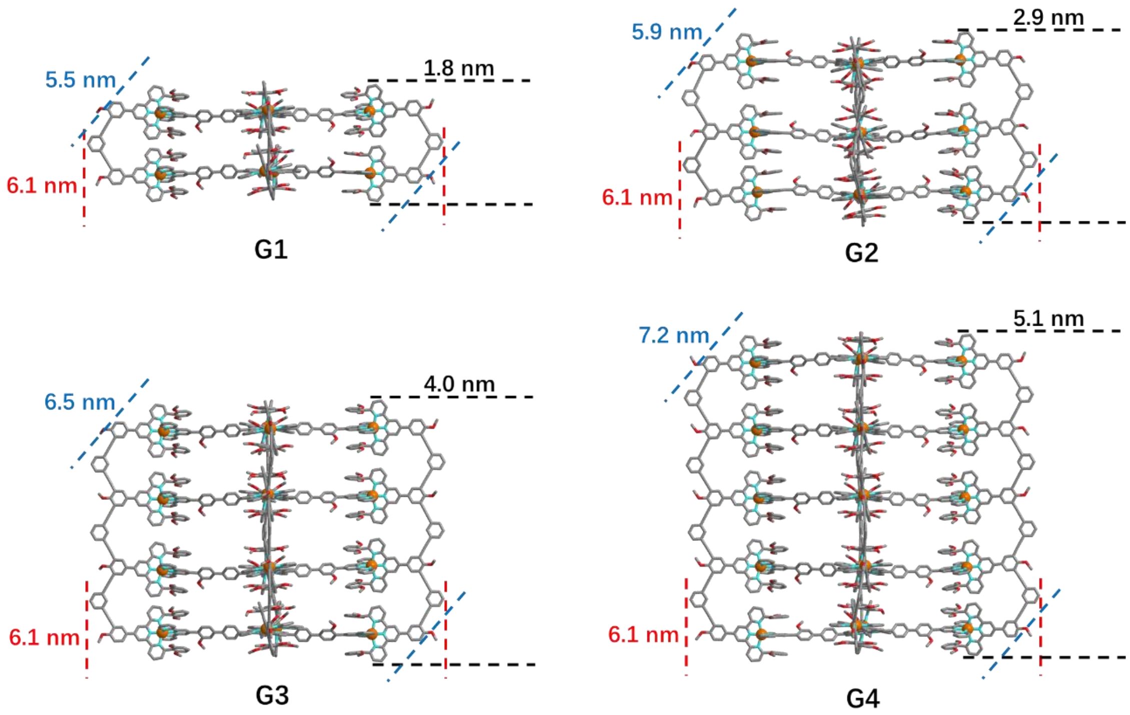

Figure S88. The molecular energy minimization diagram of **G1-G4** and the sizes of three different directions (Obtained through Materials Studio).

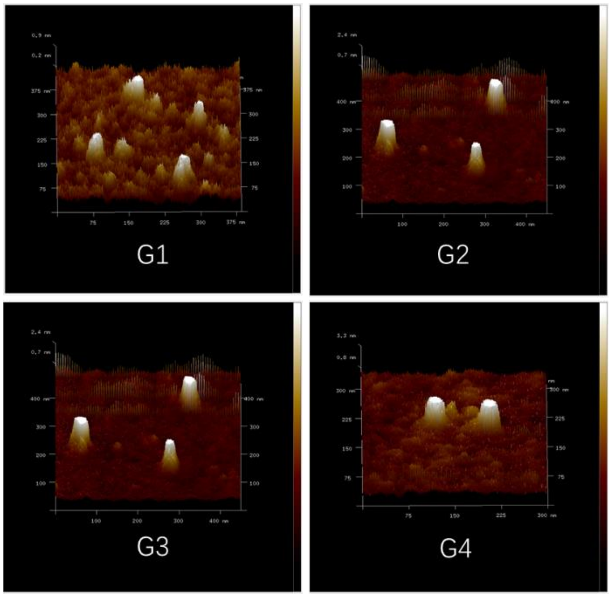

Figure S89. 3D AFM micrograph of **G1-G4**.

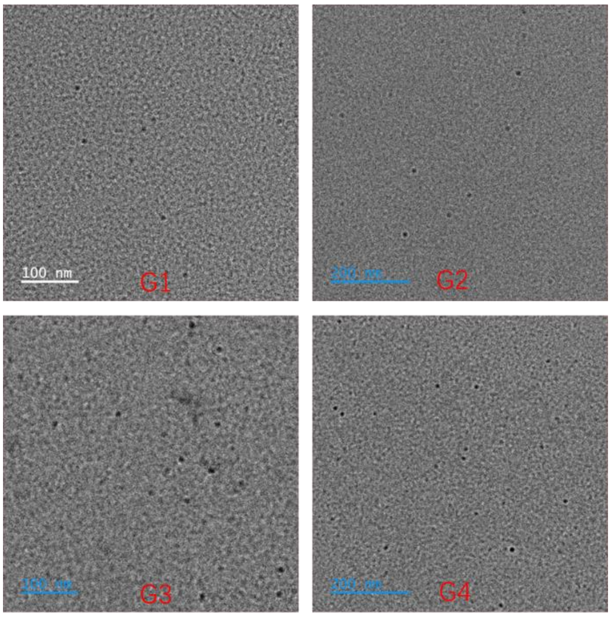

Figure S90. TEM micrograph of **G1-G4**.

6. The host-guest interaction between G0-G4 and C_{60}.

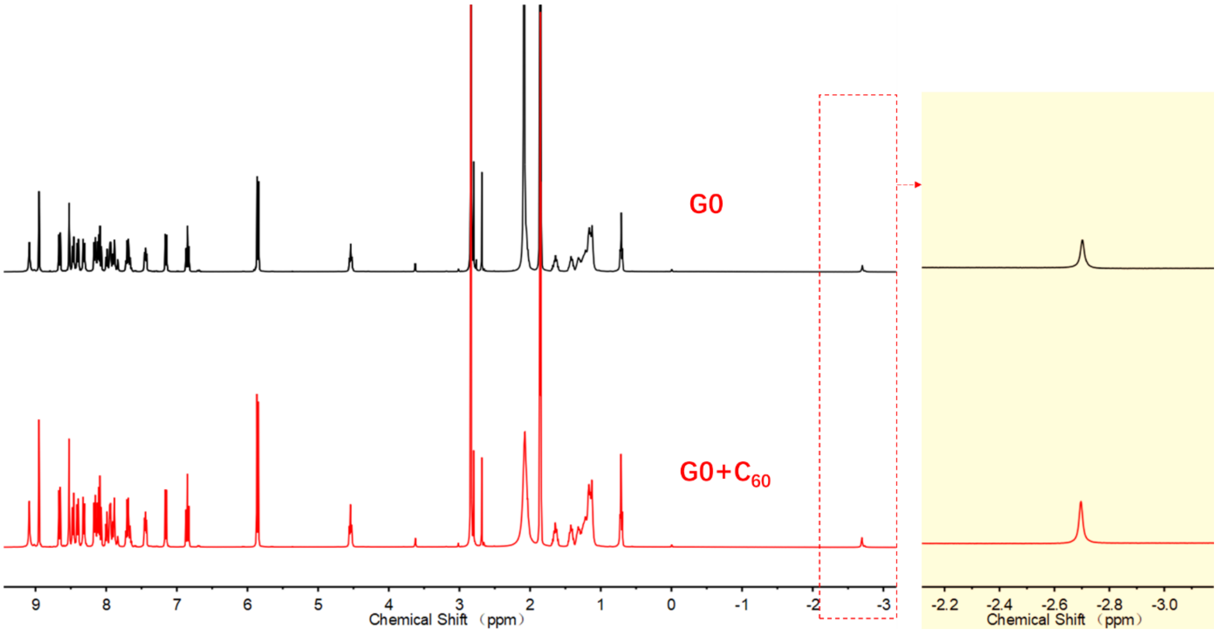

Figure S91. Comparison diagram of ^{1}H NMR spectra (600 M) of **G0** and **G0**+C_{60} (the pink part was enlarged).

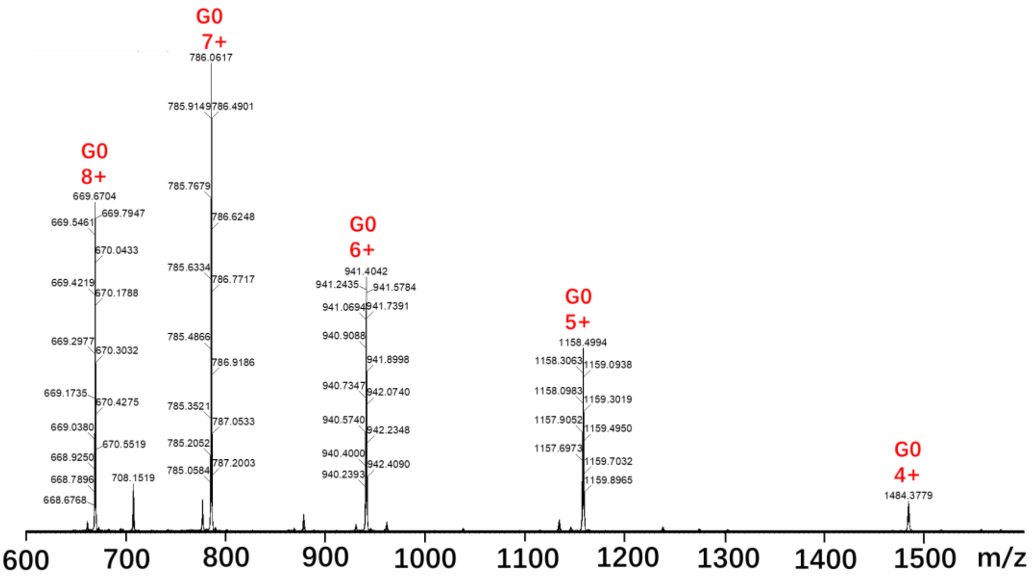

Figure S92: ESI-MS spectrum of **G0**+C_{60}.

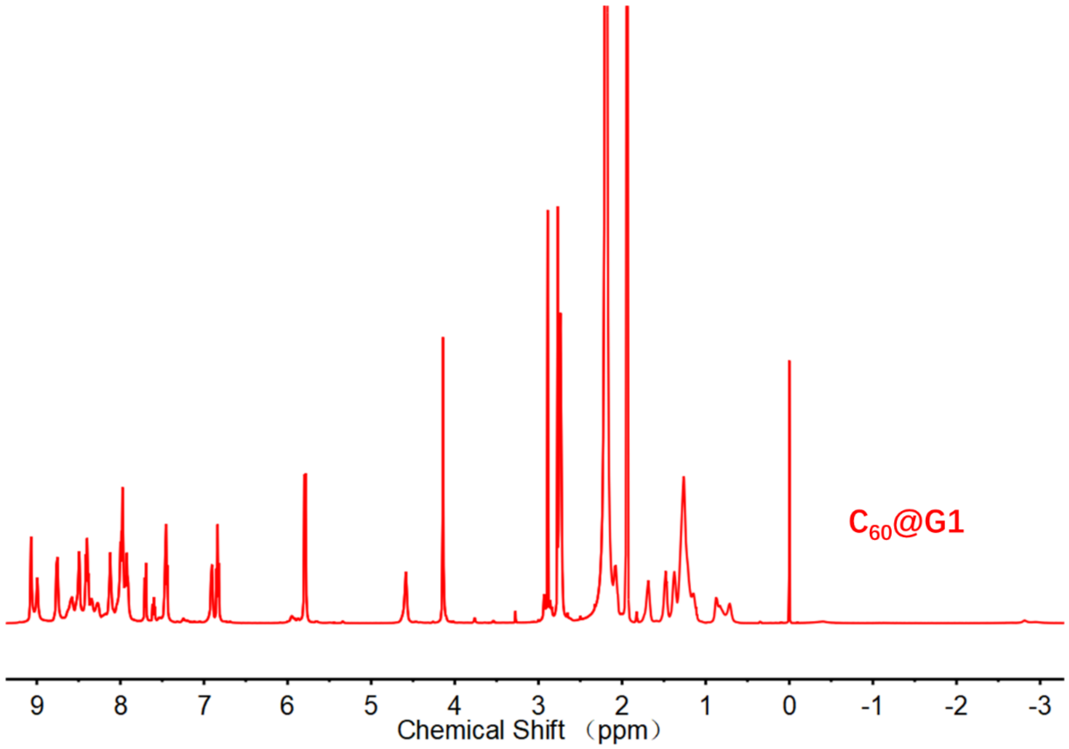

Figure S93. ^{1}H NMR spectrum of compound **C_{60}@G1** in CD_3CN.

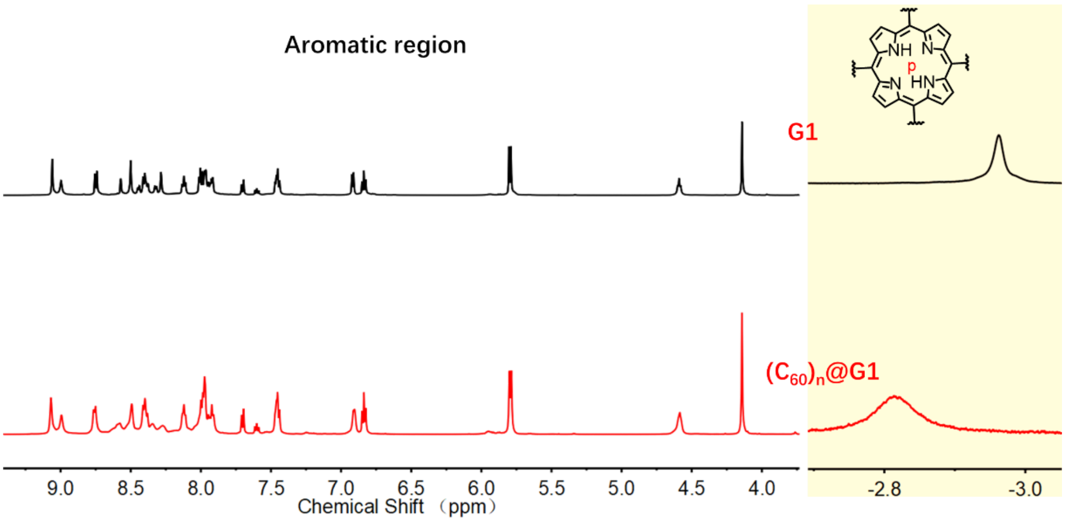

Figure S94. Comparison diagram of ^{1}H NMR spectra (600 M) of **G1** and **C_{60}@G1** (the pink part was enlarged).

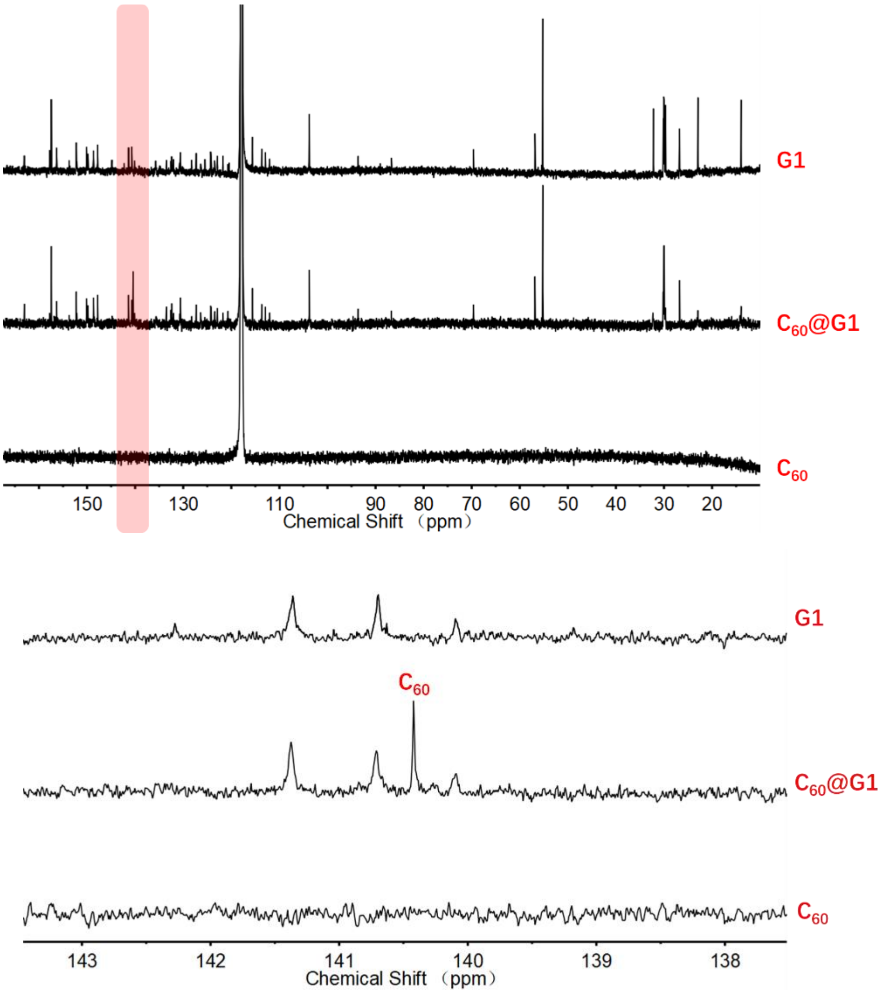

Figure S95. Comparison diagram of ^{13}C NMR spectra (600 M) of **G1**, C_{60}**@G1** and C_{60} in CD_3CN.

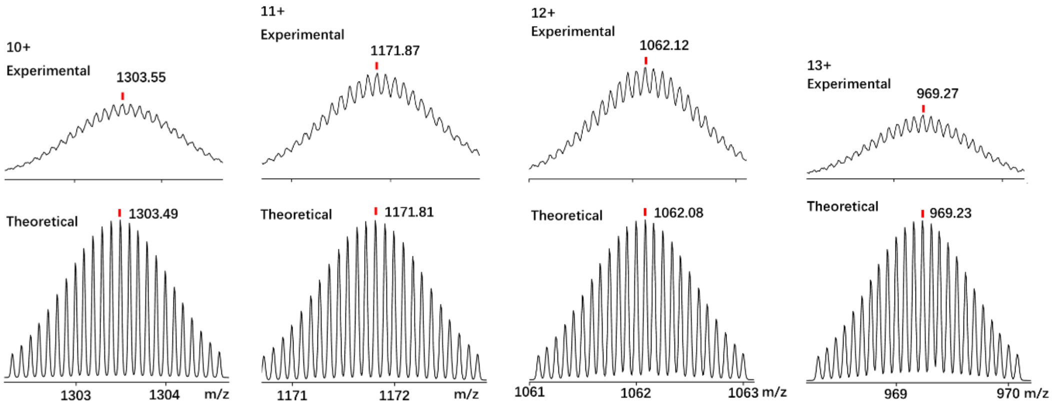

Figure S96. Theoretical (bottom) and experimental (top) isotope patterns for the different charge states observed from **C_{60}@G1**(PF_6^- as counterion).

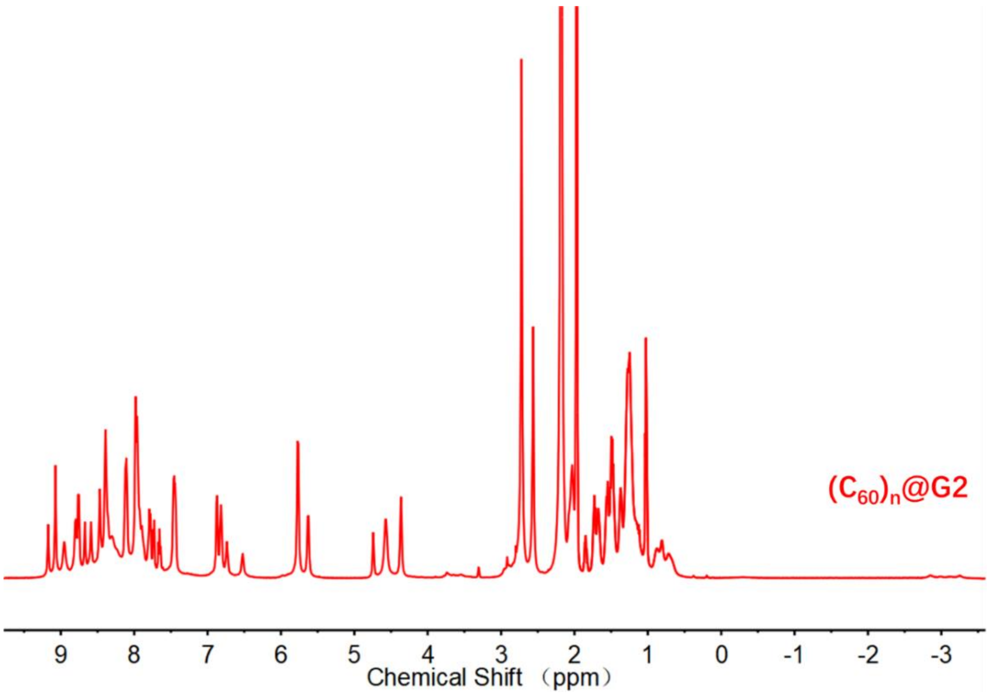

Figure S97. ^{1}H NMR spectrum of compound **$(C_{60})_n$@G2** in CD_3CN.

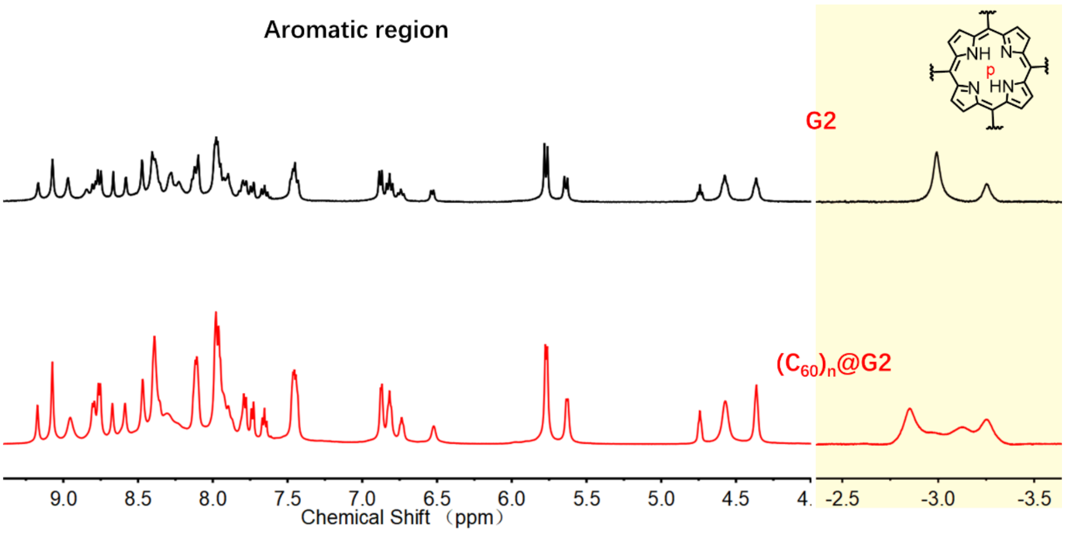

Figure S98. Comparison diagram of ^{1}H NMR spectra (600 M) of **G2** and **$(C_{60})_n$@G2** (the pink part was enlarged).

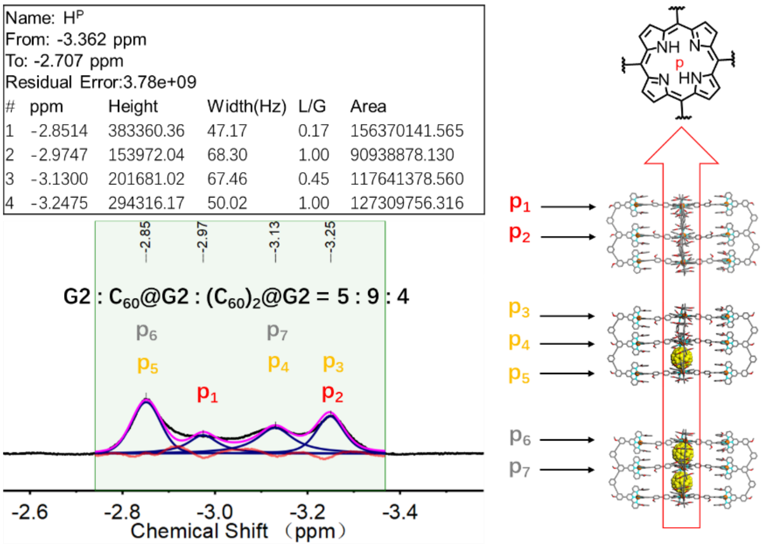

Figure S99. The ^{1}H NMR spectra integrations and attribution of H^P for $(C_{60})_n$@**G2** and proportion of **G2**, C_{60}@**G2** and $(C_{60})_2$@**G2**.

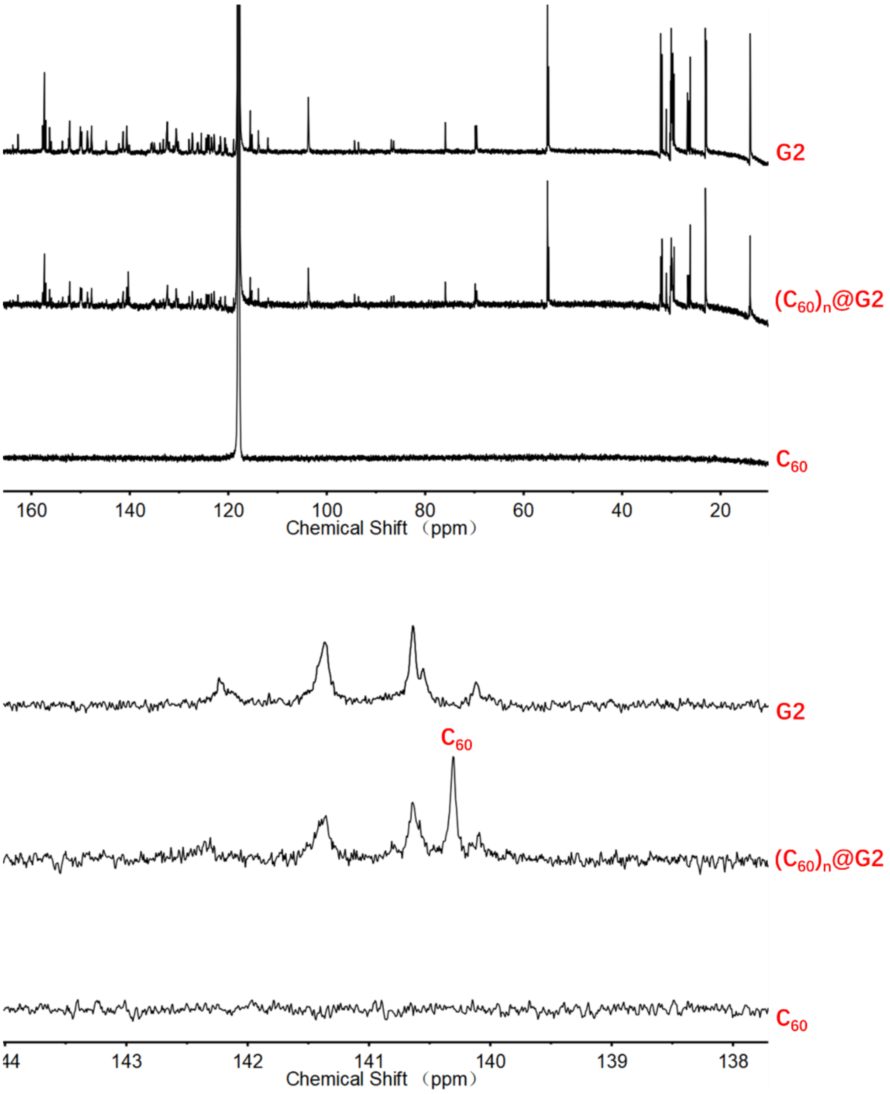

Figure S100. Comparison diagram of ^{13}C NMR spectra (600 M) of **G2**, $(C_{60})_n$**@G2** and C_{60} in CD_3CN.

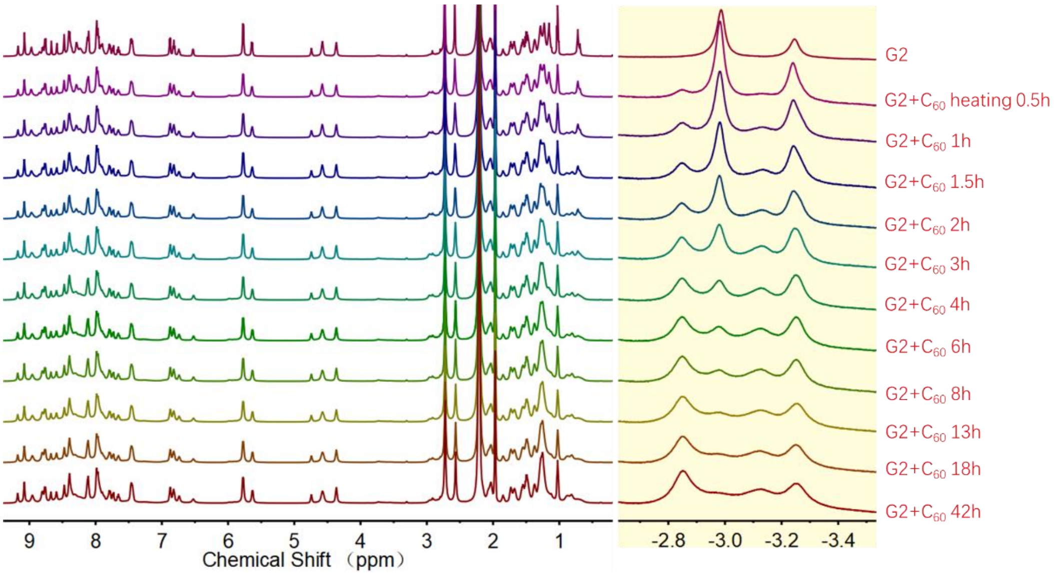

Figure S101. ^{1}H NMR spectra (600 M) monitoring of the process of **G2** wrapping C_{60} under 353K heating conditions (the pink part was enlarged).

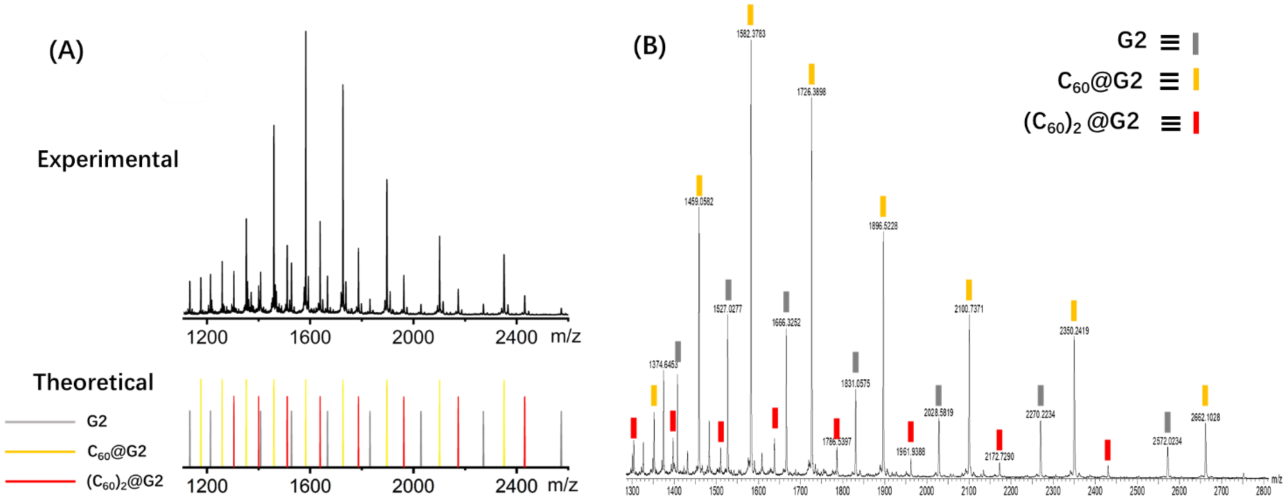

Figure S102. (A) Experimental and theoretical values of ESI-MS data in DMF for **G2**, C_{60}@**G2** and $(C_{60})_2$@**G2**; (B) ESI-MS spectrum of $(C_{60})_n$@**G2** in CH_3CN.

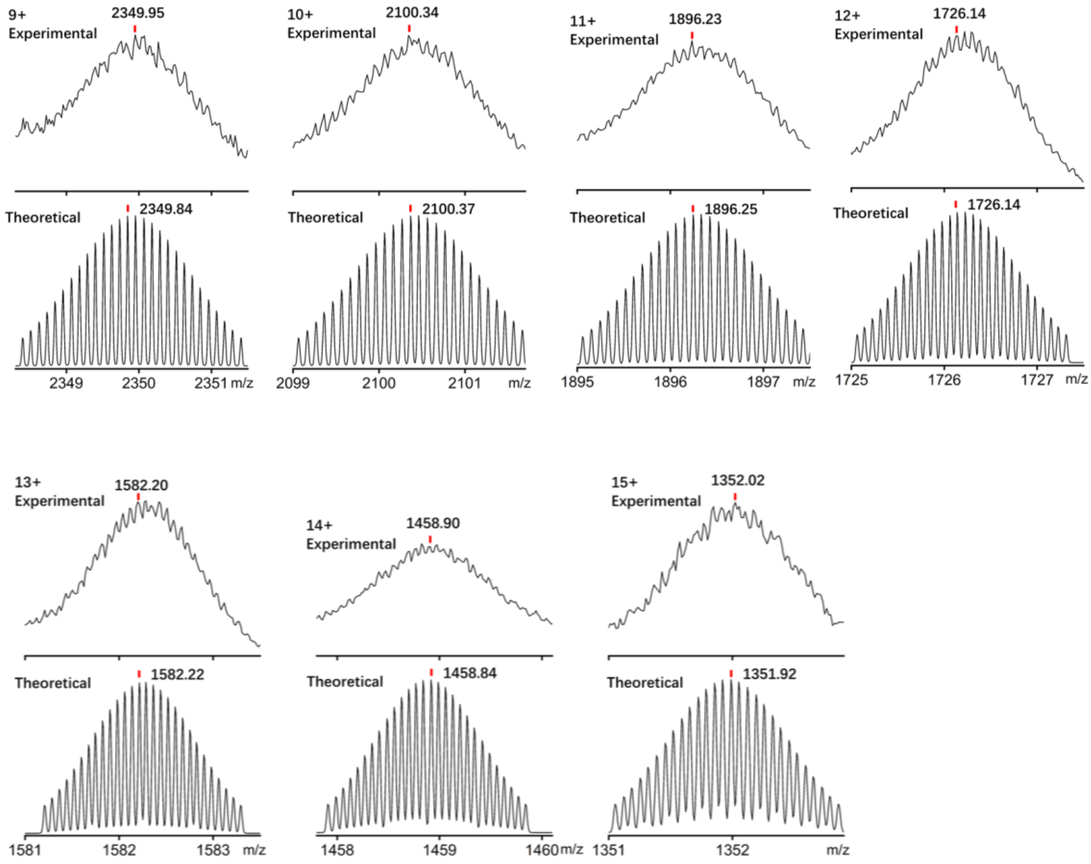

Figure S103. Theoretical (bottom) and experimental (top) isotope patterns for the different charge states observed from **C_{60}@G2** in DMF (PF_6^- as counterion).

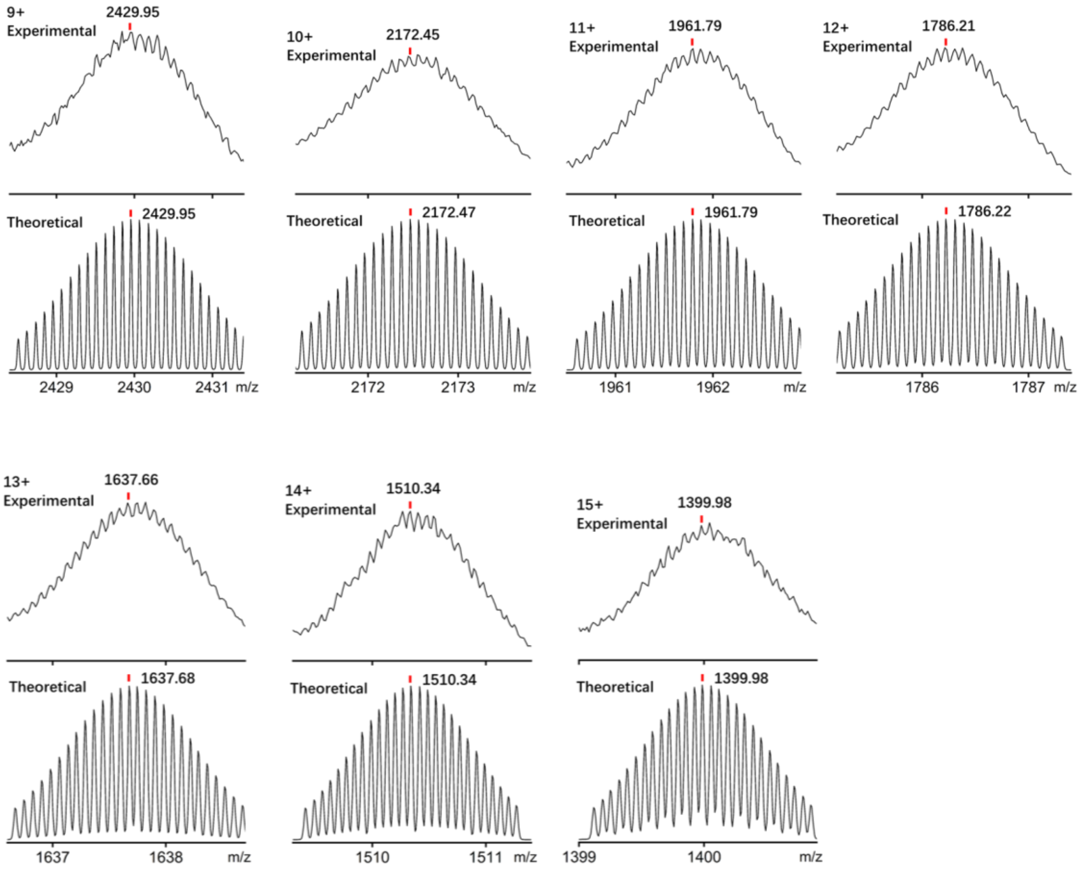

Figure S104. Theoretical (bottom) and experimental (top) isotope patterns for the different charge states observed from **$(C_{60})_2$@G2** in DMF (PF_6^- as counterion).

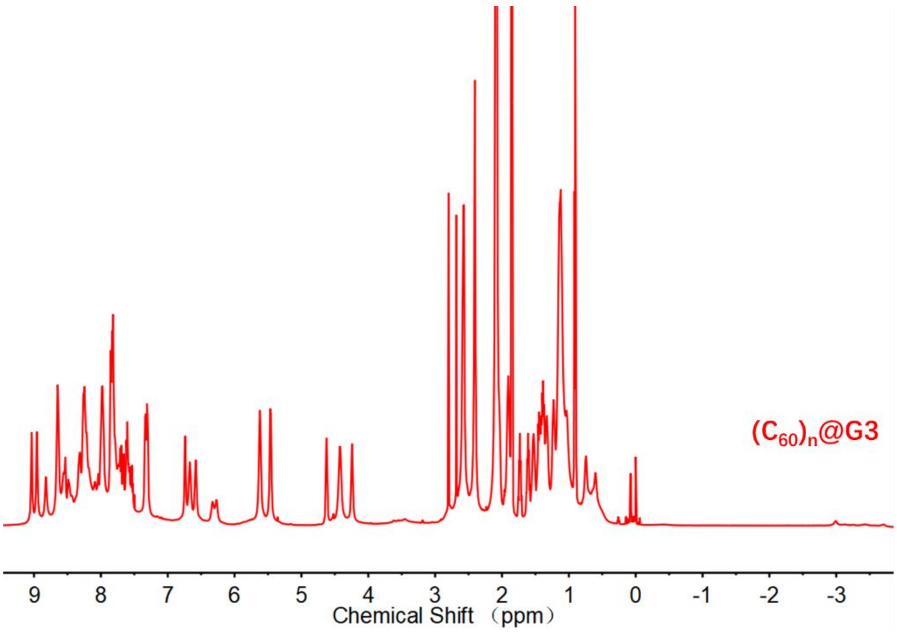

Figure S105. ^{1}H NMR spectrum of compound **$(C_{60})_n$@G3** in CD_3CN.

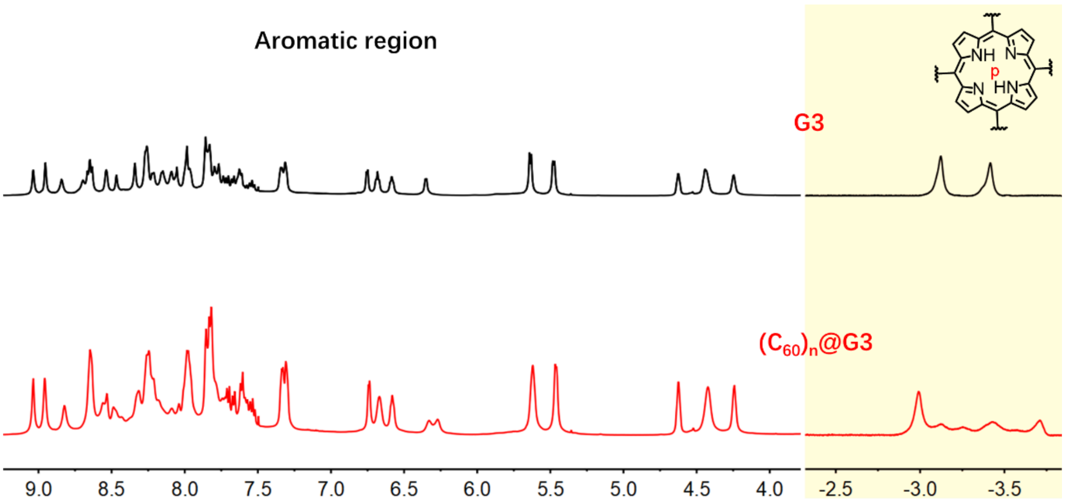

Figure S106. Comparison diagram of ^{1}H NMR spectra (600 M) of **G3** and **$(C_{60})_n$@G3** (the pink part was enlarged).

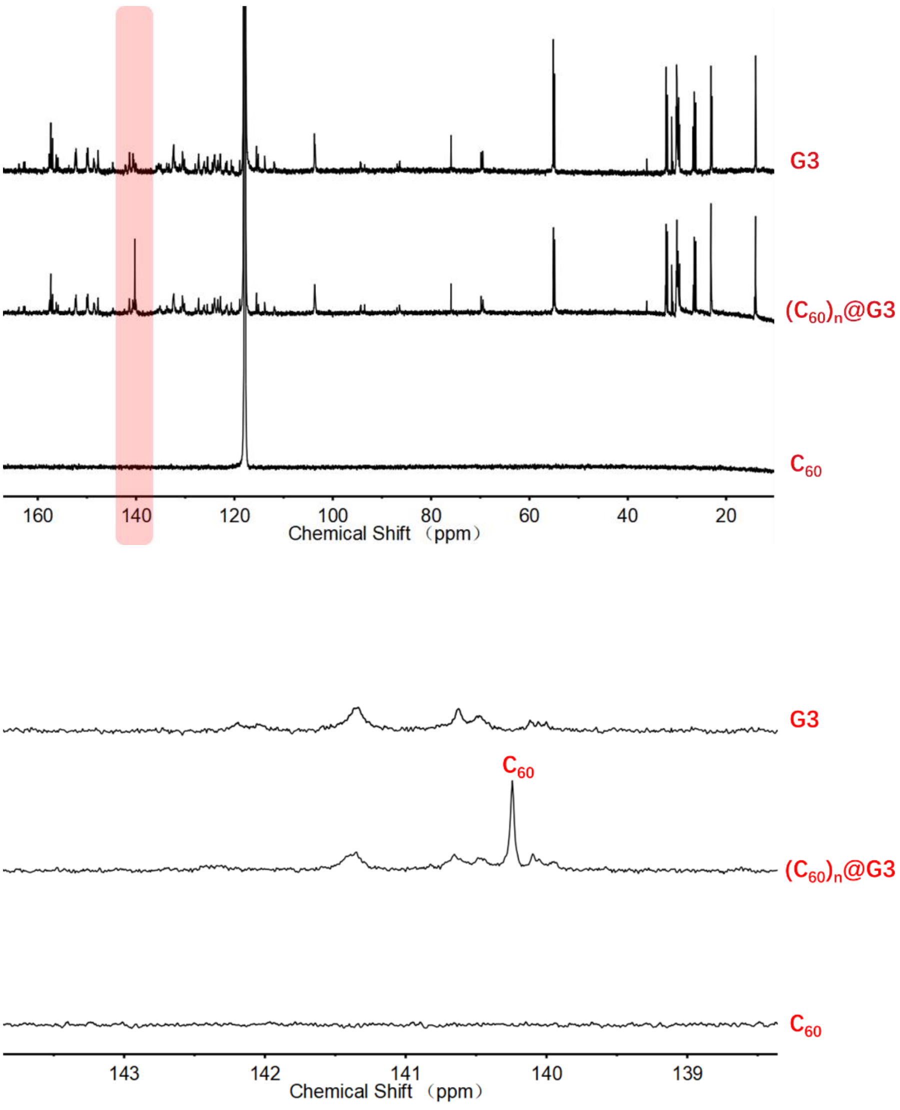

Figure S107. Comparison diagram of ^{13}C NMR spectra (600 M) of **G3**, $(C_{60})_n$**@G3** and C_{60} in CD_3CN.

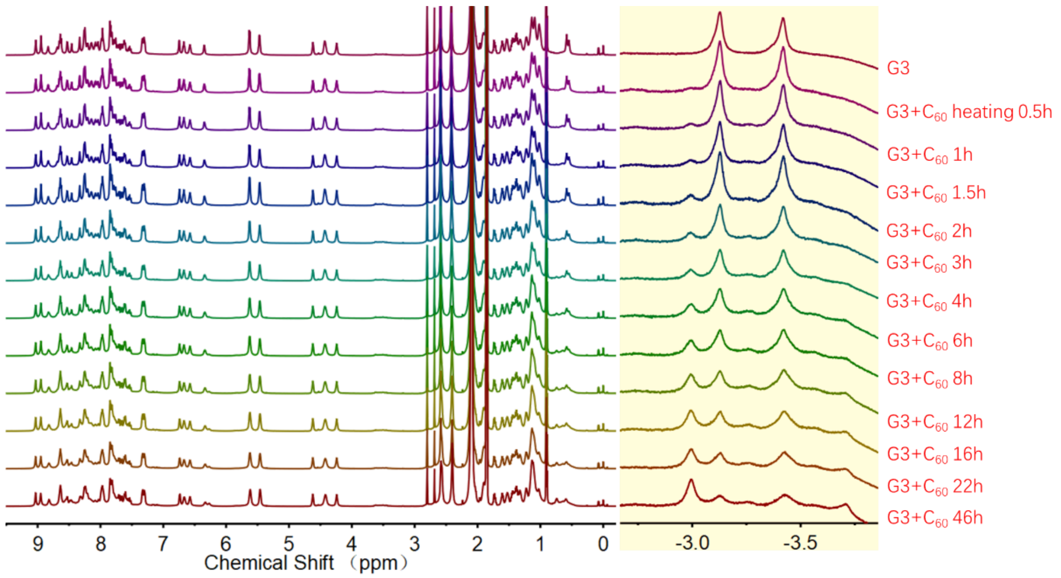

Figure S108. ^{1}H NMR spectra (600 M) monitoring of the process of **G3** wrapping C_{60} under 353K heating conditions (the pink part was enlarged).

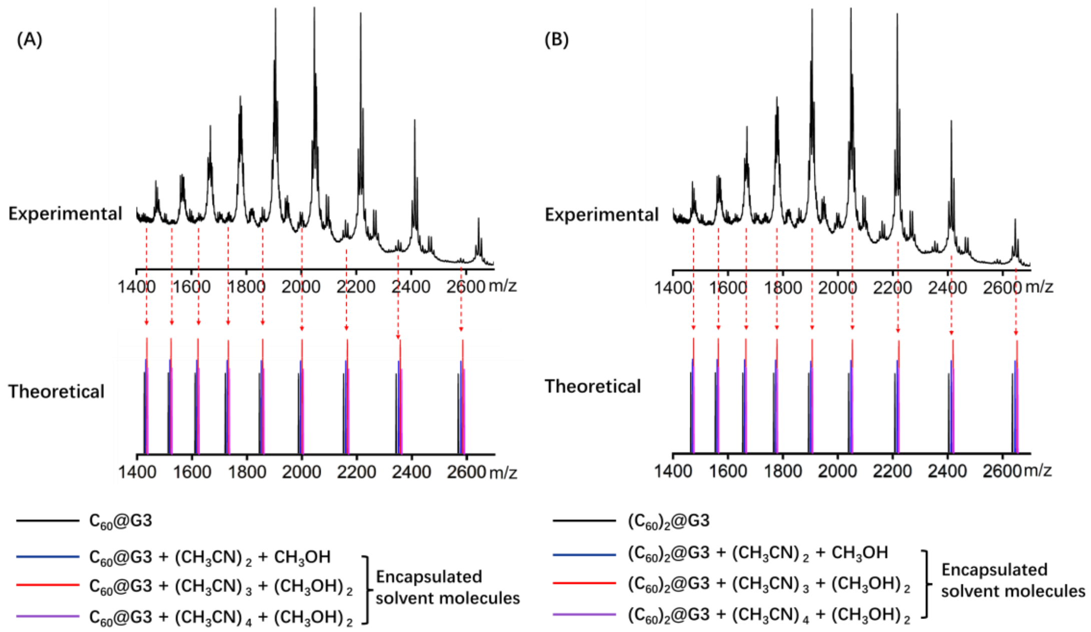

Figure S109. Experimental and theoretical values of mass spectrometry data for (A) part C_{60}@**G3** and (B) part $(C_{60})_2$@**G3**.

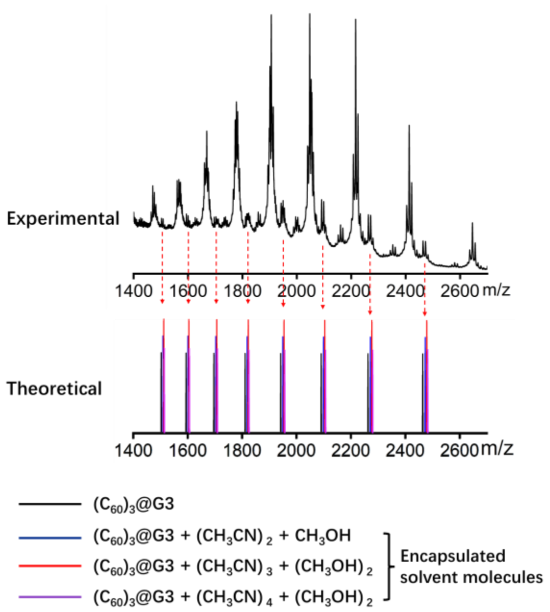

Figure S110. Experimental and theoretical values of mass spectrometry data for part $(C_{60})_3$@**G3**.

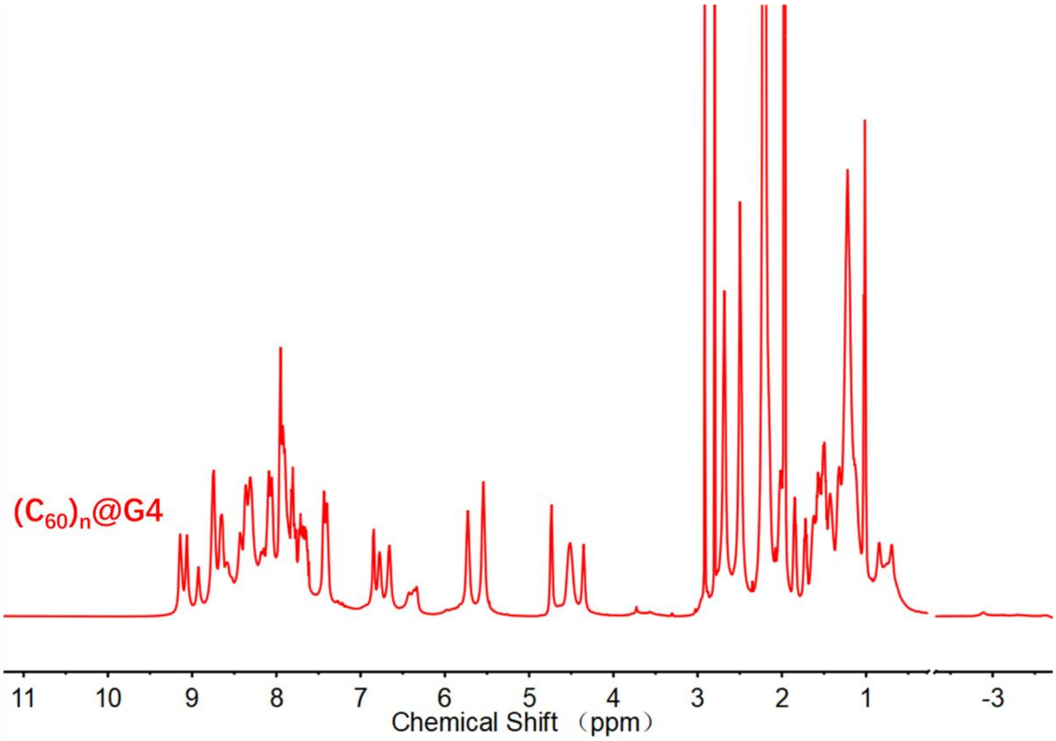

Figure S111. ^{1}H NMR spectrum of compound **$(C_{60})_n$@G4** in CD_3CN.

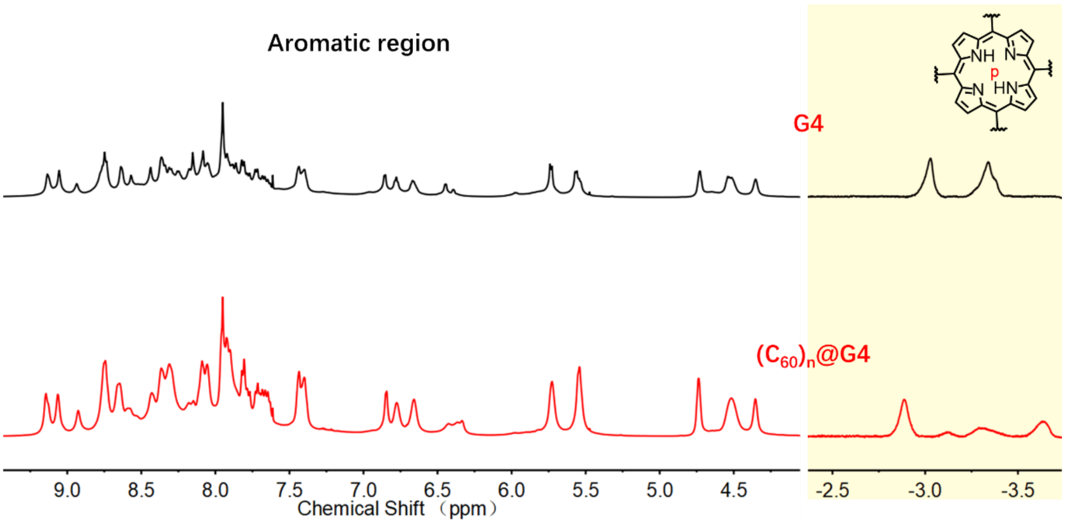

Figure S112. Comparison diagram of ^{1}H NMR spectra (600 M) of **G4** and **$(C_{60})_n$@G4** (the pink part was enlarged).

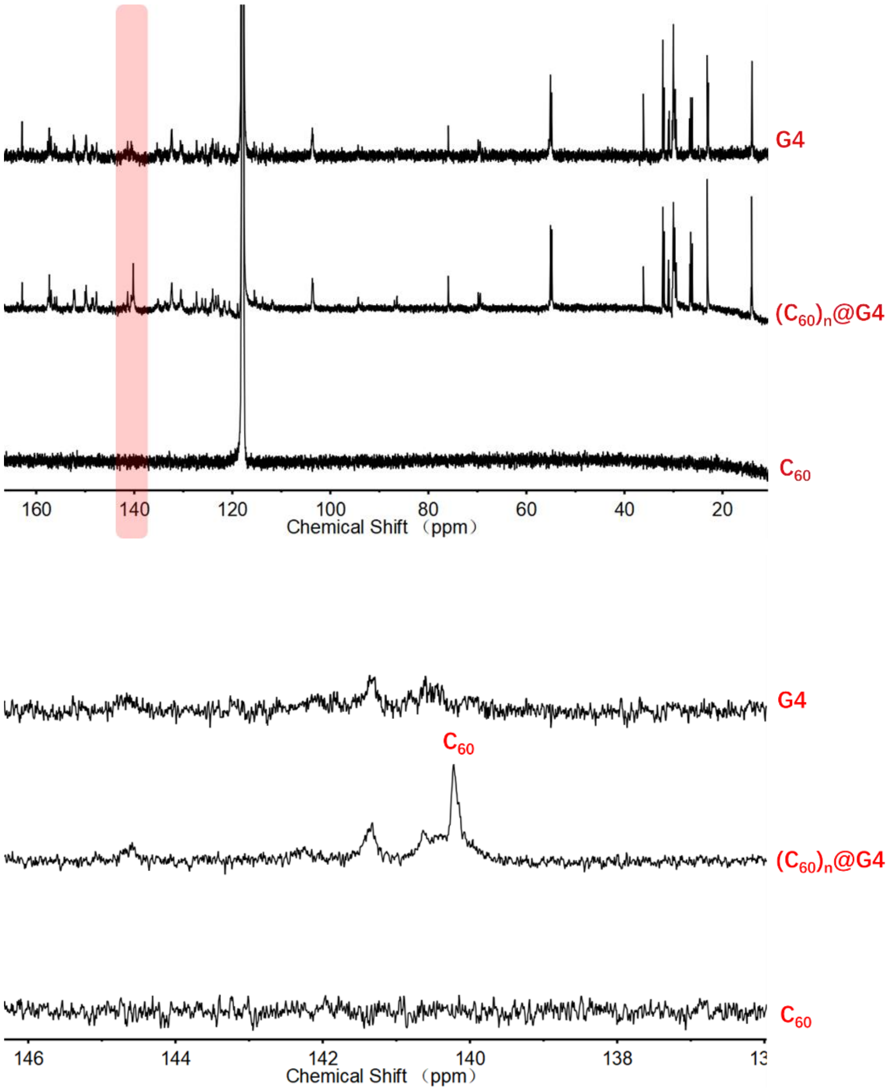

Figure S113. Comparison diagram of ^{13}C NMR spectra (600 M) of **G4**, $(C_{60})_n$**@G4** and C_{60} in CD_3CN.

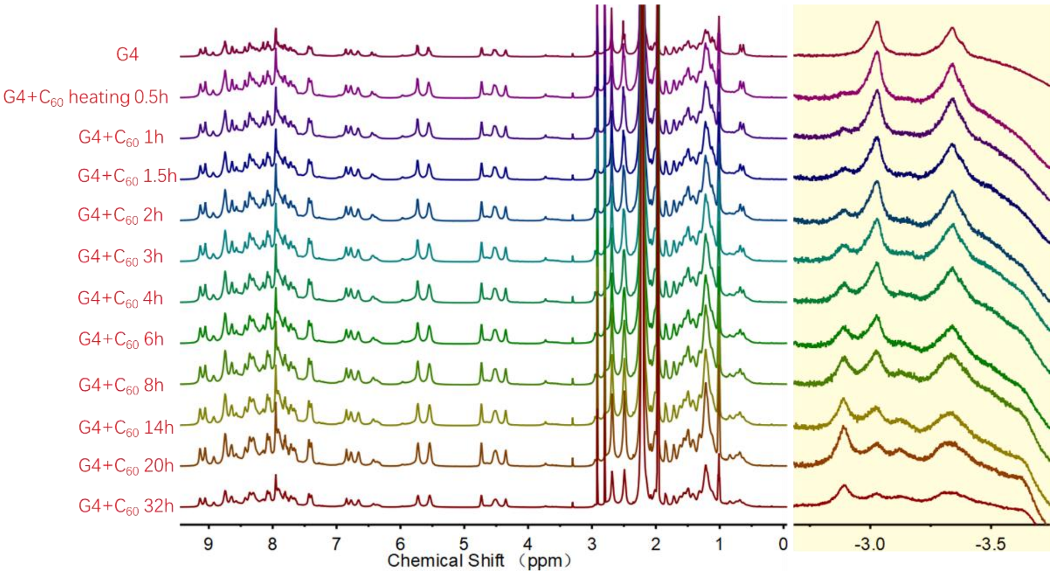

Figure S114. ^{1}H NMR spectra (600 M) monitoring of the process of **G4** wrapping C_{60} under 353K heating conditions (the pink part was enlarged).

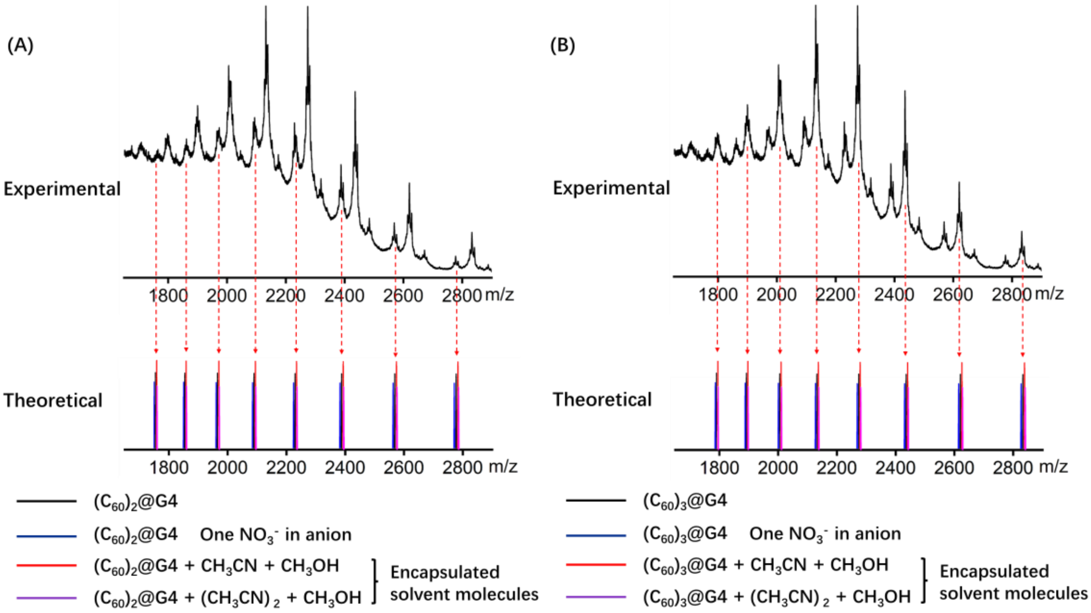

Figure S115. Experimental and theoretical values of mass spectrometry data for (A) part $(C_{60})_2$@**G4** and (B) part $(C_{60})_3$@**G4**.

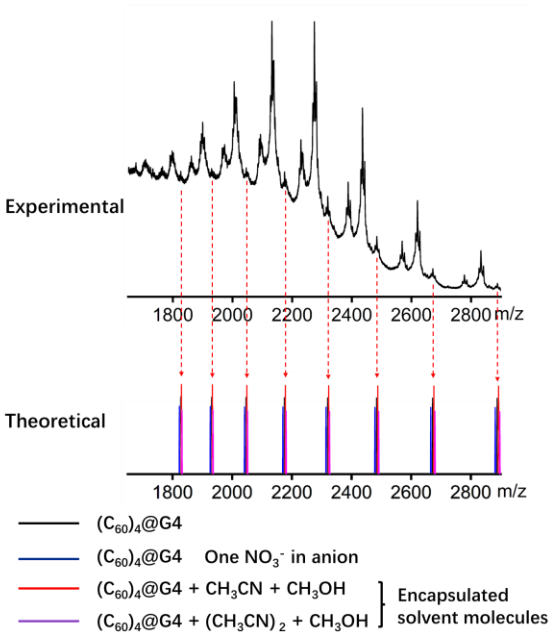

Figure S116: Experimental and theoretical values of mass spectrometry data for part $(C_{60})_4$@**G4**.

7. Calculation of binding constant (K_a), activation energy (E_a) and the energy state of host-guest complexes.

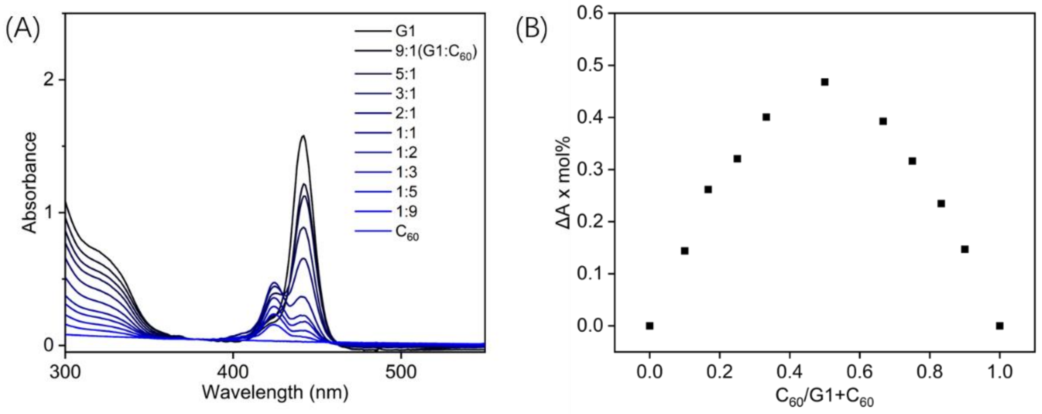

Figure S117. (A) UV-vis absorption of **G1** with guest C_{60} in different molar ratios (**G1** + C_{60} = 4 μM). (B) Job's plot of the **G1**$\supset C_{60}$ in DMF, showing a 1:1 stoichiometry.

UV-Vis Titration of G1 and G2 with C_{60}

The binding constant (K_a) for the formation of **C_{60}@G1, C_{60}@G2** and **$(C_{60})_2$@G2** in DMF was determined by UV titration on a Hitachi (model U-3010) UV–vis spectrophotometer in a 1-cm UV cuvette. The concentrated solution of C_{60} (1 mM in PhMe) was added incrementally to the Metal-organic capsules solution (20 μM in DMF). The UV-Vis spectra were recorded one after the other. From a global shift analysis of the titration data in absorption intensity at 401 nm, 433 nm and 466 nm using Bindfit [S3,S4] gave an association constant of $(2.9 \pm 0.6) \times 10^4$ M^{-1}, $(3.06 \pm 0.5) \times 10^4$ M^{-1} and $(1.99 \pm 0.5) \times 10^4$ $M^{-1}$for **C_{60}@G1, C_{60}@G2** and **$(C_{60})_2$@G2**, respectively.

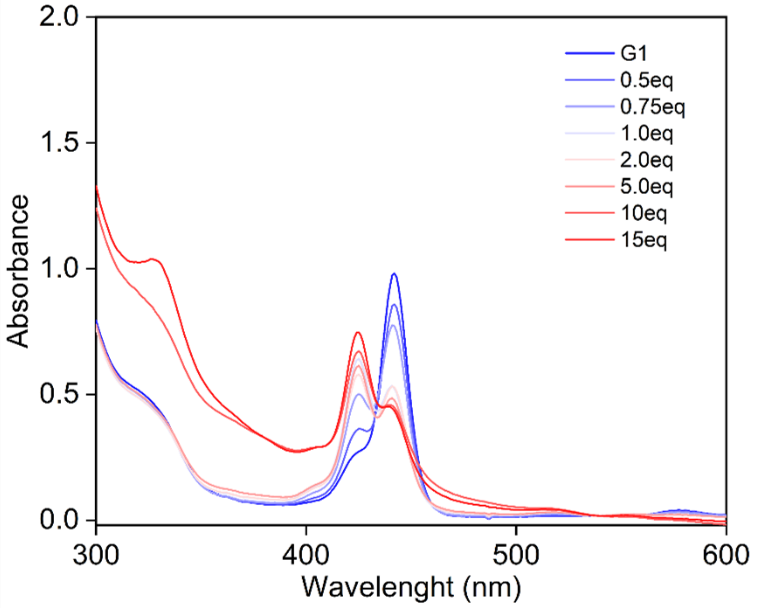

Figure S118. UV-Vis titration of **C_{60}** into **G1** in DMF.

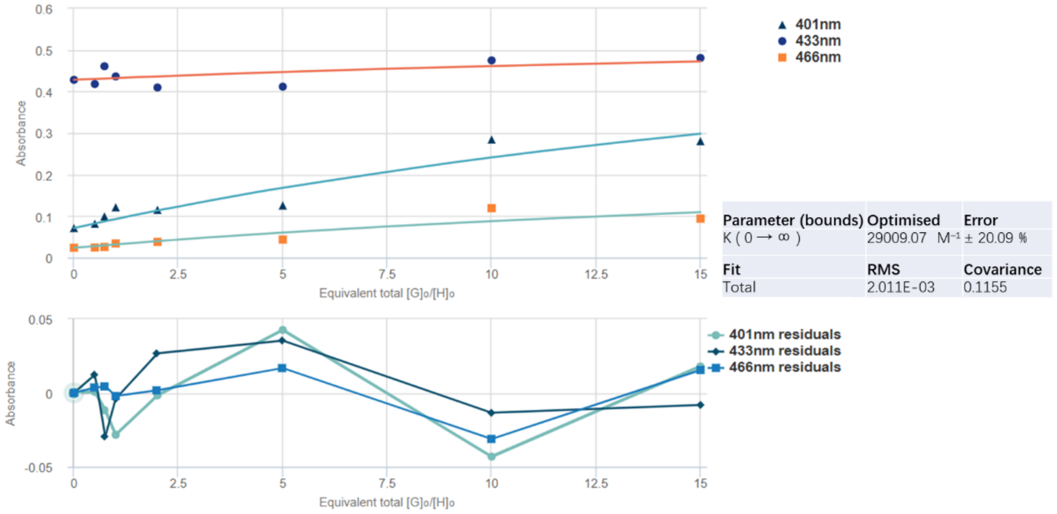

Figure S119. Binding isotherms (1:1 model) fitted to the absorbance shift of three bands vs. the equivalents of C_{60} added to determine the binding affinity of **G1** with C_{60} (top); and the residual plot from the fit (bottom).

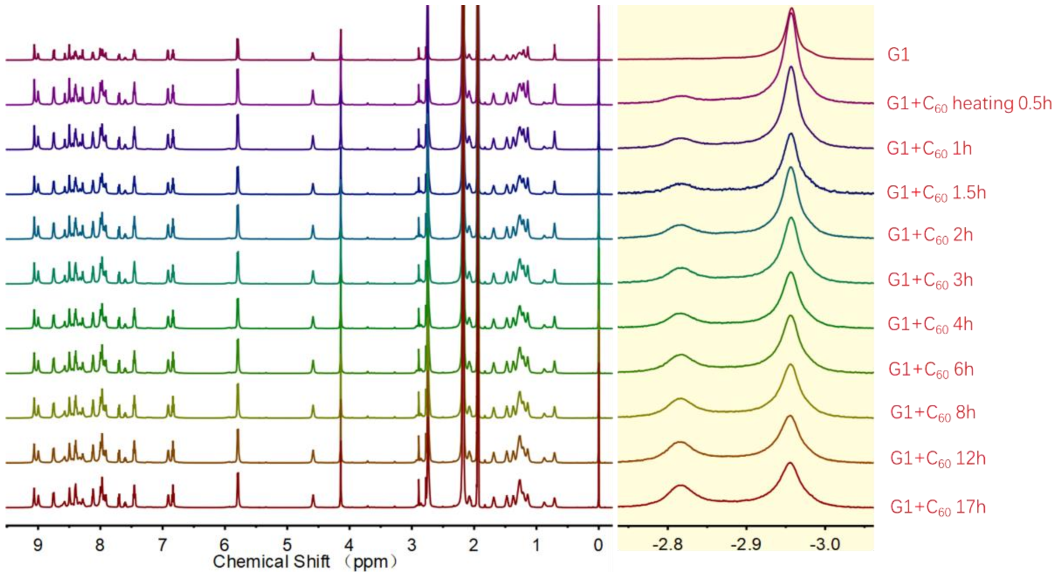

Figure S120. ^{1}H NMR spectra (600 M) monitoring of the process of **G1** wrapping C_{60} under 333K heating conditions (the pink part was enlarged).

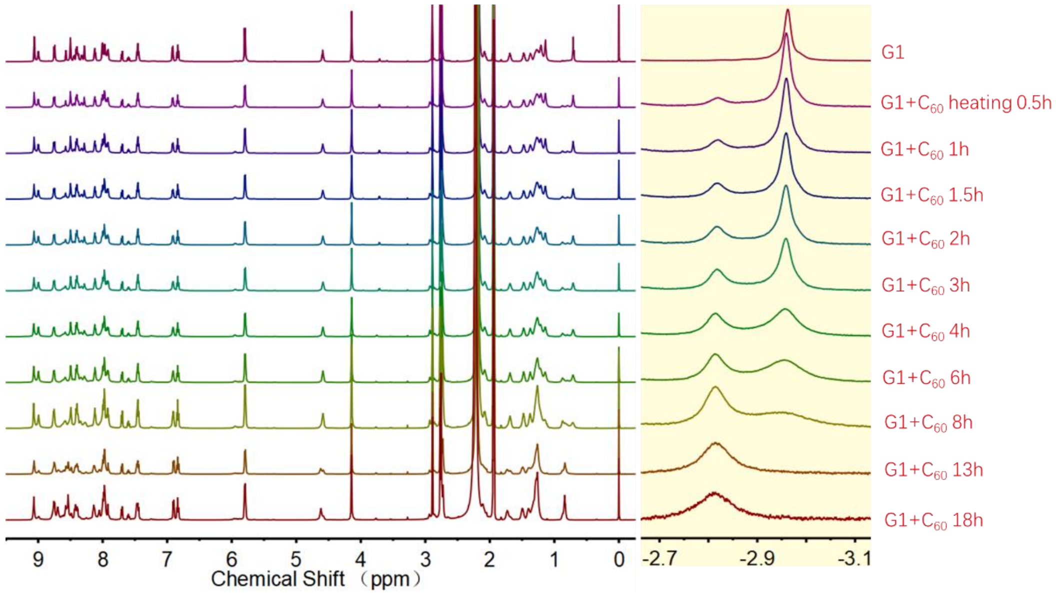

Figure S121. ^{1}H NMR spectra (600 M) monitoring of the process of **G1** wrapping C_{60} under 353K heating conditions (the pink part was enlarged).

Table S1. The integral ratio (**G1**:C_{60}@**G1**) of proton (H^P) in porphyrin rings during ^{1}H NMR spectra (600 M) monitoring of the process of **G1** wrapping C_{60} under 333K and 353K heating conditions.

Integral ratio	Time (h)										
	0.5	1	1.5	2	3	4	6	8	12	13	17
G1 : C_{60}@G1 (333 K)	8.33	5.56	4.35	3.57	3.0	2.86	2.38	2.2	1.9	-	1.7
G1 : C_{60}@G1 (353 K)	5.8	4.65	3.42	2.53	2.14	1.56	1.2	0.59	-	0	-

Table S2. According to the integration ratio in Table S1 and c_0=0.581 mM calculated $\ln c_t$ corresponding to different heating times at 333 K and 353 K.

Time (s)	1800	3600	5400	7200	10800	14400	21600
$\ln c_t$ (333 K)	-7.5636	-7.615	-7.6564	-7.6974	-7.7356	-7.7494	-7.8018
$\ln c_t$ (353 K)	-7.6089	-7.6459	-7.7063	-7.7824	-7.8341	-7.9462	-8.0566

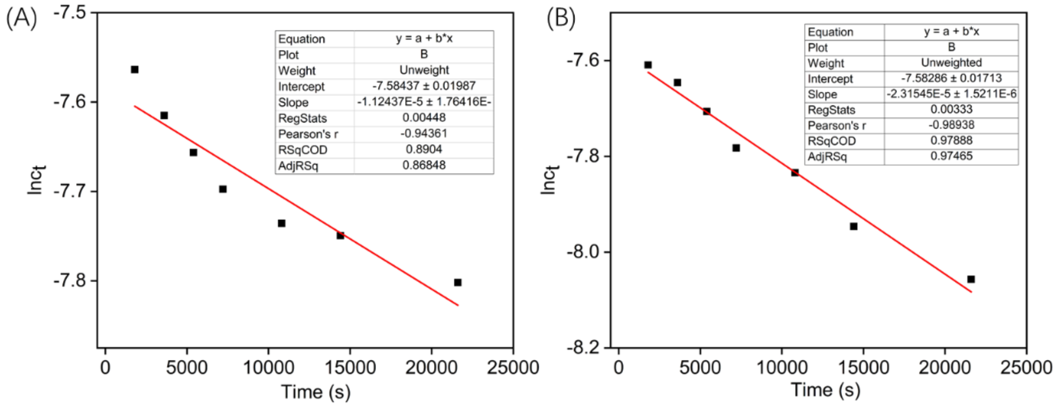

Figure S122. Based on the kinetic equations of the first order reaction ($\ln c_t = \ln c_0 - kt$), the rate constants (k) at the two temperatures were obtained by fitting straight lines. (A) Experiments conducted at 333K, (B) Experiments conducted at 353K.

$$G1 \xrightarrow[C_{60}]{k \text{ (rate constant)}} C_{60}@G1$$

$t = 0 \qquad c_0 = 0.581\ \text{mM} \qquad c_{P,0}$

$t = t \qquad c_t \qquad c_p$

Kinetic equation: $\ln c_t = \ln c_0 - kt$ (1)

$k_1 = k_{(333\ K)} = 1.12437E\text{-}5\ s^{-1}$

$k_2 = k_{(353\ K)} = 2.31545E\text{-}5\ s^{-1}$

$R = 8.314\ J{\cdot}mol^{-1}{\cdot}K^{-1}$

Arrhenius formula: $\ln\frac{k_2}{k_1} = \frac{E_a}{R}\frac{(T_2 - T_1)}{T_1 T_2}$ (2)

Activating energy: $E_a = 35.32\ kJ\ {\cdot}mol^{-1}$

Figure S123. Calculation of E_a for **G1** encapsulated C_{60} according to the Arrhenius formula.

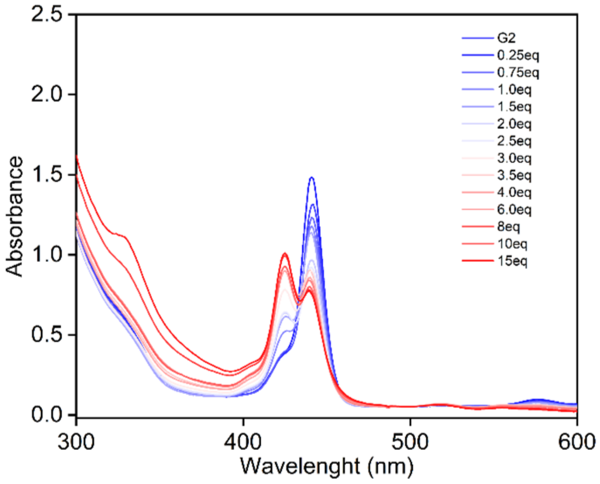

Figure S124. UV-Vis titration of **C_{60}** into **G2** in DMF.

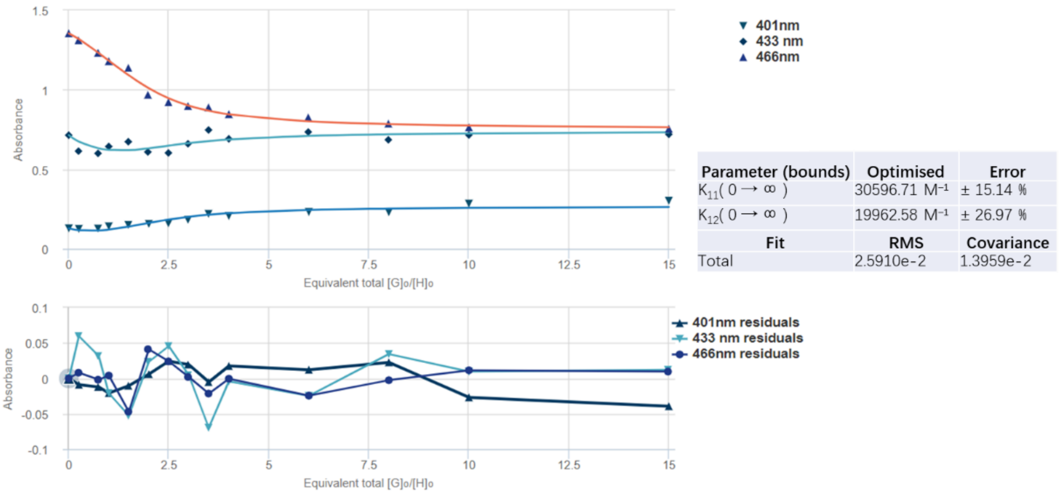

The cooperativity parameter (α) of **G2** wrapping C_{60} is described as: $\alpha = K_2/K_1 = (3.06 \pm 0.5) \times 10^4/(1.99 \pm 0.5) \times 10^4 = 0.65 < 1$

Where $\alpha > 1$ is positive cooperativity and $\alpha < 1$ is negative cooperativity. [S5]

Figure S125. Binding isotherms (1:2 model) fitted to the absorbance shift of three bands vs. the equivalents of C_{60} added to determine the binding affinity (top); and the residual plot from the fit (bottom) as well as the calculation of cooperativity parameter (α).

Calculation of the energy state of the host-guest complexes

To obtain interaction between C_{60} and supramolecules, we performed a systematic optimization protocol including forcefield and semiempirical calculations using xtb 6.5.0. [S6] First, the geometric structure for each cluster were preoptimized at the GFN-FF and GFN0-xTB method. [S7-S8] The obtained structure was further optimized using the semiempirical quantum mechanical GFN1-xTB method. [S9] (The saturated fatty chain in **G2-G4** was simplified to methoxy group).

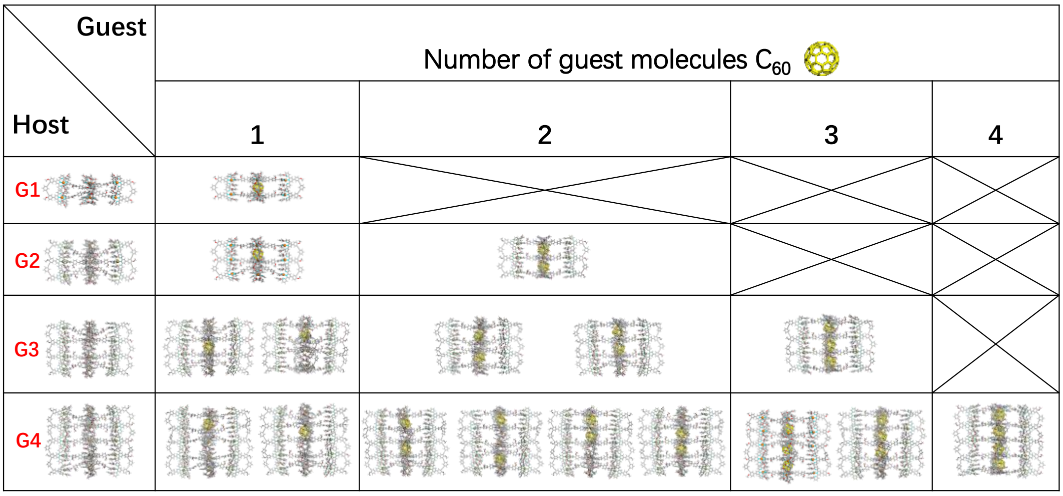

Figure S126. Possible structures after host-guest interactions between **G1** to **G4** and excessive C_{60}.

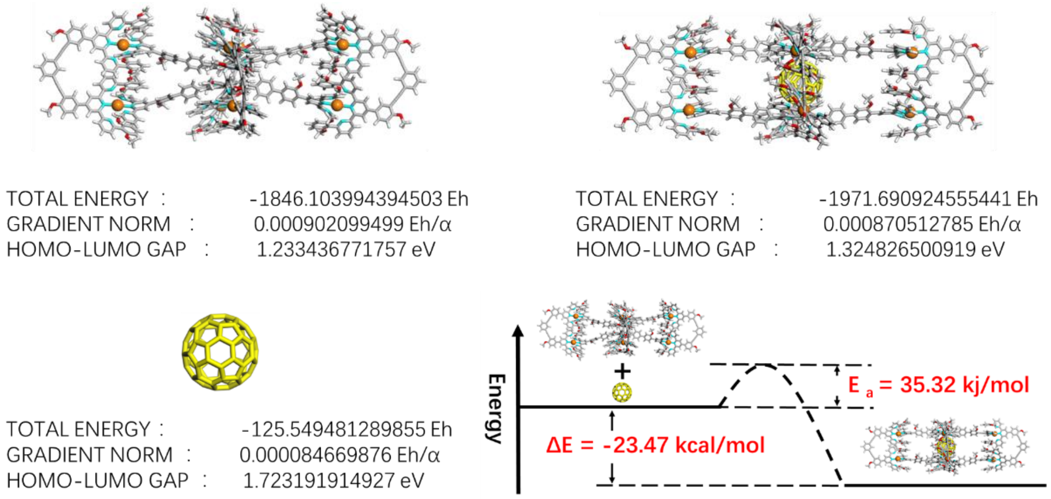

Figure S127. The total energy of **G1**, C_{60}@**G1** and free C_{60} were calculated through GFN1-xTB, and the schematic diagram of total energy change (ΔE) in the process of **G1** wrapping C_{60}. (1 Eh = 627.51 kcal/mol; activation energy Ea was calculated according to Figure S115 and S116)

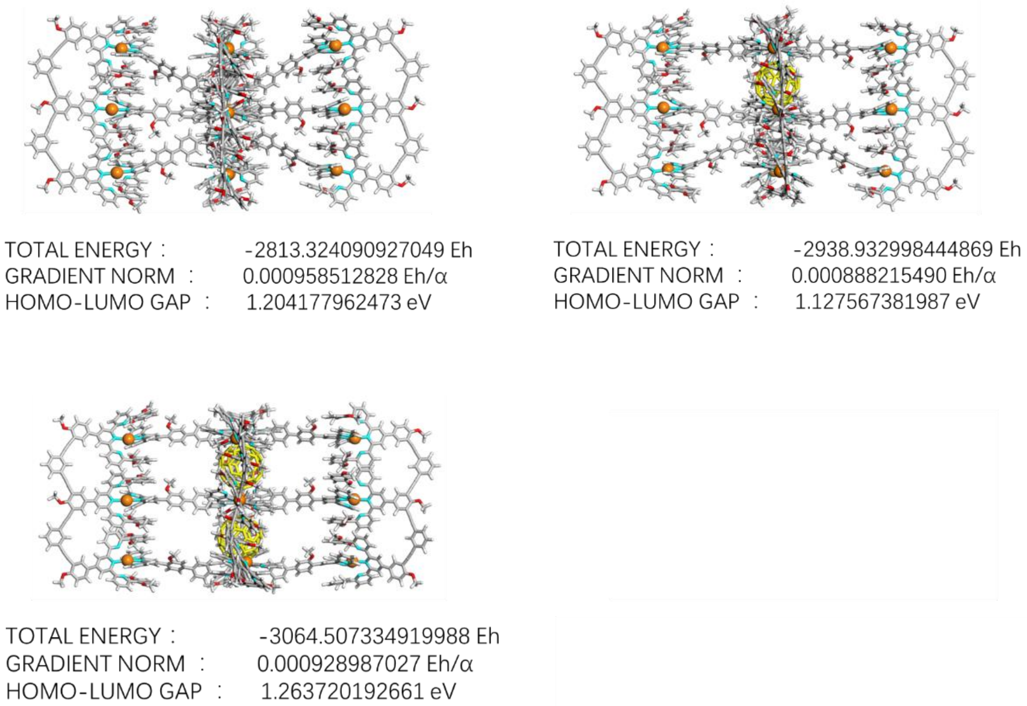

Figure S128. The energy states of **G2**, C_{60}@**G2** and $(C_{60})_2$@**G2** were calculated through GFN1-xTB.

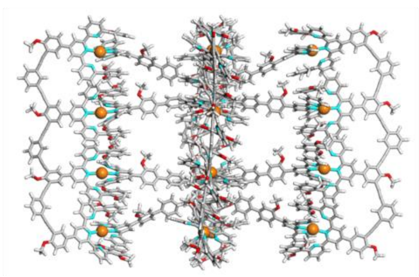

TOTAL ENERGY： -3780.534244146036 Eh
GRADIENT NORM ： 0.000965866563 Eh/α
HOMO-LUMO GAP ： 1.226839006413 eV

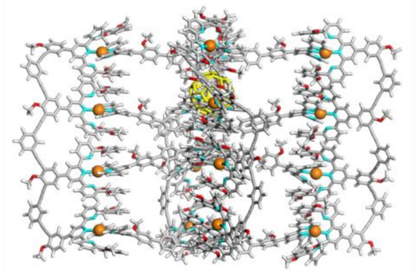

TOTAL ENERGY： -3906.129291778843 Eh
GRADIENT NORM ： 0.000673725244 Eh/α
HOMO-LUMO GAP ： 1.153631490891 eV

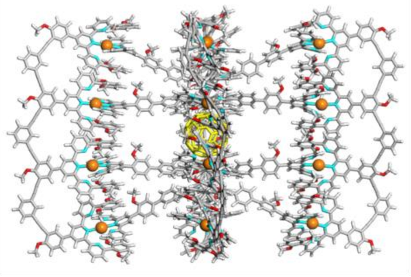

TOTAL ENERGY： -3906.160044057117 Eh
GRADIENT NORM ： 0.000934230343 Eh/α
HOMO-LUMO GAP ： 1.198578204357 eV

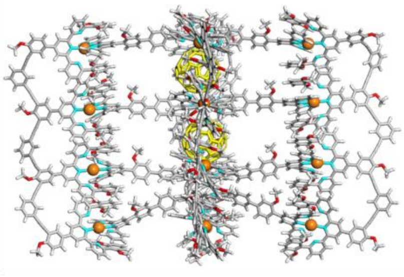

TOTAL ENERGY： -4031.754725999013 Eh
GRADIENT NORM ： 0.000737533431 Eh/α
HOMO-LUMO GAP ： 1.217912759524 eV

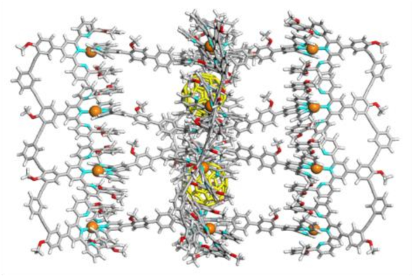

TOTAL ENERGY： -4031.751628181126 Eh
GRADIENT NORM ： 0.000943207022 Eh/α
HOMO-LUMO GAP ： 1.120770307700 eV

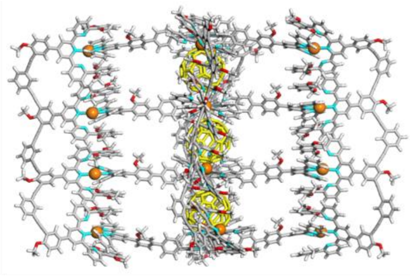

TOTAL ENERGY： -4157.343145420004 Eh
GRADIENT NORM ： 0.000777817993 Eh/α
HOMO-LUMO GAP ： 1.266380085963 eV

Figure S129. The energy states of **G3**, C_{60}@**G3**, $(C_{60})_2$@**G3**, $(C_{60})_3$@**G3** and their isomers were calculated by GFN1-xTB.

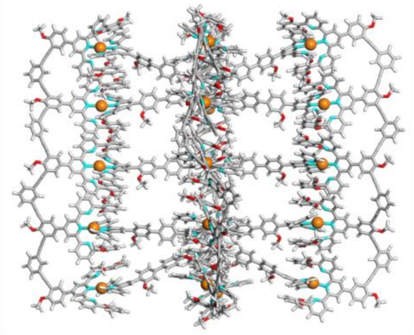

TOTAL ENERGY： -4747.720659267078 Eh
GRADIENT NORM ： 0.000917149387 Eh/α
HOMO-LUMO GAP ： 1.188380806104 eV

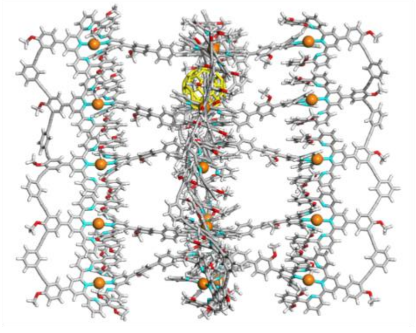

TOTAL ENERGY： -4873.341713829346 Eh
GRADIENT NORM ： 0.000979587293 Eh/α
HOMO-LUMO GAP ： 1.220356969356 eV

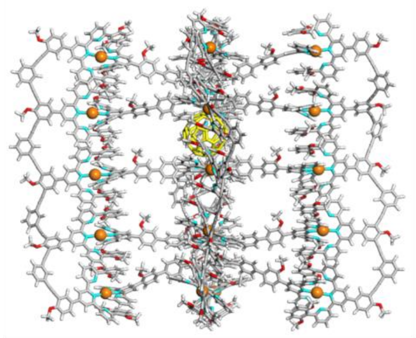

TOTAL ENERGY： -4873.365396158342 Eh
GRADIENT NORM ： 0.000997319748 Eh/α
HOMO-LUMO GAP ： 1.226305780489 eV

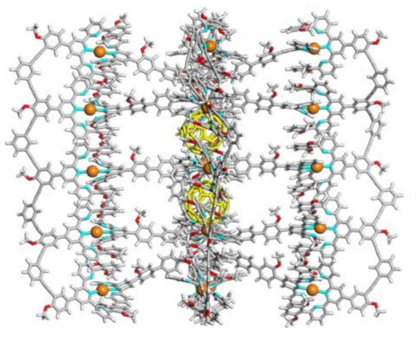

TOTAL ENERGY： -4999.001964146004 Eh
GRADIENT NORM ： 0.000999867439 Eh/α
HOMO-LUMO GAP ： 1.226251345097 eV

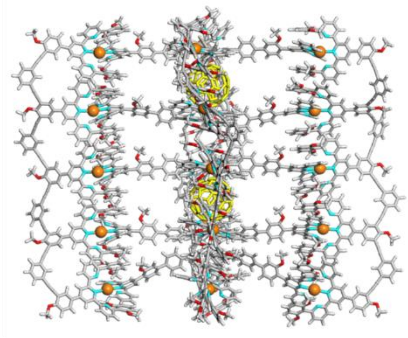

TOTAL ENERGY： -4998.936285779753 Eh
GRADIENT NORM ： 0.000848218445 Eh/α
HOMO-LUMO GAP ： 1.216063461570 eV

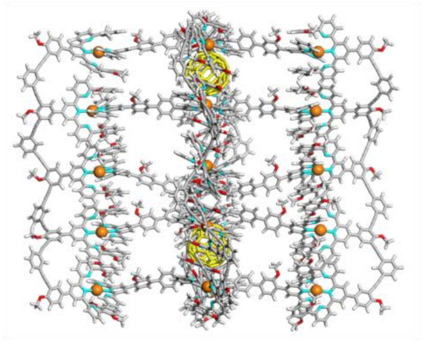

TOTAL ENERGY： -4998.942239875352 Eh
GRADIENT NORM ： 0.000747390472 Eh/α
HOMO-LUMO GAP ： 1.144899376975 eV

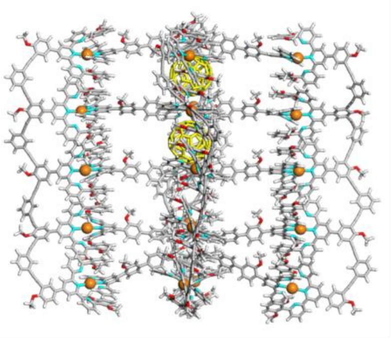

TOTAL ENERGY： -4998.944138010906 Eh
GRADIENT NORM ： 0.000902564593 Eh/α
HOMO-LUMO GAP ： 1.222442145196 eV

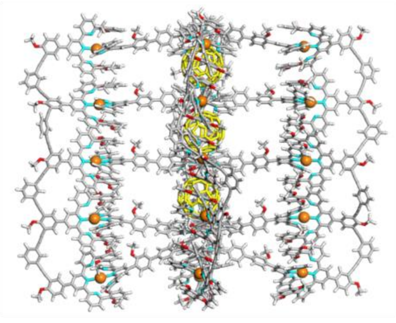

TOTAL ENERGY： -5124. 585032104970 Eh
GRADIENT NORM ： 0.000936973429 Eh/α
HOMO-LUMO GAP ： 1.213371919010 eV

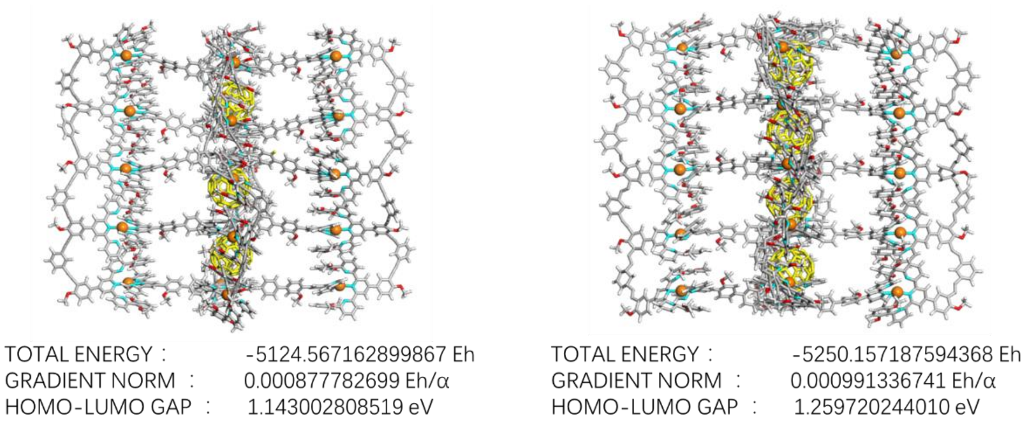

Figure S130. The energy states of **G4**, C_{60}@**G4**, $(C_{60})_2$@**G4**, $(C_{60})_3$@**G4**, $(C_{60})_4$@**G4** and their isomers were calculated by GFN1-xTB.

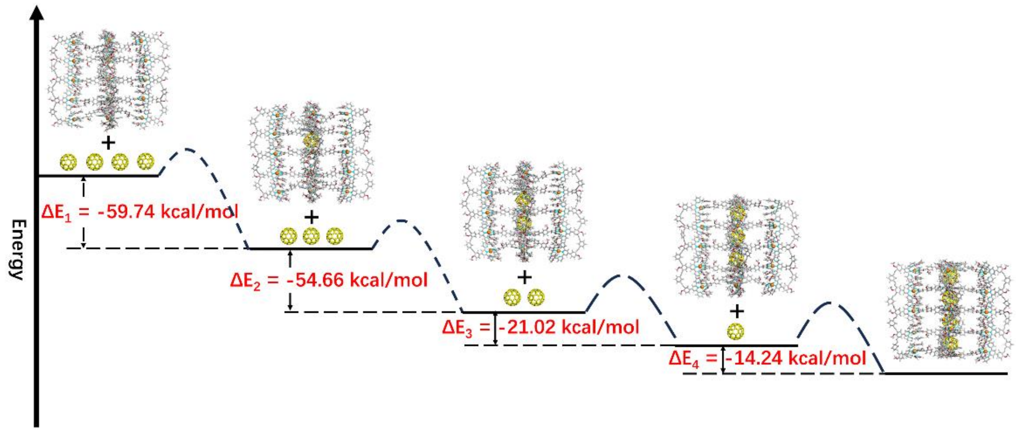

Figure S131. The energy schematic diagram of inferred main path and corresponding binding energy for **G4** wrapping C_{60}, respectively (the main path given by calculating the maximum energy difference).

8. Comparison of the properties of capsules loaded with C_{60} to produce $^1O_2$.

Capsules loaded with C_{60} were tested to generate singlet oxygen. 9,10-diphenylanthracene (DPHA) was used the $^1O_2$ scavenger monitors $^1O_2$ generation, capsules loaded with C_{60} complexes were used as a photocatalyst to dissolve in acetonitrile solution and detect the changes in absorption intensity of DPHA by UV at different irradiation times. This gradual decrease in absorption intensity can be ascribed to the formation of DPHA endoperoxide via singlet oxygen mediated oxidation of DPHA.

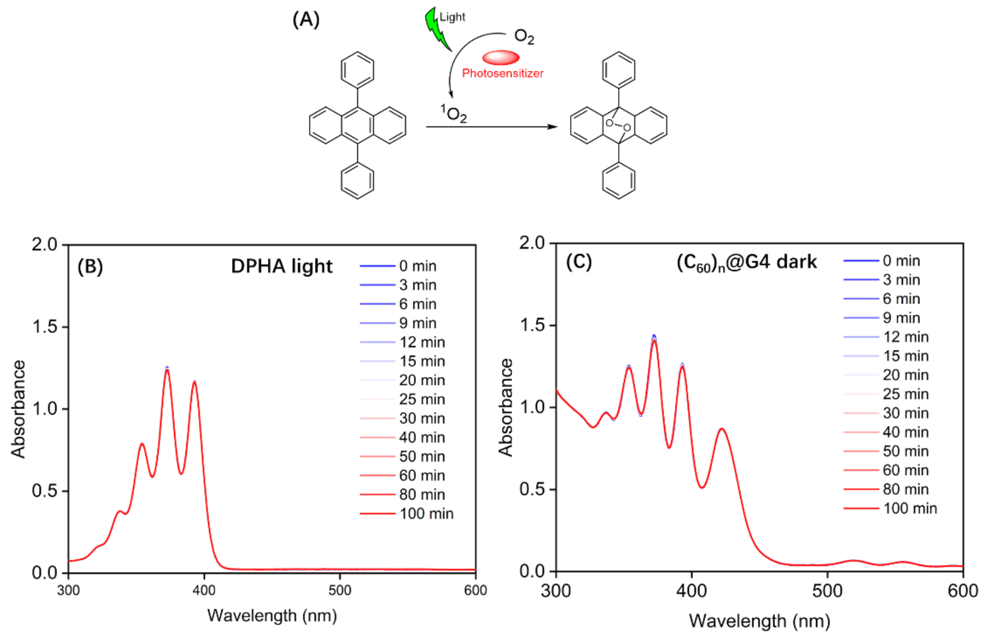

Figure S132. (A) The mechanism of 9,10-diphenylanthracene (DPHA) as the $^1O_2$ scavenger monitors singlet oxygen generation in the solution. (B) The absorption spectra of DPHA (106.0 μM) after irradiation (405 nm, 20 mW/cm^2) for different time no photosensitizer added. (C) The absorption spectra of DPHA (106.0 μM) under dark conditions for different time in the presence of $(C_{60})_n$@**G4** (1.0 μM).

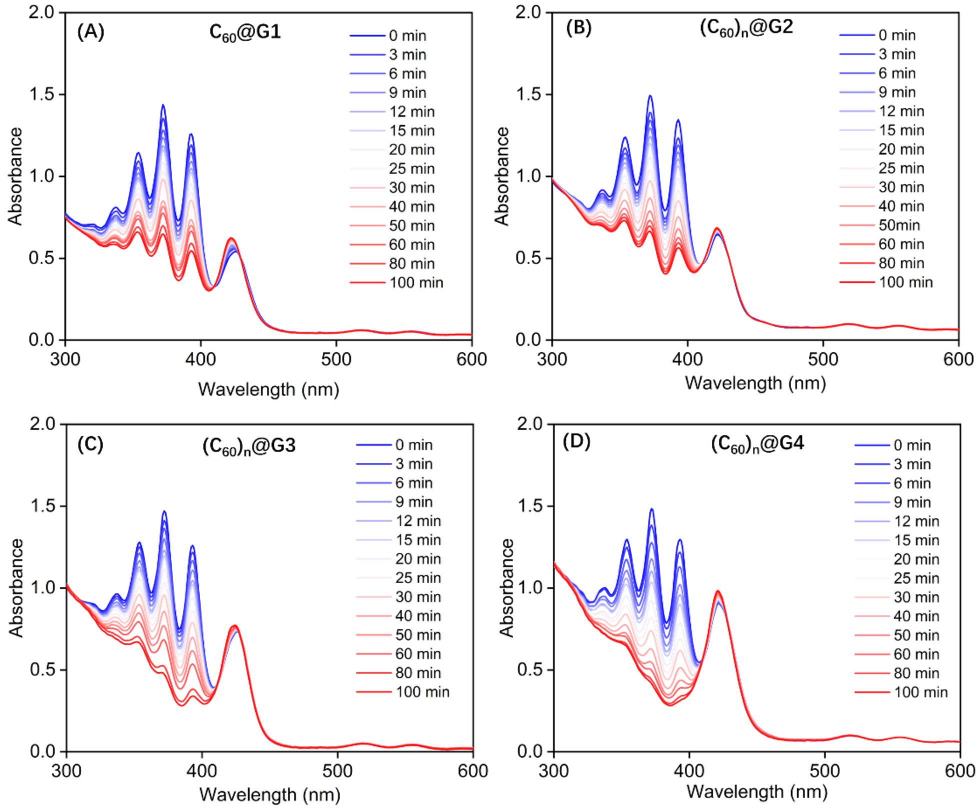

Figure S133. The absorption spectra of DPHA (106.0 μM) after irradiation (405 nm, 20 mW/cm^2) for different time in the presence of (A) C_{60}@**G1** (1.0 μM), (B) $(C_{60})_n$@**G2** (1.0 μM), (C) $(C_{60})_n$@**G3** (1.0 μM) and (D) $(C_{60})_n$@**G4** (1.0 μM) in CH_3CN.

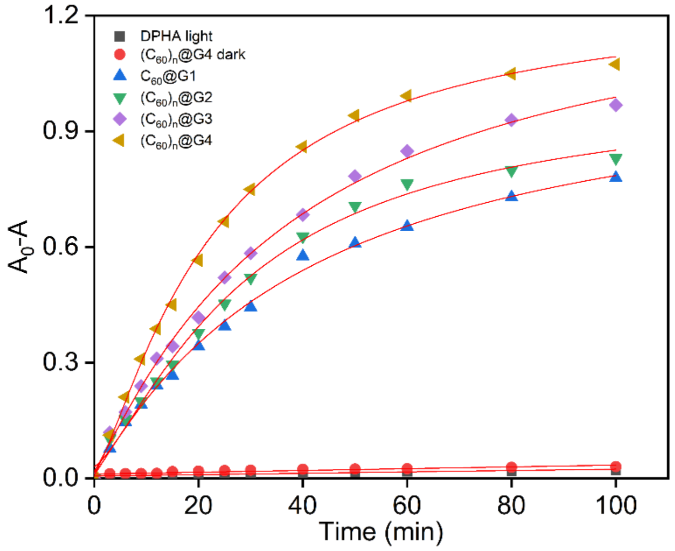

Figure S134. Two comparison samples and capsule encapsulated C_{60} complex [C_{60}@**G1**, $(C_{60})_n$@**G2**, $(C_{60})_n$@**G3** and $(C_{60})_n$@**G4**)] plots for the absorption decays of DPHA (106.0 μM) at 373 nm.

9. References.

S1. Lv, Z.; Zou, L.; Wei, H.; Liu, S.; Huang, W.; Zhao, Q., Phosphorescent Starburst Pt(II) Porphyrins as Bifunctional Therapeutic Agents for Tumor Hypoxia Imaging and Photodynamic Therapy. *ACS Appl. Mater. Interfaces.* **2018**, *10*, 19523-19533.

S2. Wang. S.; Fu. J.; Liang. Y.; He. Y.; Chen. Y.; Chan. Y., Metallo-Supramolecular Self-Assembly of a Multicomponent Ditrigon Based on Complementary Terpyridine Ligand Pairing. *J. Am. Chem. Soc.* **2016,** 138, 3651-3654.

S3. Thordarson, P., Determining association constants from titration experiments in supramolecular chemistry. *Chem. Soc. Rev.* **2011**, *40*, 1305-1323.

S4. Bindfit v0.5 (Open Data Fit, **2016**); http://app.supramolecular.org/bindfit/.

S5. Hunter. C.; Anderson. H., What is Cooperativity? *Angew. Chem. Int. Ed.* **2009,** *48*, 7488-7499.

S6. Bannwarth. C.; Caldeweyher. E., Ehlert. S.; Hansen. A.; Pracht. P.; Seibert. J.; Spicher. S.; Grimme. S., Extended tight-binding quantum chemistry methods. *WIREs Comput Mol Sci.* **2020,** *11*, e1493.

S7. Spicher. S.; Grimme. S., Robust Atomistic Modeling of Materials, Organometallic, and Biochemical Systems. *Angew. Chem. Int. Ed.* **2020,** *59*, 15665-15673.

S8. Grimme. S.; Bannwarth. C.; Shushkov. P., A Robust and Accurate Tight-Binding Quantum Chemical Method for Structures, Vibrational Frequencies, and Noncovalent Interactions of Large Molecular Systems Parametrized for All spd-Block Elements (Z = 1-86). *J. Chem. Theory Comput.* **2017,** *13*, 1989-2009.

S9. Bannwarth. C.; Ehlert. S.; Grimme. S., GFN2-xTB-An Accurate and Broadly Parametrized Self-Consistent Tight-Binding Quantum Chemical Method with Multipole Electrostatics and Density-Dependent Dispersion Contributions. *J. Chem. Theory Comput.* **2019,** *15*, 1652-1671.
